# Illuminating Neuropeptide Y Y_4_ Receptor Binding: Fluorescent Cyclic Peptides with Subnanomolar Binding Affinity as Novel Molecular Tools

**DOI:** 10.1021/acsptsci.4c00013

**Published:** 2024-03-20

**Authors:** Jakob Gleixner, Sergei Kopanchuk, Lukas Grätz, Maris-Johanna Tahk, Tõnis Laasfeld, Santa Veikšina, Carina Höring, Albert O. Gattor, Laura J. Humphrys, Christoph Müller, Nataliya Archipowa, Johannes Köckenberger, Markus R. Heinrich, Roger Jan Kutta, Ago Rinken, Max Keller

**Affiliations:** † Institute of Pharmacy, Faculty of Chemistry and Pharmacy, University of Regensburg, Universitätsstraße 31, D-93040 Regensburg, Germany; ‡ Institute of Chemistry, University of Tartu, Ravila 14a, 50411 Tartu, Estonia; § Institute of Biophysics and Physical Biochemistry, Faculty of Biology and Preclinical Medicine, University of Regensburg, Universitätsstraße 31, D-93040 Regensburg, Germany; ∥ Department of Chemistry and Pharmacy, Molecular and Clinical Pharmacy, Friedrich-Alexander-University Erlangen-Nürnberg, Nikolaus-Fiebiger-Straße 10, D-91058 Erlangen, Germany; ⊥ Institute of Physical and Theoretical Chemistry, Faculty of Chemistry and Pharmacy, University of Regensburg, Universitätsstraße 31, D-93053 Regensburg, Germany

**Keywords:** neuropeptide Y, pancreatic polypeptide, Y_4_ receptor, fluorescent ligand, binding assay, fluorescence microscopy

## Abstract

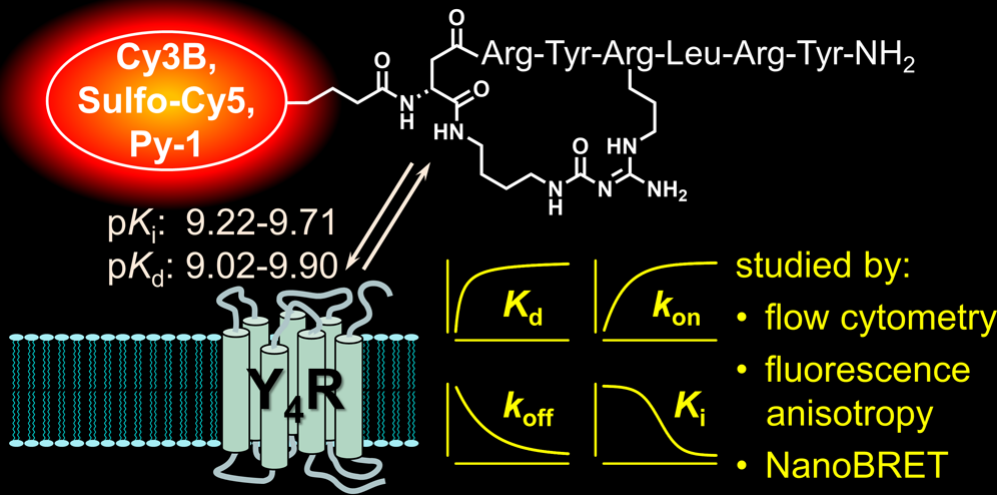

The neuropeptide Y (NPY) Y_4_ receptor (Y_4_R), a member of the family of NPY receptors, is physiologically activated by the linear 36-amino acid peptide pancreatic polypeptide (PP). The Y_4_R is involved in the regulation of various biological processes, most importantly pancreatic secretion, gastrointestinal motility, and regulation of food intake. So far, Y_4_R binding affinities have been mostly studied in radiochemical binding assays. Except for a few fluorescently labeled PP derivatives, fluorescence-tagged Y_4_R ligands with high affinity have not been reported. Here, we introduce differently fluorescence-labeled (Sulfo-Cy5, Cy3B, Py-1, Py-5) Y_4_R ligands derived from recently reported cyclic hexapeptides showing picomolar Y_4_R binding affinity. With p*K*_i_ values of 9.22–9.71 (radioligand competition binding assay), all fluorescent ligands (**16**–**19**) showed excellent Y_4_R affinity. Y_4_R saturation binding, binding kinetics, and competition binding with reference ligands were studied using different fluorescence-based methods: flow cytometry (Sulfo-Cy5, Cy3B, and Py-1 label), fluorescence anisotropy (Cy3B label), and NanoBRET (Cy3B label) binding assays. These experiments confirmed the high binding affinity to Y_4_R (equilibrium p*K*_d_: 9.02–9.9) and proved the applicability of the probes for fluorescence-based Y_4_R competition binding studies and imaging techniques such as single-receptor molecule tracking.

In humans, four functional neuropeptide Y (NPY) receptors (Y_1_R, Y_2_R, Y_4_R, Y_5_R), all members of the superfamily of G-protein-coupled receptors, mediate the action of the neuropeptides NPY, peptide YY (PYY), and pancreatic polypeptide (PP).^1^ Within the family of these linear 36-amino-acid peptides, PP exhibits a clear preference for the Y_4_R and is thus considered the primary activator of Y_4_R.^2^ PP and Y_4_R are involved in the regulation of food intake, pancreatic secretion, gastrointestinal motility, and anxiety.^3−13^ Therefore, the Y_4_R represents a potential target for the treatment of obesity and depression.^12−15^

Development processes aiming for new Y_4_R ligands require appropriate binding assays to determine Y_4_R affinities. So far, this has been primarily accomplished by radioligand competition binding studies using ^3^H- or ^125^I-labeled peptidic Y_4_R ligands. Although radiochemical binding assays represent powerful methods, in particular with respect to sensitivity and quantification of bound and free labeled ligand, several drawbacks are associated with the use of radioligands: special safety precautions, the need for specialized laboratories, permits for radioisotope handling, and the high cost of radiolabeled probes. These are some of the reasons for the continuously growing use of fluorescence-based techniques to study receptor–ligand binding. Moreover, most fluorescence-based binding assays allow, in contrast to radiochemical assays, investigations under homogeneous conditions, i.e., there is no need to separate free (unbound) ligand from receptor-bound ligand prior to measurement.^16−24^ In particular, this is advantageous for kinetic studies. Another factor promoting luminescence-based assays is the availability of instruments such as multimode plate readers allowing high-throughput measurements. A bottleneck with respect to the development and use of fluorescence-based binding assays is the limited availability of fluorescent ligands exhibiting, all at once, high receptor affinity, favorable physicochemical and photophysical properties, appropriate binding kinetics (reversible binding), and adequate chemical stability as well as photostability.

For the Y_4_R, few fluorescently labeled ligands have been reported, Sulfo-Cy5-labeled [K^4^]hPP,^25^ Sulfo-Cy5-labeled [K^4^,Nle^17,30^]hPP (**1**),^26^ 5-TAMRA-labeled [K^18^,Nle^17,30^]hPP (**2**),^27^ and the UR-KK236^28^-derived Sulfo-Cy5.5-labeled hexapeptide **3**(29) (for structures of **1**–**3** see Figure 1A). With a *K*_d_ value of 5.6 nM,^25^ only Sulfo-Cy5-[K^4^]hPP shows a dissociation constant <10 nM among these probes (*K*_d_ of **1** and **2**: 11 and 26 nM, respectively; *K*_i_ of **3**: 33 nM). Compared to the PP derivatives Sulfo-Cy5-[K^4^]hPP, **1** and **2**, fluorescent ligand **3** exhibits a considerably lower molecular weight, which is considered favorable. However, with a *K*_i_ value of 33 nM, compound **3** exhibits only moderate Y_4_R binding affinity for application as a molecular tool.

**Figure 1:**
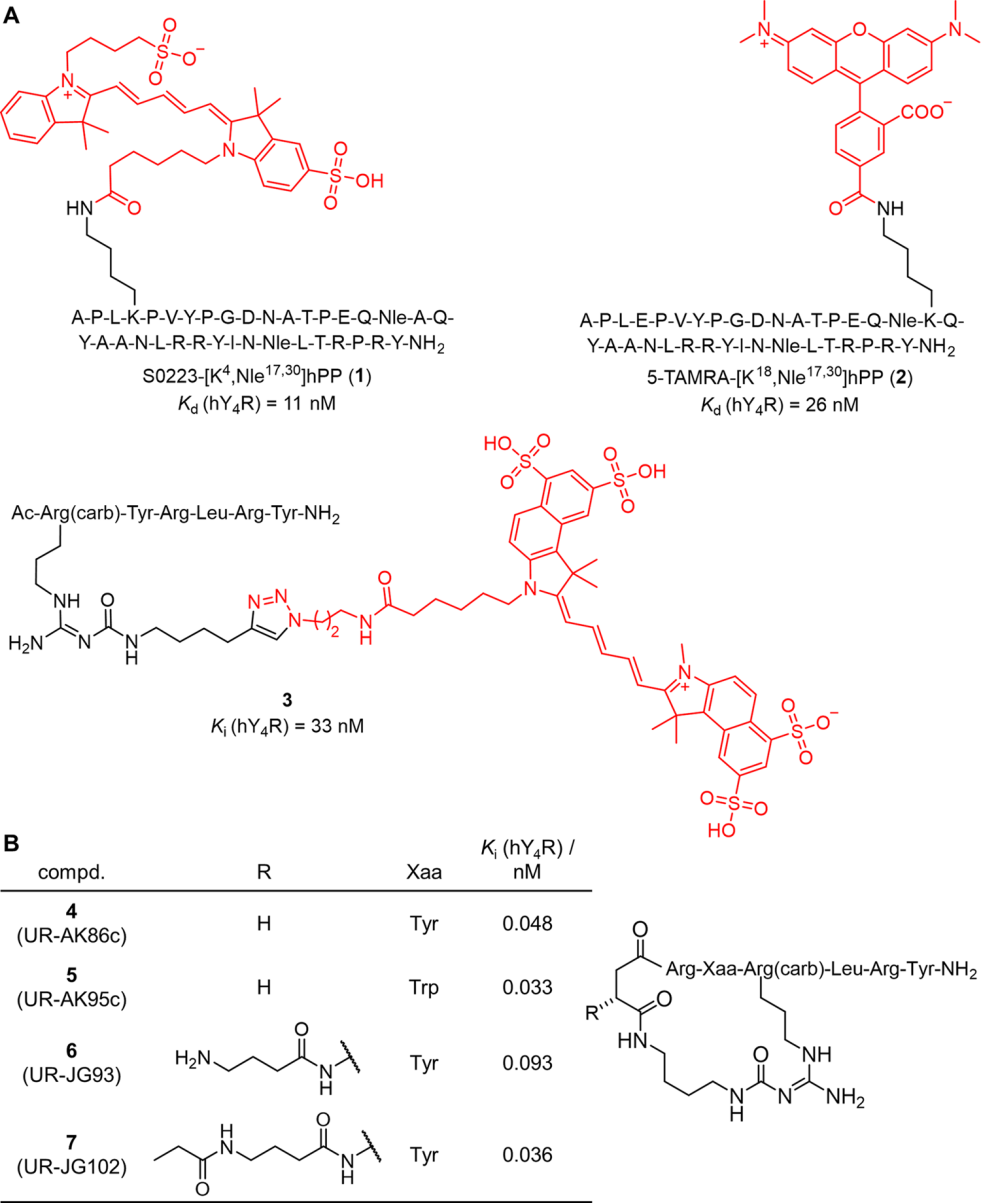
(A) Structures and Y_4_R affinities of reported Y_4_R fluorescent ligands. **1**(26) and **2**(27) represent derivatives of the endogenous Y_4_R ligand hPP labeled with sulfo-Cy5 (S0223) and 5-TAMRA, respectively. Compound **3**(29) represents a derivative of the hexapeptide UR-KK236^28^ labeled with Sulfo-Cy5.5 (lumiprobe, ref no. 7330). (B) Structures of the reported cyclic Y_4_R ligands **4**–**7** showing high binding affinity to Y_4_R (p*K*_i_ > 10).^30,31^

The present study aimed to design, synthesize, and characterize fluorescent Y_4_R ligands with low molecular weight and higher affinity to Y_4_R compared to reported probes. To achieve this, we exploited the recent discovery of the cyclic hexapeptides **4** and **5** showing two-digit picomolar Y_4_R affinities (Figure 1B).^30^ Modification of peptide **4** resulted in the amine-functionalized peptide **6** (UR-JG93), its propionylated congener UR-JG102 (**7**) (Figure 1B) and the radiolabeled analogue [^3^H]UR-JG102, all showing very high Y_4_R affinity comparable to that of **4** and **5**.^31^

The same approach was followed for the synthesis of fluorescent Y_4_R ligands, accessible by conjugation of fluorescent dyes to precursor peptide **6** (Figure 2, approach 1). Additionally, a second approach was explored by replacing Arg^1^ in peptide **4** by an alkyne-functionalized arginine enabling conjugation to a fluorescent dye by click chemistry (Figure 2, approach 2). The synthesized fluorescent ligands were studied with respect to Y_4_R binding (radiochemical competition binding assay) and Y_4_R agonistic activity (functional assays). Moreover, Y_4_R binding of selected fluorescent ligands was investigated using different luminescence-based methods (flow cytometry, fluorescence anisotropy (FA), NanoBRET assay, confocal microscopy, wide-field epifluorescence, and TIRF microscopy with single-particle tracking).

**Figure 2:**
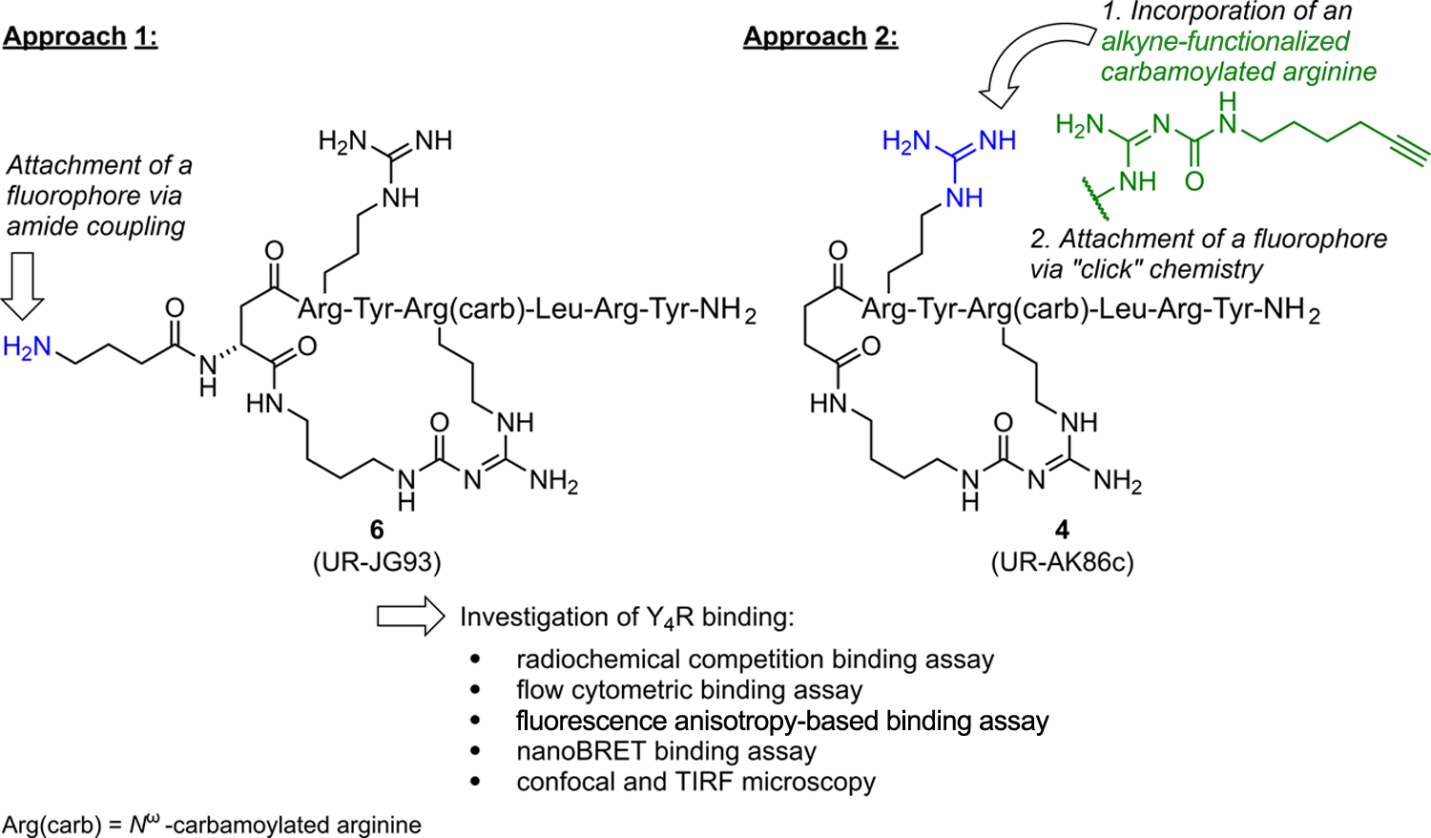
Study design of the present work: synthesis and characterization of fluorescent probes with high Y_4_R binding affinity derived from recently reported cyclic hexapeptides (**6**,^31^**4**^30^).

## Results and Discussion

### Synthesis and Chemical Stability

**Figure 3:**
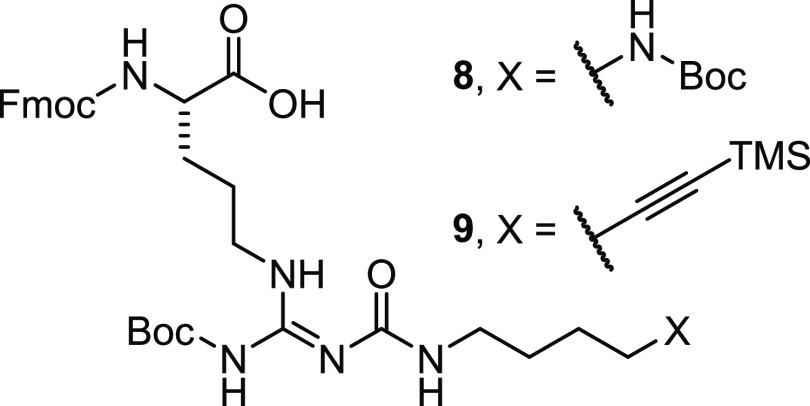
Structures of the reported *N*^ω^-carbamoylated arginines **8**(32) and **9**(29) used in SPPS for the preparation of precursor peptide **11**.

In addition to the previously described amino-functionalized peptide **6**,^31^ an alkyne-functionalized precursor peptide (**11**) was prepared for fluorescence labeling. The cyclic peptide **11** was prepared via the N-terminally succinylated linear peptide **10**, which was synthesized by solid-phase peptide synthesis (SPPS) involving the reported *N*^ω^-carbamoylated arginine building blocks **8**(32) (position 3) and **9**(29) (position 1) (structures of **8** and **9** shown in Figure 3).

**Scheme 1:**
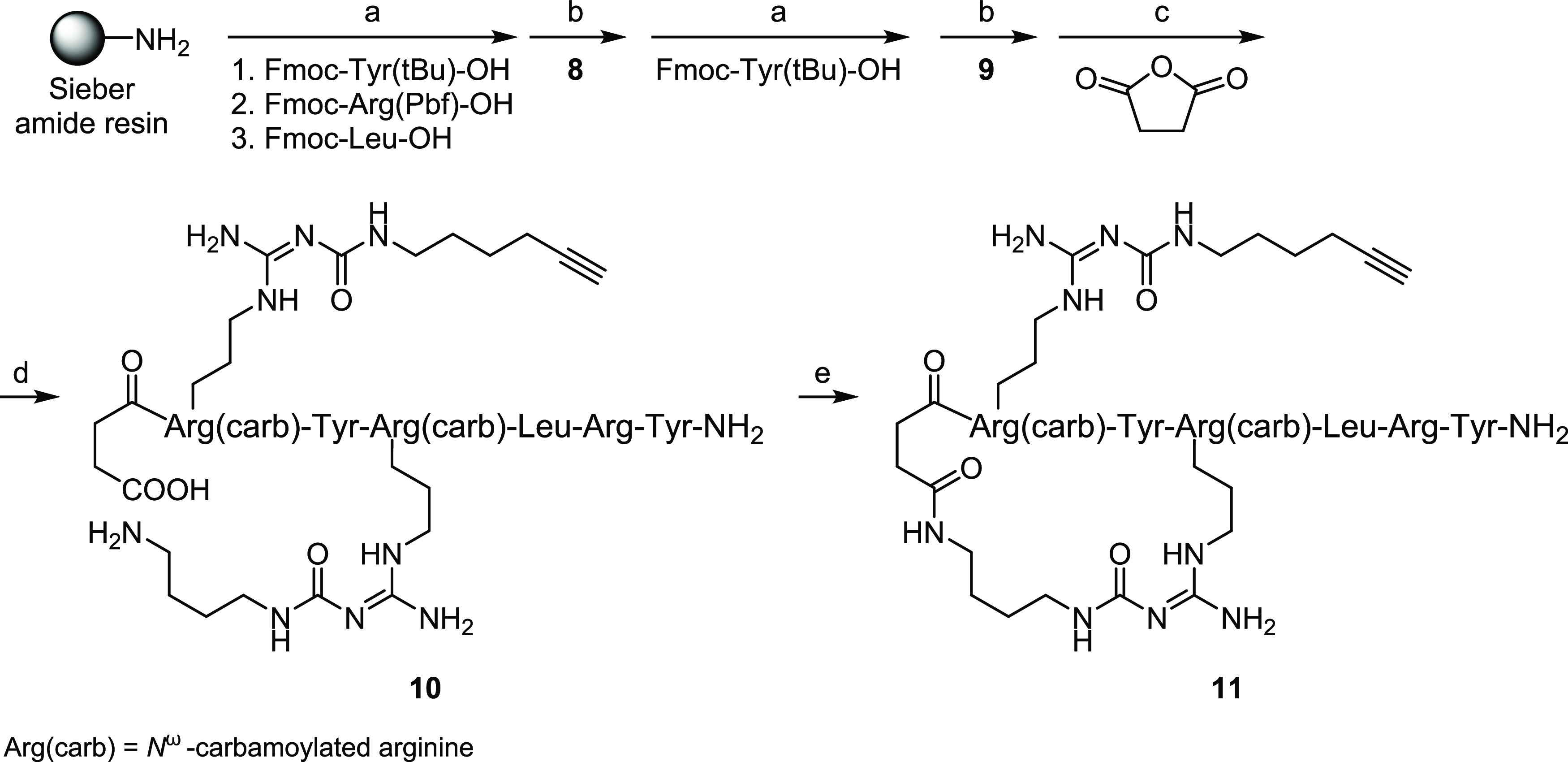
Synthesis of the Cyclic Peptide **11** via SPPS Reagents and conditions: (a) Fmoc amino acid/HOBt/HBTU/DIPEA (5/5/4.9/10 equiv), DMF/NMP 8:2 v/v, 38 °C, 2 × 45 min (“double” coupling); Fmoc deprotection (following amino acid coupling): 20% piperidine in DMF/NMP 8:2 v/v, rt, 2 × 10 min; (b) building block **8** or **9**/HOBt/HBTU/DIPEA (3/3/2.9/6 equiv), DMF/NMP 8:2 v/v, 38 °C, 16 h (single coupling); Fmoc deprotection (following amino acid coupling): as under (a); (c) succinic anhydride/DIPEA (10/10 equiv), DMF/NMP 8:2 v/v, 38 °C, 45 min; (d) (1) TFA/CH_2_Cl_2_ 1:3 v/v, rt, 2 × 20 min; (2) TFA/H_2_O 95:5 v/v, rt, 5 h; overall yield: 37%; (e) peptide cyclization: HOBt/PyBOP/DIPEA (3/2.9/6 equiv), DMF/NMP 8:2 v/v, rt, 16 h, 21%.

The amino-functionalized arginine derived from building block **8** is required for peptide cyclization, and the incorporation of **9** provides the alkyne functional group (Scheme 1). The fully deprotected peptide **10** was cyclized in solution via the succinyl carboxylic group and the primary amino group of the carbamoylated arginine in position 3 (coupling reagents: HOBt, PyBOP, DIPEA), yielding peptide **11** in 21% yield. To note, the synthesis of the alkyne-functionalized peptide **11** is more laborious compared to the preparation of the reported amine-functionalized precursor peptide **6**,^31^ as the synthesis of **11** requires two different *N*^ω^-carbamoylated arginines. The major purpose for the synthesis of peptide **11** and a fluorescent dye conjugate derived from **11** was to explore how strongly this structural modification, including the attachment of a fluorescent dye by click chemistry, affects binding to Y_4_R.

**Scheme 2:**
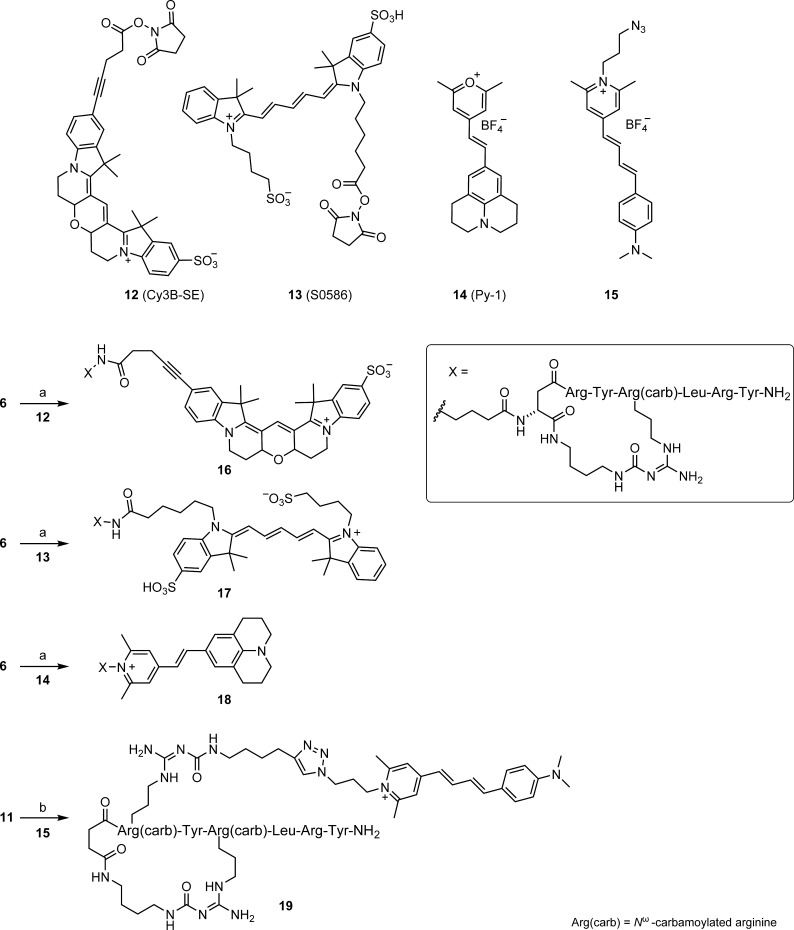
Synthesis of the Fluorescent Y_4_R Ligands **16**–**19** Reagents and conditions: (a) DIPEA, DMF, rt, 2 h, 34–69%; (b) CuSO_4_, sodium ascorbate, H_2_O/NMP 1:1 v/v, rt, 2 h, 27%.

For the preparation of the fluorescent peptides, different fluorescent dyes with various reactive groups were used (Scheme 2): the indolinium-type dyes Cy3B and Sulfo-Cy5 (S0223), used as succinimidyl esters (compounds **12** and **13**), the pyrylium dye Py-1 (**14**, yielding a pyridinium-type cyanine dye after reaction with primary amines), and the azido-functionalized pyridinium-type cyanine dye **15**, which was obtained from the pyrylium dye Py-5 and 3-azidopropane-1-amine (Scheme S1, Supporting Information). The fluorescently labeled ligands **16**–**18** were prepared from precursor **6** and the activated fluorescent dyes **12**, **13**, and **14**, respectively, using DIPEA as a base and *N*,*N*-dimethylformamide (DMF) as solvent. **16**–**18** were obtained in 34–69% yield. Fluorescent ligand **19** was synthesized from **11** and **15** via a copper(I)-catalyzed “click-reaction” in 27% yield.

The chemical stability of **16**–**18** was investigated in phosphate-buffered saline (PBS, pH 7.4) at room temperature over 48 h. The Cy3B- and Sulfo-Cy5 labeled compounds **16** and **17** showed excellent stability (Figure S1A,B, Supporting Information). The Py-1 labeled ligand **18** showed a slow decomposition (Figure S1C, Supporting Information). However, for at least up to 6 h, the degradation was marginal allowing an investigation of this fluorescent ligand in cellular assays used in this study.

### Investigation of YR Binding in Radiochemical Binding Assays

Y_1_, Y_2_, Y_4_, and Y_5_ receptor binding of **11** and **16**–**19** was determined using reported whole-cell radioligand competition binding assays (Y_1_: Keller et al.,^33^ Y_2_: Konieczny et al.,^34^ Y_4_: Konieczny et al.,^30^ Y_5_: Kuhn et al.^35^). With p*K*_i_ values >9, all studied compounds showed high Y_4_R affinities (Table 1, competition binding curves shown in Figure S2, Supporting Information). Since the *K*_i_ values were at least 500-fold lower compared to the *K*_i_ determined at Y_1_, Y_2_, and Y_5_ receptors, **11** and **16**–**19** also exhibit pronounced Y_4_R selectivity.

**Table 1:** Y Receptor Binding Data of hPP, **4**–**6**, **11**, and **16**–**19**

	p*K*_i_ ± SEM/*K*_i_ [nM]			
compd.	hY_1_R^a^	hY_2_R^b^	hY_4_R^c^	hY_5_R^d^
hPP	6.4/440^e^	<5.5/>3000^e^	10.02 ± 0.06/0.10^f^	7.8/17^e^
**4**	<5.5/>3000^g^	<5/>10000^g^	10.36 ± 0.11/0.048^g^	<5.5/>3000^g^
**5**	<5/>10000^g^	<5/>10000^g^	10.48 ± 0.04/0.033^g^	<5.5/>3000^g^
**6**	<6/>1000^h^	6.04 ± 0.07/950^h^	10.04 ± 0.04/0.093^h^	<6/>1000^h^
**11**	<5/>10000	<5/>10000	9.38 ± 0.04/0.42	<5.5/>3000
**16**	6.91 ± 0.01/120	<6/>1000	9.71 ± 0.09/0.21	6.32 ± 0.04/480
**17**	<6/>1000	<6/>1000	9.48 ± 0.09/0.35	<6/>1000
**18**	<6/>1000	<6/>1000	9.22 ± 0.03/0.61	6.08 ± 0.05/840
**19**	<6/>1000	<6/>1000	9.22 ± 0.06/0.62	<6/>1000

a Determined by competition binding at SK-N-MC neuroblastoma cells using [^3^H]UR-MK299 (*K*_d_ = 0.054 nM, *c* = 0.15 nM) as radioligand.

b Determined by competition binding with [^3^H]propionyl-pNPY (*K*_d_ = 0.14 nM,^34^*c* = 0.5 nM) at CHO-hY_2_R cells.

c Determined by competition binding at CHO-hY_4_R-G_qi5_-mtAEQ cells using [^3^H]UR-KK200 (*K*_d_ = 0.67 nM,^35^*c* = 1 nM) as radioligand.

d Determined by competition binding at HEC-1B-hY_5_R cells using [^3^H]propionyl-pNPY (*K*_d_ = 11 nM,^26^*c* = 5 nM) as radioligand.

e Berlicki et al. (reported *K*_i_ values were converted to p*K*_i_ values).^36^

f Wirth et al.^37^

g Konieczny et al.^30^

h Gleixner et al.^31^ Data represent mean values ± SEM (p*K*_i_) or mean values (*K*_i_) from 3 to 4 independent experiments performed in triplicate.

Consequently, incorporation of the alkyne-functionalized carbamoylated arginine instead of Arg^1^ in the parent compound **4** (compound **11**) and coupling to a fluorescent dye (**19**) only slightly affects Y_4_R binding (Table 1). Likewise, the conjugation of fluorescent dyes to precursor peptide **6** did not alter Y_4_R binding, underlining the addressed position in **6** as favorable for the attachment of bulky moieties.

### Functional Studies at Y_4_R

Y_4_R agonistic activities of **11** and **16**–**19** were investigated in a Ca^2+^ aequorin assay,^25,34^ measuring the increase in cytosolic Ca^2+^, and a miniG_si_ recruitment assay,^37^ detecting binding of an artificial G-protein to the Y_4_R. Additionally, compounds **16** and **17** were studied in a cAMP CAMYEN assay^31^ to measure changes in cytosolic cAMP levels. The CAMYEN assay uses a Nanoluciferase as the BRET donor in place of the “Renilla” luciferase found in the traditional CAMYEL BRET assay, while retaining the rest of the sensor. Due to the increased brightness of the Nanoluciferase compared to the “Renilla” luciferase, the CAMYEN biosensor has an increased bioluminescence signal readout, as well as a bigger window in the signal-to-baseline of the cAMP responses, reducing saturation of the sensor. This allows for easier and faster detection of CAMYEN sensor activation by plate readers and a greater potential for determining ligand efficacy (i.e., partial agonism) when compared to the CAMYEL assay.

To note, control experiments with hPP in the absence and presence of dummy fluorescent ligands showed that the readout of the aequorin (Ca^2+^-assay) and Nanoluciferase (Nluc, miniG_si_ assay) luminescence is only slightly affected by the fluorophore at high fluorescent ligand concentrations (Figure S6, Supporting Information).

In the Ca^2+^ and mini G_si_ assays, the studied peptides displayed partial agonism with efficacies ranging from 56 to 71 and 43 to 72%, respectively (Table 2, concentration–response curves shown in Figures S3 and S4, Supporting Information).

**Table 2:** Y_4_R Agonistic Activities of hPP, **4**–**6**, **11**, and **16**–**19**^a^

	Ca^2+^-aequorin		miniG_si_ recruitment		CAMYEN cAMP	
cmpd.	pEC_50_ ± SEM/EC_50_ [nM]	*E*_max_ ± SEM/%	pEC_50_ ± SEM/EC_50_ [nM]	*E*_max_ ± SEM/%	pEC_50_ ± SEM/EC_50_ [nM]	*E*_max_ ± SEM/%
hPP	7.90 ± 0.2/17^b^	100^b^	8.94 ± 0.01/1.1^b^	100^b^	9.85 ± 0.09/0.15^b^	100^b^
**4**	8.57 ± 0.03/2.7^c^	81 ± 1^c^	n.d.	n.d.	9.79 ± 0.1/0.17^b^	113 ± 9^b^
**5**	9.00 ± 0.07/0.99^c^	84 ± 2^c^	8.74 ± 0.04/1.8	61 ± 2	n.d.	n.d.
**6**	7.76 ± 0.05/18^b^	69 ± 3^b^	8.60 ± 0.03/2.5^b^	62 ± 2^b^	n.d.	n.d.
**11**	7.01 ± 0.01/98	56 ± 3	7.2 ± 0.1/73	62 ± 1	n.d.	n.d.
**16**	7.8 ± 0.1/19	66 ± 2	7.4 ± 0.1/41	72 ± 9	9.58 ± 0.06/0.26	93 ± 5
**17**	7.41 ± 0.05/40	71 ± 3	7.07 ± 0.09/93	70 ± 9	9.65 ± 0.05/0.22	90 ± 3
**18**	7.3 ± 0.1/65	61 ± 5	8.15 ± 0.01/7.1	44 ± 2	n.d.	n.d.
**19**	7.25 ± 0.07/58	64 ± 2	7.38 ± 0.02/42	43 ± 1	n.d.	n.d.

a Agonistic potencies (pEC_50_, EC_50_) and efficacies *E*_max_ (relative to the maximum effect elicited by 1 μM hPP, *E*_max_ = 100%) determined in a Ca^2+^-aequorin assay (CHO-hY_4_R-G_qi5_-mtAEQ cells), a miniG_si_ recruitment assay (HEK293T-NlucN-mG_si_/Y_4_R-NlucC cells) and in a CAMYEN cAMP assay (HEK293T-CAMYEN-hY_4_R cells).

b Gleixner et al.^31^

c Konieczny et al.^30^ Data represent mean values ± SEM (pEC_50_, *E*_max_) or mean values (EC_50_) from three or four independent experiments performed in triplicate. n.d. = not determined.

As previously observed for peptidic Y_4_R agonists,^31^ the potencies (pEC_50_) obtained from the Ca^2+^ and miniG_si_ assay were consistently lower than the respective binding affinities (p*K*_i_ values; Tables 1 and 2), which can be attributed to the nonequilibrium conditions in the case of the functional assays. In contrast, the pEC_50_ values of **16** and **17**, determined in the cAMP CAMYEN assay, were in good agreement with the p*K*_i_ values from the radiochemical competition binding assay, which is explainable by a downstream signal amplification for this type of functional assay.

### Fluorescence Characterization

The excitation and emission spectra of **16**–**19** and the emission quantum yield were recorded in PBS and PBS supplemented with 1% bovine serum albumin (BSA, spectra shown in Figure S7, Supporting Information). The Cy3B-labeled ligand **16** showed the highest quantum yield with 67–69% (Table 3). The small spectral shifts of the maxima of the excitation and emission spectra in the presence of BSA may indicate some unspecific interactions to protein surfaces. However, as the fluorescence quantum yield is not altered in the presence of BSA, these interactions have no significant impact on the general photophysics of the Cy3B dye. Interestingly, comparison to the Cy3B derivative, which was obtained by preparation of an amide from succinimidyl ester **12** (Cy3B-SE, cf. Scheme 2) and 2-aminoethanol,^38^ reveals that the peptide moiety in fluorescent ligand **16** mediates an increase in fluorescence quantum yield by ca. 10%.

**Table 3:** Maxima of the Excitation and Emission Spectra and Fluorescence Quantum Yields of **16**–**19**, Determined in PBS (pH = 7.4) and PBS Containing 1% BSA

		λ_ex_ [nm]/λ_em_ [nm]/Δ [eV]		Φ [%]	
compd.	dye	PBS	PBS with 1% BSA	PBS	PBS with 1% BSA
**16**	Cy3B	571/584/0.048	572/586/0.052	69	67
**17**	Sulfo-Cy5	648/665/0.049	657/670/0.037	26	33
**18**	py-1	517/633/0.439	506/595/0.367	2	36
**19**	py-5	496/692/0.708	515/644/0.482	4	24

For the Sulfo-Cy5 labeled compound **17**, the bathochromic shift is more pronounced and a small increase in fluorescence quantum yield is observed in the presence of BSA, which was also previously observed for peptidic neurotensin receptor ligands.^39^ In the case of the pyridinium-type cyanine dye-labeled ligands **18** and **19**, a hypsochromic shift of the first electronic absorption and a considerably higher fluorescence quantum yield in the presence of BSA is observed (Table 3). This phenomenon, also reported for other Py-1- and Py-5-labeled receptor ligands,^40−42^ can be explained by strongly reduced degrees of freedom in configurational space of the fluorophore due to binding interactions with amino acid residues on the BSA surface, which is evidenced by the marked reduction in the Stokes shift observed for **18** and **19**. In contrast to the Cy3B dye in **16**, the conformationally less restricted dyes in **17**–**19** show a Stokes shift reduction and enhancement of the fluorescence quantum yield in the presence of BSA. It is tempting that in the dyes with higher flexibility (as in **17**–**19**) the excitation energy is rapidly released via pronounced geometric changes favoring internal conversion rather than fluorescence and that rigidification due to binding to BSA blocks these deactivation channels making fluorescence a competing process. Currently, detailed mechanistic studies are conducted in our laboratories to elucidate the excited state deactivation of the pyridinium-type cyanine dyes in **18** to **19** on a molecular level.

### Flow Cytometric Y_4_R Binding Studies

**Figure 4:**
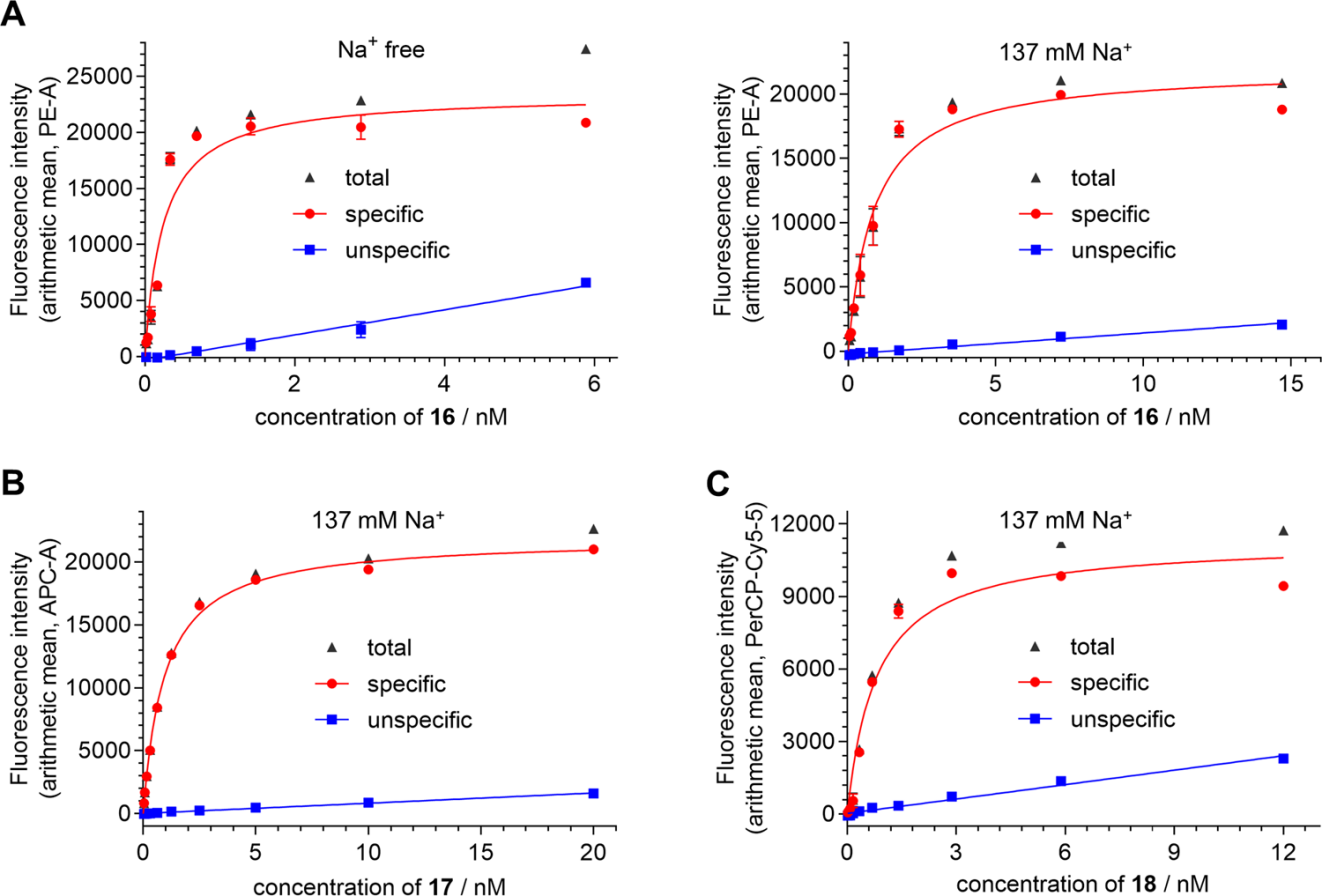
Flow cytometric saturation binding of **16** (A), **17** (B), and **18** (C) at whole CHO-hY_4_R-G_qi5_-mtAEQ cells at 22 ± 2 °C. (A) Representative saturation isotherms (red circle) of **16** from experiments performed in sodium-free buffer (buffer I) and sodium-containing buffer (DPBS, 137 mM Na^+^). (B, C) Representative saturation isotherms (red circle) of **17** (B) and **18** (C) from experiments performed in sodium-containing buffer (DPBS). Unspecific binding (blue squares) was determined in the presence of 1 μM hPP (A–C). Total and unspecific binding data represent mean values ± SEM. Specific binding, representing calculated values ± propagated error, were fitted according to an equation describing a hyperbolic isotherm (binding-saturation: one site-specific binding, GraphPad Prism 5).

Y_4_R binding of **16**–**18** was investigated in flow cytometry-based binding experiments using CHO-hY_4_R-G_qi5_-mtAEQ cells,^25^ which were also used for radiochemical binding assays and the Ca^2+^ aequorin assay. Saturation binding with the Cy3B-labeled ligand **16** was performed in a sodium-free buffer (buffer I, composition see the Experimental Section) and in sodium-containing (137 mM Na^+^) DPBS (Figure 4A). This comparison was of interest as previous studies revealed marked differences in Y_4_R affinities of Y_4_R (partial) agonists depending on the presence or absence of sodium in the binding buffer (up to 20-fold lower affinity in the presence of Na^+^ at a physiological concentration).^26,31,35^ As receptor binding studies should generally be performed under physiological-like conditions, i.e., in sodium-containing buffers, **17** and **18** were only studied in DPBS. The flow cytometric investigation of the cell viability by propidium-based live/dead staining revealed that in sodium-free buffer (buffer I) the major fraction of CHO-hY_4_R-G_qi5_-mtAEQ cells was nonviable already after 15 min of incubation at 22 °C (Figure S8A, Supporting Information). Over time (followed up to 6 h), the fraction of intact cells further decreased. In contrast, CHO-hY_4_R-G_qi5_-mtAEQ cells suspended in DPBS were largely intact even after 6 h of incubation (Figure S8B, Supporting Information). As the dissociation of **16** from CHO-hY_4_R-G_qi5_-mtAEQ cells was followed over 6 h in buffer I (see below), a data analysis based on the intact cell population was not reasonable for the whole experiment. Therefore, for all flow cytometric binding experiments performed with **16** in buffer I (saturation binding, association and dissociation kinetics, competition binding), data analysis was performed based on the nonviable cell population (corresponds to population P2 in Figure S8A, Supporting Information).

Saturation binding studies with fluorescent ligand **16** gave a slightly lower *K*_d_ value for buffer I (*K*_d_: 0.30 nM) compared to DPBS (*K*_d_: 0.70 nM). Notably, analysis of the saturation binding data based on the intact cell population (performed as a control), yielded the same *K*_d_ values as obtained from the analysis based on the nonviable cell population, showing that the loss of cell integrity had no or only little impact on Y_4_R binding of **16** (cf. Figure S9, Supporting Information). With *K*_d_ values <1 nM, fluorescent ligand **16** showed higher Y_4_R binding affinity compared to previously reported fluorescently labeled Y_4_R ligands.^25−27,29^ Saturation binding with **17** and **18** yielded *K*_d_ values of 0.93 and 0.51 nM, respectively (Table 4 and Figure 4B,C) indicating that the type of fluorophore has almost no effect on Y_4_R binding.

**Table 4:** Parameters Characterizing Y_4_R Binding of **16**–**18** Determined in Flow Cytometric Binding Assays at 22 ± 2 °C Using CHO-hY_4_R-G_qi5_-mtAEQ Cells

		saturation binding	binding kinetics					
cmpd.	buffer	p*K*_d_/*K*_d_ [nM]^a^	*k*_obs(mono)_ [min^–1^]^b^	*k*_obs(bi,fast)_ [min^–1^]^c^*k*_obs(bi,slow)_ [min^–1^]^c^	*k*_off_ [min^–1^]^d^	*k*_on(mono)_ [nM^–1^ min^–1^]^e^	*k*_on(bi,fast)_ [nM^–1^ min^–1^]^e^ k_on(bi,slow)_ [nM^–1^ min^–1^]^e^	*K*_d_(kin) [nM]^f^
**16**	buffer I (Na^+^-free)	9.56 ± 0.09/0.30	0.13 ± 0.02	n.a.	0.0069 ± 0.0006	0.41 ± 0.07	n.a.	0.017 ± 0.004
**16**	DPBS (137 mM Na^+^)	9.16 ± 0.05/0.70	0.18 ± 0.04	1.0 ± 0.2	0.012 ± 0.001	0.24 ± 0.05	1.3 ± 0.3	with *k*_on(mono)_: 0.05 ± 0.02
				0.048 ± 0.003			0.050 ± 0.006	with *k*_on(bi,fast)_: 0.009 ± 0.002
								with *k*_on(bi,slow)_: 0.25 ± 0.05
**17**	DPBS (137 mM Na^+^)	9.03 ± 0.02/0.93	0.15 ± 0.01	1.89 ± 0.05	0.026 ± 0.002	0.12 ± 0.01	1.87 ± 0.05	with *k*_on(mono)_: 0.21 ± 0.03
				0.071 ± 0.008			0.045 ± 0.009	with *k*_on(bi,fast)_: 0.007 ± 0.001
								with *k*_on(bi,slow)_: 0.29 ± 0.07
**18**	DPBS (137 mM Na^+^)	9.3 ± 0.1/0.51	0.10 ± 0.02	0.7 ± 0.1	0.010 ± 0.001	0.18 ± 0.04	1.3 ± 0.2	with *k*_on(mono)_: 0.06 ± 0.02
				0.034 ± 0.001			0.048 ± 0.004	with *k*_on(bi,fast)_: 0.008 ± 0.002
								with *k*_on(bi,slow)_: 0.20 ± 0.05

a Equilibrium dissociation constant expressed as p*K*_d_ (mean values ± SEM) and *K*_d_ (mean values) obtained from at least three independent experiments (performed in triplicate).

b Observed association rate constant obtained by monophasic fitting (exponential rise to a maximum); mean values ± SEM from three independent experiments (performed in duplicate).

c Observed association rate constant obtained by biphasic fitting; mean values ± SEM from three or four independent experiments (performed in duplicate).

d Dissociation rate constant obtained from three-parameter monophasic fits (exponential decline); mean values ± SEM from three or four independent experiments (performed in duplicate).

e Association rate constant ± propagated error calculated from *k*_obs(mono)_, *k*_obs(bi,fast)_, or *k*_obs(bi,slow)_ values, the respective *k*_off_ value, and the ligand concentration used for the association experiments.

f Kinetically derived dissociation constant ± propagated error calculated from *k*_off_ and *k*_on_ values. n.a.: not applicable

**Figure 5:**
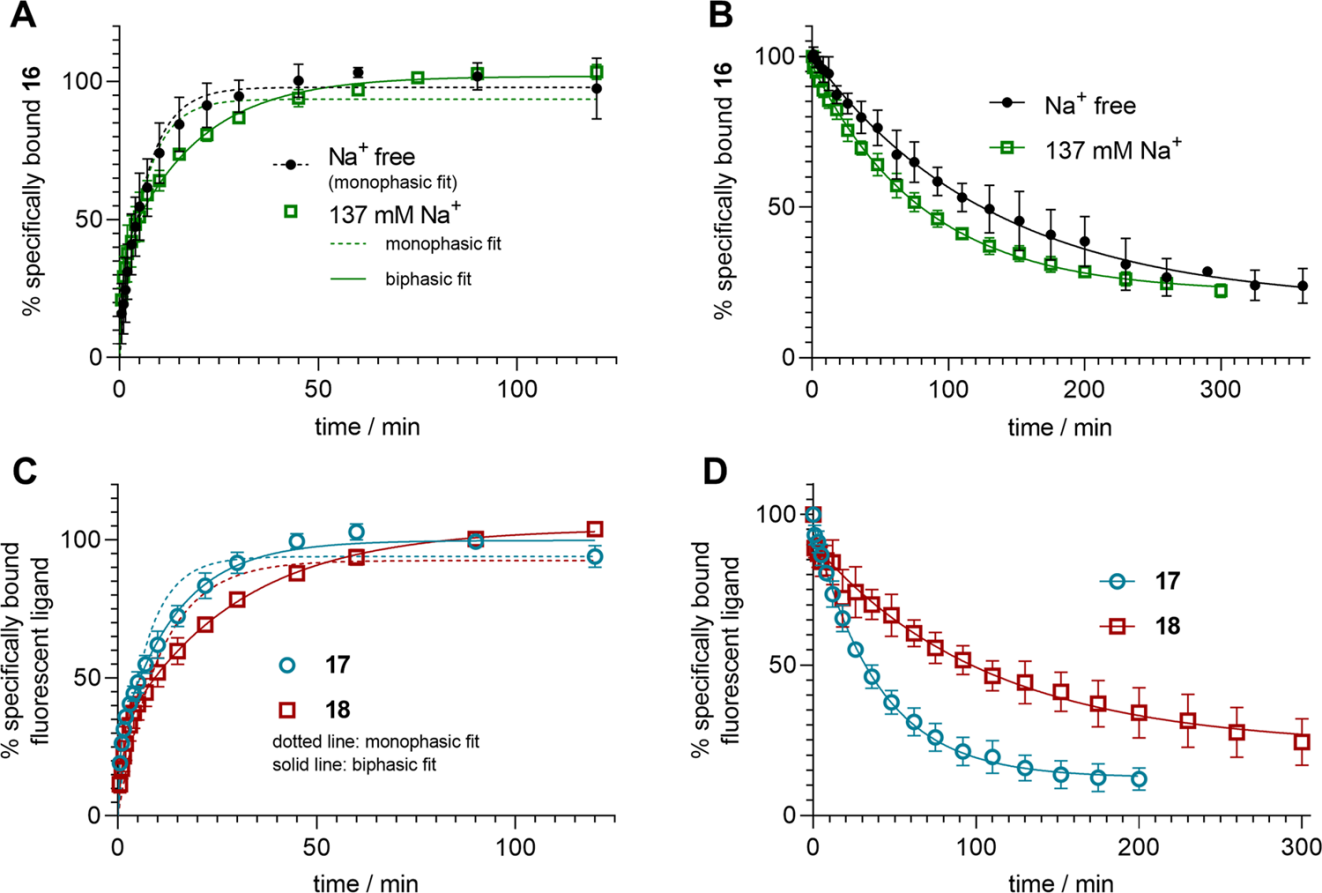
Binding kinetics of **16**–**18** determined by flow cytometry at whole CHO-hY_4_R-G_qi5_-mtAEQ cells at 22 ± 2 °C. (A) Association of **16** to the hY_4_R under sodium-free conditions (buffer I) and in sodium-containing buffer (DPBS, 137 mM Na^+^). Concentration of **16**: 0.3 and 0.7 nM, respectively. Proportion of the initial fast association (two-phase association fit, GraphPad Prism 5): 38 ± 4% (mean ± SEM). (B) Dissociation of **16** from the hY_4_R determined in buffer I and DPBS. The dissociation was initiated after 1.5 h of preincubation with **16** (*c* = 1.5 nM (Na^+^-free) and 3.5 nM (137 mM Na^+^)) by the addition of an excess of hPP (1000-fold) and **5** (100-fold). Plateau values of the three-parameter fits (monophasic exponential decline): 13% (Na^+^-free), 22% (137 mM Na^+^). (C) Association of **17** (*c* = 1 nM) and **18** (*c* = 0.5 nM) to the hY_4_R determined in DPBS (137 mM Na^+^). Proportion of the initial fast association (two-phase association fit, GraphPad Prism 5): 74 ± 1% (**17**), 29 ± 3% (**18**) (mean values ± SEM). (D) Dissociation of **17** and **18** from the hY_4_R in DPBS. The dissociation was initiated after 1.5 h of preincubation with **17** (*c* = 5 nM) or **18** (*c* = 2.5 nM) by the addition of an excess of hPP (1000-fold) and **5** (100-fold). Plateau values of the three-parameter fits (monophasic exponential decline): 13% (**17**), 22% (**18**). Data (A–D) represent mean values ± SEM from three independent experiments performed in duplicate.

All data obtained from association binding experiments in DPBS correspond better to a biphasic than to a monophasic curve (Figure 5A,C), as supported by a statistical extra sum-of-squares *F*-test (one-phase association vs two-phase association, GraphPad Prism 5) giving *P* values <0.002. In contrast, the association of **16** in sodium-free buffer was monophasic (*F*-test derived *P*-value: 0.55) (Figure 5A). For all studied fluorescent ligands (**16**–**18**), association experiments indicated that equilibrium is reached after approximately 1 h (Figure 5A,C). Worth mentioning, the structurally related radioligand [^3^H]UR-JG102 ([^3^H]**7**), also studied at CHO-hY_4_R-G_qi5_-mtAEQ cells in sodium-free (buffer I) and sodium-containing (DPBS) buffer, showed similar kinetics being monophasic in buffer I and biphasic in DPBS. However, compared to **16**–**18**, the biphasic character of the association curve of [^3^H]**7** in DPBS was considerably more pronounced.^31^ As described for [^3^H]**7**, the first (fast) association phase could represent association to a subpopulation of receptors that are sodium-bound.^31^ It is well known that sodium stabilizes the inactive receptor conformation of GPCRs,^43^ presumably being better accessible for receptor agonists due to a more open passage to the ligand binding pocket.^43,44^ The second (slow) association could represent binding to a population of nonsodium-bound receptors (note: at 150 mM sodium a GPCR population is not necessarily saturated with sodium^44^), which are prone to adapt the active receptor conformation. At first sight, this disagrees with the observed proportions of the initial (fast) and the second (slow) association phase, which varied considerably for the fluorescent ligands **16**–**18** (values given in the caption of Figure 5). However, as the receptor conformation also depends on the coupling to the G-protein and as G-protein binding may influence ligand binding,^45^ the inconsistency of the proportions could be explained by a ligand bias with respect to the G-protein coupling efficiency of the agonist-bound receptor and in terms of G-protein modulated ligand affinity. This is in accordance with the monophasic association of **16** observed in the FA assay using Y_4_R displaying BBVs instead of whole cells (see below). It should also be kept in mind that during the association process, dissociation takes place, which codetermines the result of an association experiment. Furthermore, provided that the receptor populations are interconvertible and that equilibria among different receptor conformations can be modulated by the ligand,^46^ a readjustment of the equilibrium between distinct receptor populations may occur upon ligand binding, which can be differently pronounced for different ligands.

The dissociation of **16** was monophasic for the sodium-free and sodium-containing buffer (Figure 5B). As also observed for the radioligand [^3^H]**7**, the dissociation of **16** from Y_4_R was incomplete (plateau significantly higher than zero, *P* < 0.05, *t* test). The minor component of long-lived Y_4_R binding may be explained by a conformational readjustment of the receptor protein upon ligand binding^47^ or by an increased rebinding capability due to simultaneous interaction with more than one binding site.^48^ Moreover, as **16** is a Y_4_R partial agonist, the incomplete dissociation could also be caused by ligand-induced receptor internalization. The Sulfo-Cy5 labeled compound **17** and the Py-1 labeled probe **18** showed a monophasic dissociation comparable to that of **16** in terms of *k*_off_ values and the minor component of long-lived Y_4_R binding (Figure 5D and Table 4).

The kinetically derived dissociation constants *K*_d(kin)_ of **16**–**18** obtained in DPBS were calculated from *k*_off_ and *k*_on(bi,fast)_, *k*_off_, and *k*_on(bi,slow)_, and additionally from *k*_off_ and *k*_on(mono)_, as the latter represents the whole association process and also with regard to the fact that the biphasic character of the association was only weakly pronounced. The *K*_d(kin)_ values were consistently lower than the equilibrium *K*_d_ values obtained from saturation binding experiments. For all three fluorescent ligands, the lowest discrepancy between *K*_d(kin)_ and the equilibrium *K*_d_ (factor 2.6–3.2) is obtained when *k*_on(bi,slow)_ is used for the calculation (Table 4). This indicates that Y_4_R binding of **16**–**18** in DPBS largely proceeds according to the law of mass action for an incubation time of approximately 1 h. Flow cytometric Y_4_R competition binding studies in buffer I and DPBS using **16** as labeled ligand are discussed below.

### Fluorescence Anisotropy-Based Y_4_R Binding Studies

As Cy3B exhibits a fluorescence lifetime (τ(PBS) = 2.27 ns, τ(PBS + 1% BSA) = 2.74 ns^38^) compatible with FA measurements, the Cy3B-labeled fluorescent ligand **16** was investigated in FA-based Y_4_R binding assays. For these experiments, Y_4_R displaying budded baculovirus particles (BBVs) were used. With a uniform size of approximately 300 nm × 50 nm (length × diameter), BBVs are well suited for FA measurements.^19^ To note, techniques that are not based on the use of polarized light (e.g., radiochemical, flow cytometric, and NanoBRET binding assays), require a large excess of the labeled probe relative to the amount of receptor used. In contrast, FA measurements require approximately equal concentrations of the labeled ligand and receptor. Accordingly, ligand depletion must be taken into account for FA data analysis.

For Y_4_R binding studies, two batches of Y_4_R displaying BBVs were prepared in SF9 insect cells: the Y_4_R was either expressed alone or in combination with enzymes catalyzing a mammalian-like receptor glycosylation (so-called “SweetBac” system^49^). In the present article, the term Y_4_R^nonglyco^ is used for the former approach, precluding a mammalian-like glycosylation of Y_4_R, and the term Y_4_R^SwBac^ is used for the latter (we do not use the term Y_4_R^glyco^ as glycosylation was not proven).

**Figure 6:**
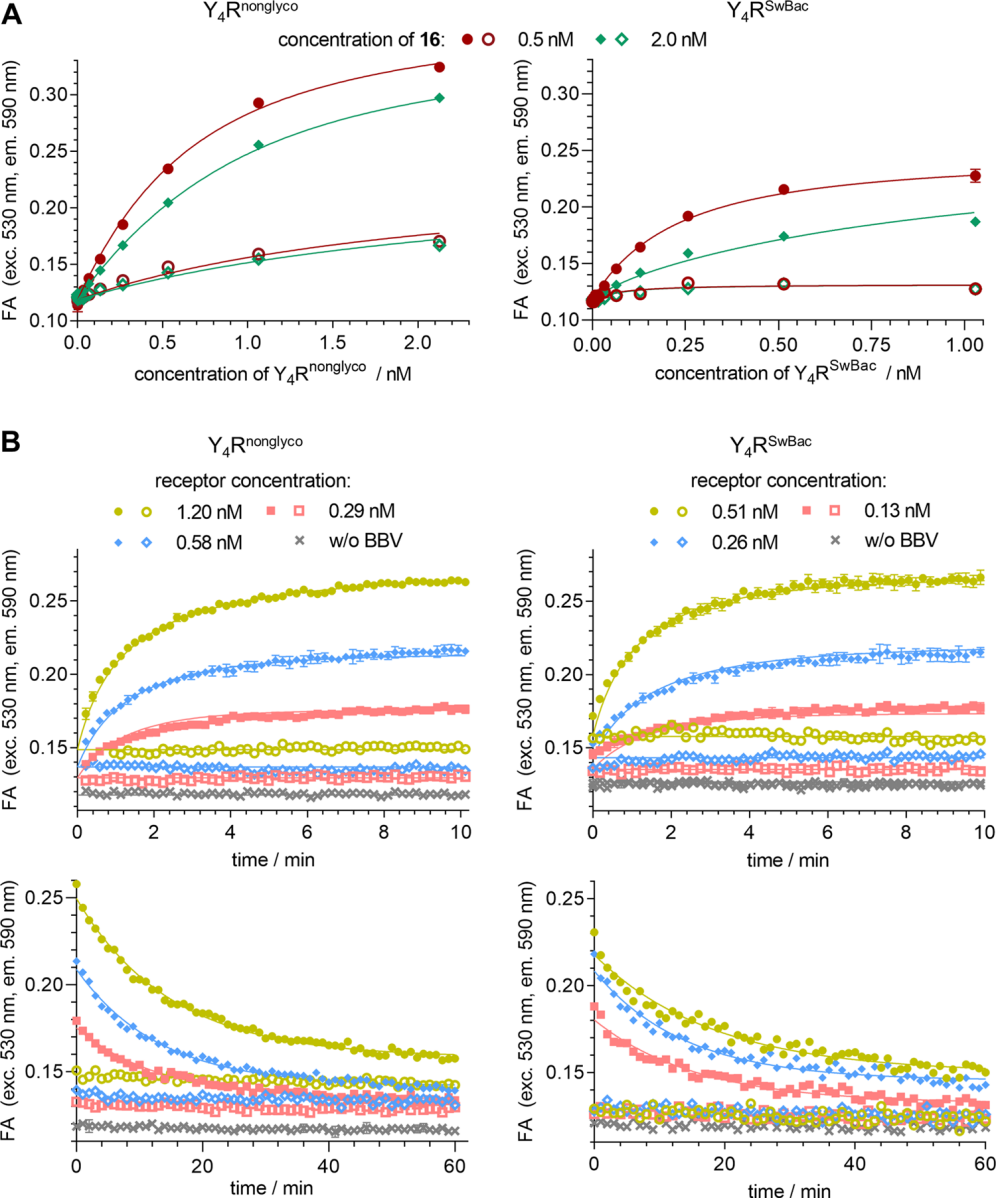
Binding of **16** to hY_4_R displaying BBVs studied by FA measurement at 27 °C. (A) Binding isotherms of **16** obtained from experiments using fixed concentrations of **16** (0.5 or 2 nM) and increasing amounts of Y_4_R. Total binding is represented by filled symbols and unspecific binding (determined in the presence of 1 μM hPP) is represented by open symbols. Depicted data (mean values ± SEM from a representative experiment performed in duplicate) represent snapshots at 90 min incubation. Y_4_R concentrations displayed on the abscissa were calculated after global analysis of the data from three or four individual experiments by a modified version of a model described by Veiksina et al.,^19^ affording the estimated binding site (Y_4_R) concentration of the applied BBV stock which amounted to 6 ± 1 nM (Y_4_R^nonglyco^, mean value ± SEM, *n* = 3) or 2.1 ± 0.1 nM (Y_4_R^SwBac^, mean value ± SEM, *n* = 4). (B) Association and dissociation of **16** (0.5 nM) determined in real time for three different Y_4_R concentrations (green, blue, and red symbols). Total binding is represented by filled symbols and unspecific binding (determined in the presence of 1 μM hPP) is represented by open symbols. Data represent mean values ± SEM from a representative experiment performed in duplicate.

All FA binding experiments with **16** were performed in DPBS (137 mM Na^+^). Equilibrium binding involving two different concentrations of **16** yielded *K*_d_ values of 0.60 nM (Y_4_R^nonglyco^) and 0.14 nM (Y_4_R^SwBac^) (Figure 6A and Table 5), essentially being in agreement with the equilibrium *K*_d_ value of **16** obtained from flow cytometric saturation binding studies in DPBS (0.70 nM). These experiments also provide information about the Y_4_R concentration of the BBV stocks (plotted at the abscissa in Figure 6A, values provided in the figure caption).

**Table 5:** Parameters Characterizing Y_4_R Binding of **16** Determined in Fluorescence Anisotropy-Based Binding Assays at 27 °C Using Y_4_R^nonglyco^ and Y_4_R^SwBac^ Displaying BBVs

	saturation binding	binding kinetics		
BBV	p*K*_d_/*K*_d_ [nM]^a^	*k*_on_ [nM^–1^ min^–1^]^b^	*k*_off_ [min^–1^]^c^	*K*_d(kin)_ [nM]^d^
Y_4_R^nonglyco^	9.2 ± 0.1/0.60	0.015 ± 0.004	0.0012 ± 0.0001	0.11 ± 0.03
Y_4_R^SwBac^	9.9 ± 0.1/0.14	0.021 ± 0.002	0.00098 ± 0.00003	0.052 ± 0.006

a Equilibrium dissociation constant expressed as p*K*_d_ (mean values ± SEM) and *K*_d_ (mean value) obtained from three independent experiments (performed in duplicate).

b Association rate constant ± SEM obtained from global analysis^19^ of data from three individual experiments (performed in duplicate) each involving two different concentrations of **16** (0.5 and 7.0, or 0.5 and 2.0 nM).

c Dissociation rate constants obtained from three-parameter monophasic fits (exponential decline). Mean values ± SEM from three independent experiments (performed in triplicate).

d Kinetically derived dissociation constants ± SEM calculated from the mean *k*_off_ value and individual *k*_on_ values.

Association and dissociation experiments with **16** were performed with six different receptor concentrations and 2–3 different fluorescent ligand concentrations. The association and dissociation curves obtained for a ligand concentration of 0.5 nM are shown for three selected receptor concentrations in Figure 6B. For both Y_4_R batches, the association and dissociation curves were monophasic and the dissociation was nearly complete with plateau values <12%.

**Figure 7:**
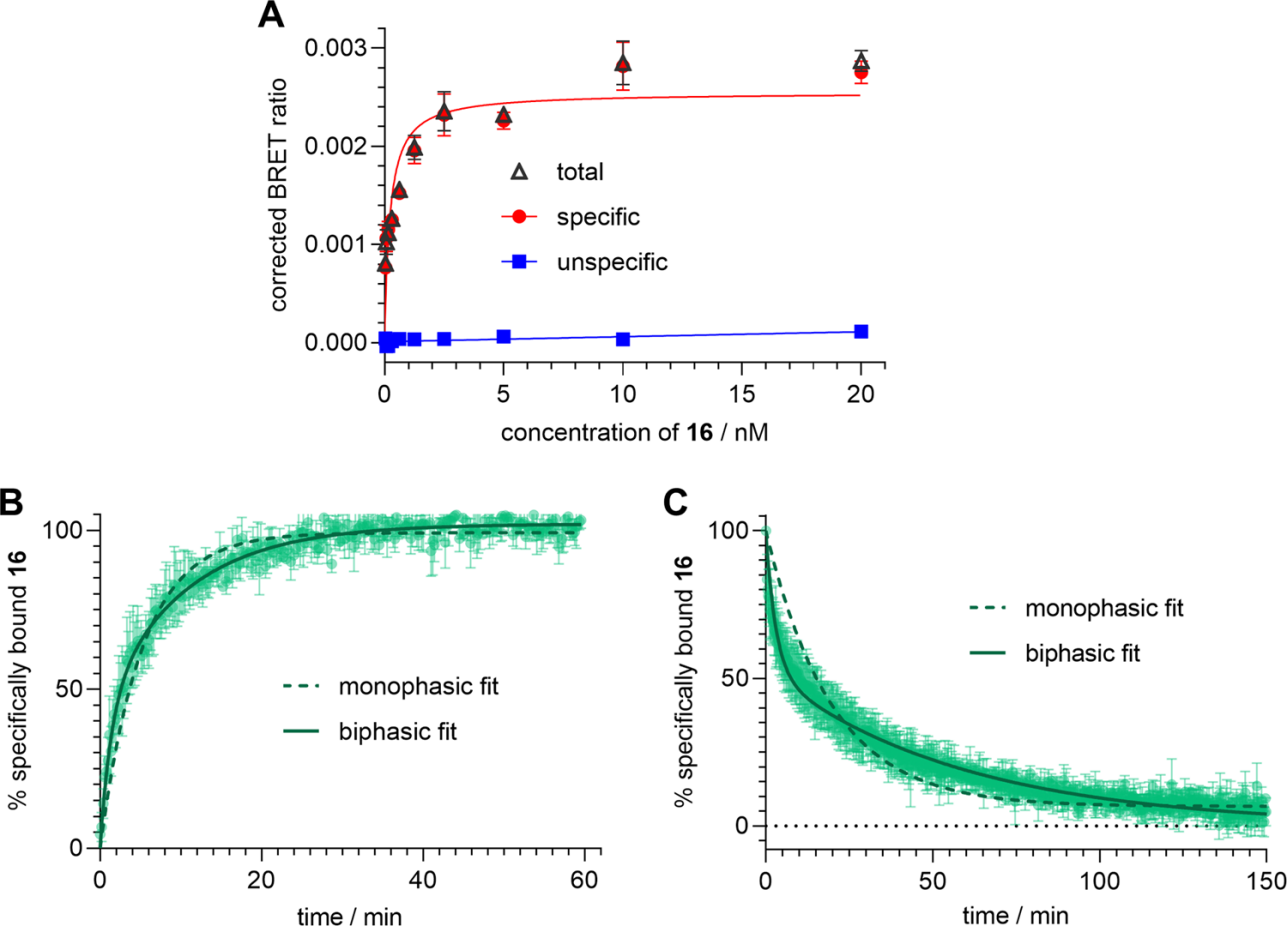
Characterization of Y_4_R binding of fluorescent ligand **16** in a NanoBRET-based binding assay at 25 °C using intact HEK293T-hY_4_R-NLuc(intraECL2) cells. (A) Representative saturation isotherm (specific binding) from saturation binding experiments. Unspecific binding was determined in the presence of 1 μM **5**. Total and unspecific binding data represent mean values ± SEM. Specific binding data represent calculated values ± propagated error. (B) Association of **16** (*c* = 1 nM) to Y_4_R. Mean values ± SEM from three independent experiments performed in triplicate. (C) Dissociation of **16** from Y_4_R. The dissociation was initiated after 1.5 h of preincubation with **16** (*c* = 3.5 nM) by the addition of a 1000-fold excess of **5**. Mean values ± SEM from three independent experiments performed in duplicate. Plateau value of the three-parameter fit describing a monophasic exponential decline: 6% (note: for the biphasic fit the plateau value was not different from zero, see discussion).

Notably, the association of **16** to Y_4_R was faster compared to flow cytometric and BRET-based kinetic binding studies, reaching a plateau after about 10 min (Figures 5A,C, 6B, and 7B), regardless of minor differences in assay temperatures (flow cytometry: 22 °C, FA: 27 °C, BRET: 25 °C). This could be attributed to the different expression systems (mammalian vs Sf9 insect cells). Y_4_Rs displayed by BBVs (FA assay) are highly unlikely to couple with insect cell Gα_i_-like proteins,^50^ thus a ternary complex, which would affect agonist receptor affinity, cannot be formed. As in the case of flow cytometric kinetic binding studies, the kinetically derived *K*_d_ value of **16** was lower than the equilibrium *K*_d_ (ca. a factor of 6 and 3 for Y_4_R^nonglyco^ and Y_4_R^SwBac^, respectively) (Table 5). Concerning the two variants of Y_4_R preparations (nonglycosylated vs putatively glycosylated), no marked differences in Y_4_R binding were observed. FA-based Y_4_R competition binding studies using **16** as a labeled ligand are discussed below.

### NanoBRET Y_4_R Binding Studies

**Figure 8:**
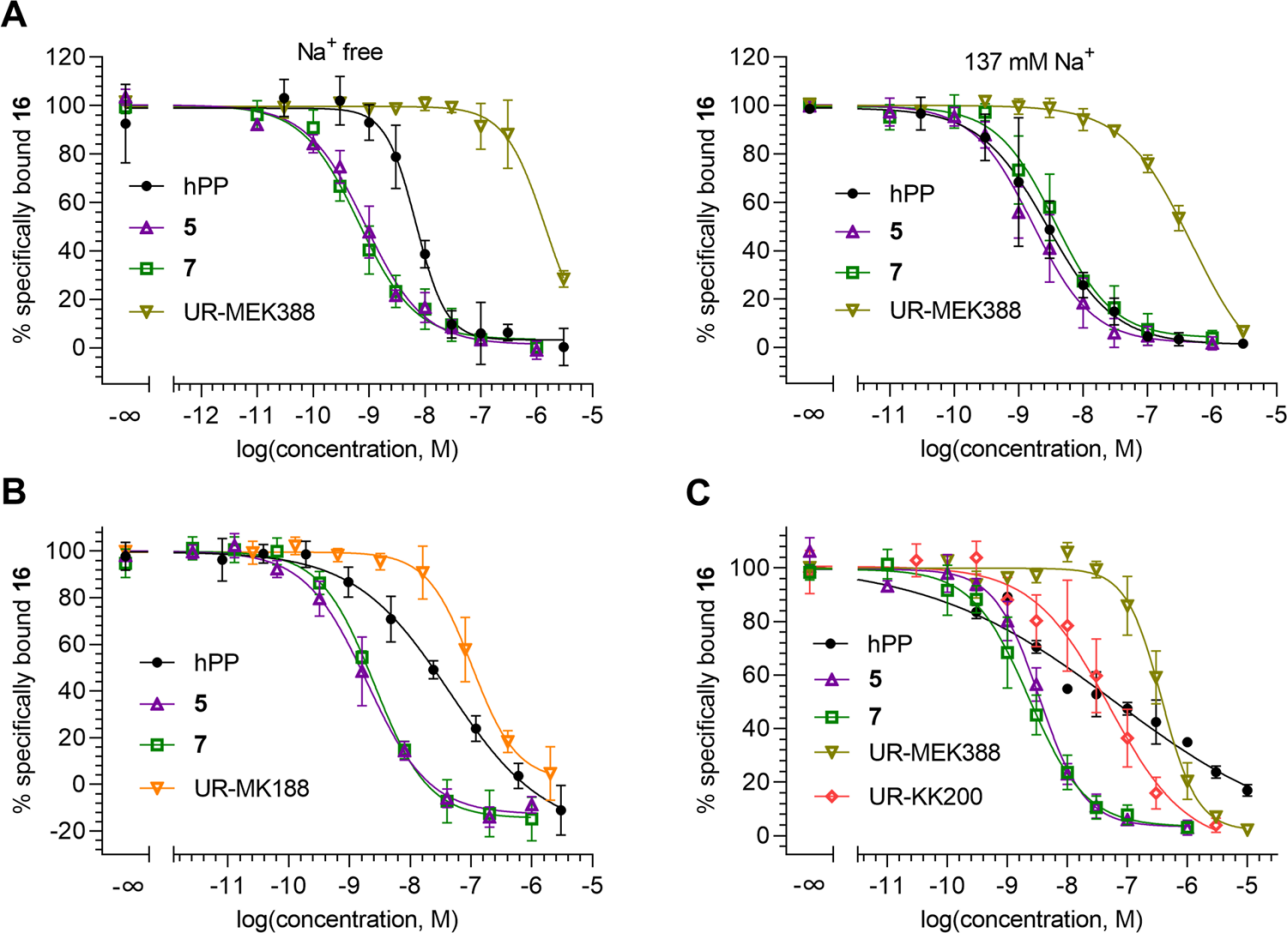
Determination of Y_4_R affinities of Y_4_R reference ligands (hPP, **5**, **7**, UR-MK188, UR-MEK388, UR-KK200) in different fluorescence-based assays by competition binding with **16**. (A) Displacement curves based on data from flow cytometric competition binding experiments performed with intact CHO-hY_4_R-G_qi5_-mtAEQ cells in sodium-free buffer (buffer I) and sodium-containing buffer (DPBS, 137 mM Na^+^). (B) Displacement curves from fluorescence anisotropy-based competition binding experiments performed with Y_4_R^SwBac^-displaying BBVs in DPBS. (C) Displacement curves from NanoBRET-based competition binding experiments performed with intact HEK293T-hY_4_R-NLuc(intraECL2) cells in L15-HEPES (140 mM Na^+^). Data (A–C), representing mean values ± SEM from three to five independent experiments performed in duplicate (B) or triplicate (A, C), were fitted according to a four-parameter logistic model.

To establish a Y_4_R NanoBRET binding assay, HEK293T cells were stably transfected with two different hY_4_R-Nluc fusion constructs, either with N-terminally fused Nluc (HEK293T-Nluc-hY_4_R cells) or with the Nluc inserted in the extracellular loop (ECL) 2 of the receptor (HEK293T-hY_4_R-Nluc(intraECL2) cells). Recently, BRET-based Y_1_R binding of a fluorescently labeled ligand at different Nluc-Y_1_R constructs was described. In these studies, the N-terminal fusion construct produced no BRET signal, whereas the intraECL2 construct gave high signals allowing the determination of equilibrium *K*_d_ values and kinetic data.^51^ Binding experiments with **16** at HEK293T-Nluc-hY_4_R cells and HEK293T-hY_4_R-Nluc(intraECL2) cells also resulted in considerably lower BRET signals for the former compared to the latter (data not shown), which can most likely be attributed to a higher distance between the Nluc and the Y_4_R ligand binding pocket with the N-terminal fusion construct. Therefore, the binding studies with **16** were performed with the HEK293T-hY_4_R-Nluc(intraECL2) cells using coelenterazine h or furimazine as the Nluc substrate and **5** for the determination of unspecific binding instead of hPP (used to block Y_4_R in radiochemical, flow cytometric and FA-based assays). This was necessary as the insertion of Nluc into ECL2 hampers Y_4_R binding of the large endogenous ligand hPP (see competition binding studies below, Figure 8).

Saturation binding with **16** at HEK293T-hY_4_R-Nluc(intraECL2) cells yielded a *K*_d_ value of 0.94 nM in good agreement with the *K*_d_ values obtained from flow cytometry and FA-based saturation binding assays (Tables 4–6 and Figure 7A).

**Table 6:** Parameters Characterizing Y_4_R Binding of **16** Determined in a NanoBRET Binding Assay at 25 °C

saturation binding	binding kinetics			
p*K*_d_/*K*_d_ [nM]^a^	*k*_obs_ [min^–1^]^b^	*k*_off_ [min^–1^]^c^	*k*_on_ [nM^–1^min^–1^]^d^	*K*_d_(kin) [nM]^e^
9.02 ± 0.04/0.94	mono: 0.18 ± 0.04	mono: 0.06 ± 0.01	mono: 0.18 ± 0.07	mono: 0.3 ± 0.2
	bi, fast: 0.58 ± 0.02	bi, fast: 0.34 ± 0.03	bi, fast: 0.24 ± 0.05	bi, fast: 1.4 ± 0.4
	bi, slow: 0.095 ± 0.005	bi, slow: 0.016 ± 0.002	bi, slow: 0.078 ± 0.008	bi, slow: 0.21 ± 0.05

a Equilibrium dissociation constant expressed as p*K*_d_ (mean values ± SEM) and *K*_d_ (mean value) obtained from three independent experiments (performed in triplicate).

b Observed association rate constants obtained by monophasic and biphasic fitting. Mean values ± SEM from four independent experiments (performed in triplicate).

c Dissociation rate constants obtained from three-parameter monophasic (*Y*_0_ constrained to 100%) and five-parameter biphasic (*Y*_0_ constrained to 100%, plateau constrained to zero) fits (exponential decline). Mean values ± SEM from three independent experiments (performed in triplicate).

d Association rate constant ± propagated error calculated from *k*_obs_, *k*_off_, and the ligand concentration used for the association studies.

e Kinetically derived dissociation constants ± propagated error calculated from various *k*_off_ and *k*_on_ values.

As in the case of flow cytometric association and dissociation studies, kinetic data from the BRET assay with **16** were analyzed by monophasic and biphasic fits (Figure 7B,C and Table 6). For both, association and dissociation experiments, the statistical Extra sum-of-squares *F*-test suggested that the data are better fitted by the biphasic fit compared to a monophasic analysis (two-phase association vs one-phase association and two-phase decay vs one-phase decay, GraphPad Prism 5) giving *P* values <0.0001. For the monophasic dissociation analysis, the plateau value was low (6%), but still significantly different from zero (*P* < 0.05, *t* test). In contrast, for the biphasic fit, the plateau value was not different from zero (*P* > 0.05, *t* test) indicating a complete dissociation of **16** from the Y_4_R (Figure 7B,C). Possible reasons for a biphasic association were discussed in the section describing flow cytometric binding studies (see above). Like the biphasic association, the biphasic dissociation may also be explained by the existence of different receptor states.^47^ However, since a monophasic dissociation curve was obtained from flow cytometric dissociation experiments with **16** (cf. Figure 5B), the cell type (flow cytometry: CHO cells, NanoBRET: HEK293T cells) and/or the receptor variant (flow cytometry: wild-type Y_4_R, NanoBRET: Y_4_R-Nluc construct (Nluc inserted in ECL2)) seem to be relevant for the association. Recently, a metadynamics protocol for binding and unbinding of peptide ligands of class A GPCRs suggested that Y_4_R agonists also bind with high affinity to the vestibule-like binding site of Y_4_R, located on top of the orthosteric binding site.^52^ Consequently, in the dissociation process, the ligand might be withheld by residing in the vestibule of the receptor. Depending on the receptor conformation and possibly additionally triggered by the presence of Nluc in the ECL2 of the Y_4_R, the residence time in the vestibule might vary, resulting in different dissociation rates as observed in the NanoBRET assay.

The *K*_d(kin)_ values of **16** were calculated from *k*_on(bi,fast)_ and *k*_off(bi,fast)_ as well as from *k*_on(bi,slow)_ and *k*_off(bi,slow)_, and additionally from *k*_on(mono)_ and *k*_off(mono)_ as the monophasic fits cover the whole association and dissociation process. Moreover, the biphasic character was generally weak (minor differences in *k* values, see Table 6). Interestingly, the *K*_d(kin)_ value derived from *k*_on(bi,fast)_ and *k*_off(bi,fast)_ was in good agreement with the *K*_d_ value from saturation binding studies. The *K*_d_(kin) value obtained from *k*_on(mono)_ and *k*_off(mono)_ and the *K*_d(kin)_ calculated from *k*_on(bi,slow)_ and *k*_off(bi,slow)_ were similar and slightly lower (factor 3–5) compared to the *K*_d_ obtained from saturation binding experiments (Table 6). Consequently, irrespective of the weak biphasic nature of the association and dissociation, Y_4_R binding of **16** studied in the NanoBRET assay approximatively follows the law of mass action.

BRET-based saturation binding experiments at HEK293T-hY_4_R-Nluc(intraECL2) cells were also performed with the Py-1 labeled ligand **18**. Although the excitation spectrum of the Py-1 conjugate **18** shows a higher overlap with the Nluc emission spectrum (λ_max_ ca. 460 nm)^53^ compared to the excitation spectrum of the Cy3B-labeled ligand **16** (cf. Table 3), considerably lower BRET signals compared to experiments carried out with **16** (data not shown) were obtained, which can be explained by the markedly higher fluorescence quantum yield of **16** compared to **18** (see Table 3). NanoBRET Y_4_R competition binding studies using **16** as labeled ligand are discussed below.

### Fluorescence-Based Y_4_R Competition Binding

Using fluorescent ligand **16** as labeled probe, Y_4_R affinities of previously reported Y_4_R ligands (hPP, **5**, **7** and UR-MEK388 or UR-MK188) were determined in flow cytometric, FA- and BRET-based binding assays (displacement curves shown in Figure 8). To note, the dimeric argininamide-type Y_4_R ligands UR-MEK388 and UR-MK188 represent enantiomers ((*S*,*S*)- and (*R*,*R*)-configuration, respectively) displaying almost equal Y_4_R affinity and Y_4_R antagonism (Ca^2+^ aequorin assay).^54^ Additionally, the Y_4_R partial agonist UR-KK200^35^ was studied in the NanoBRET assay.

The obtained Y_4_R affinities of hPP were consistently lower than affinities determined in radiochemical assays (Table 7), with the lowest discrepancy (ca. one log unit) found in the case of the flow cytometric binding studies performed in DPBS. The lowest hPP affinity was determined in the NanoBRET-based competition binding assay, producing a shallow curve with a very low hill slope of −0.3 (Figure 8C). This shows that the insertion of Nluc into ECL2 affects the binding of hPP to Y_4_R. Similar to hPP, the obtained p*K*_i_ values of the cyclic peptides **5** and **7** were lower by approximately 1 order of magnitude compared to reported p*K*_i_ values determined in radioligand competition binding assays. The Y_4_R affinity of UR-KK200, determined in the NanoBRET assay, was in good agreement with the reported data (radiochemical assay). Y_4_R binding data, obtained for UR-MEK388 (flow cytometric and NanoBRET assays) were also in accordance with a reported p*K*_i_ value determined in a flow cytometric competition binding assay using Sulfo-Cy5-labeled [K^4^]hPP as labeled ligand (Table 7). The Y_4_R affinity of UR-MK188, determined in the FA competition binding assay, was slightly higher compared to the reported binding data of UR-MK188. Generally, the lowest discrepancies between the determined and reported binding data of Y_4_R reference ligands were found for the NanoBRET assay. As **16** showed a complete dissociation from the Y_4_R only in the case of the NanoBRET assay, the incomplete dissociation observed in the flow cytometric and FA binding assay could be a reason for the observed discrepancies between reported Y_4_R affinities and binding data determined with **16** in flow cytometric and FA-based competition binding assays: to achieve a complete “displacement” of the fluorescent ligand **16**, the competing ligand, incapable of displacing (pseudo)irreversibly bound **16** from the receptor, must be used at higher concentrations (compared to the situation where the labeled ligand shows full reversible binding), resulting in a higher receptor occupancy and thus effectively preventing binding of **16** to the receptor. This phenomenon was also clearly observed for radiolabeled muscarinic M_2_ receptor ligands showing different dissociation kinetics (reversible and (pseudo)irreversible binding): competition binding studies with reference ligands yielded consistently lower p*K*_i_ values for the nonreversibly binding radioligand.^55^

**Table 7:** Overview of hY_4_R Binding Affinities of Y_4_R Reference Ligands Determined in Fluorescence-Based Competition Binding Assays Using **16** as Labeled Probe

	flow cytometry^a^		FA^b^	nanoBRET^c^	literature	
	Na^+^ free	137 mM Na^+^	137 mM Na^+^	140 mM Na^+^	Na^+^ free	137 mM Na^+^
cmpd.	p*K*_i_					
hPP	8.5 ± 0.1	8.9 ± 0.1	8.0 ± 0.2	7.7 ± 0.1	10.02^d^	10.1^e^
**5**	9.49 ± 0.07	9.15 ± 0.08	9.26 ± 0.04	8.89 ± 0.06	10.48^f^	n.a.
**7**	9.61 ± 0.06	8.8 ± 0.1	9.4 ± 0.1	9.07 ± 0.07	10.5^e^	9.79^e^
UR-KK200	n.d.	n.d.	n.d.	7.8 ± 0.1	8.92^g^	7.99^e^
UR-MEK388	6.29 ± 0.08	6.68 ± 0.09	n.d.	6.85 ± 0.05	6.58^h^	n.a.
UR-MK188	n.d.	n.d.	7.7 ± 0.1	n.d.	6.88^h^	6.82^g^
					6.18^g^	

a Determined at intact CHO-hY_4_R-G_qi5_-mtAEQ cells; mean values ± SEM from three or four independent experiments performed in triplicate.

b Determined at Y_4_R^SwBac^ displaying BBVs; mean values ± SEM from three independent experiments performed in duplicate.

c Determined at intact HEK293T-hY_4_R-NLuc(intraECL2) cells; mean values ± SEM from three or four independent experiments performed in triplicate.

d Wirth et al.^37^

e Gleixner et al.^31^

f Konieczny et al.^30^

g Kuhn et al. (reported *K*_i_ values were converted to p*K*_i_).^35^

h Keller et al. (reported *K*_i_ values were converted to p*K*_i_).^54^ Note: UR-MEK388 and UR-MK188 represent enantiomers exhibiting almost equal Y_4_R affinity.^54^ n.a. not available.

### Microscopy Studies

**Figure 9:**
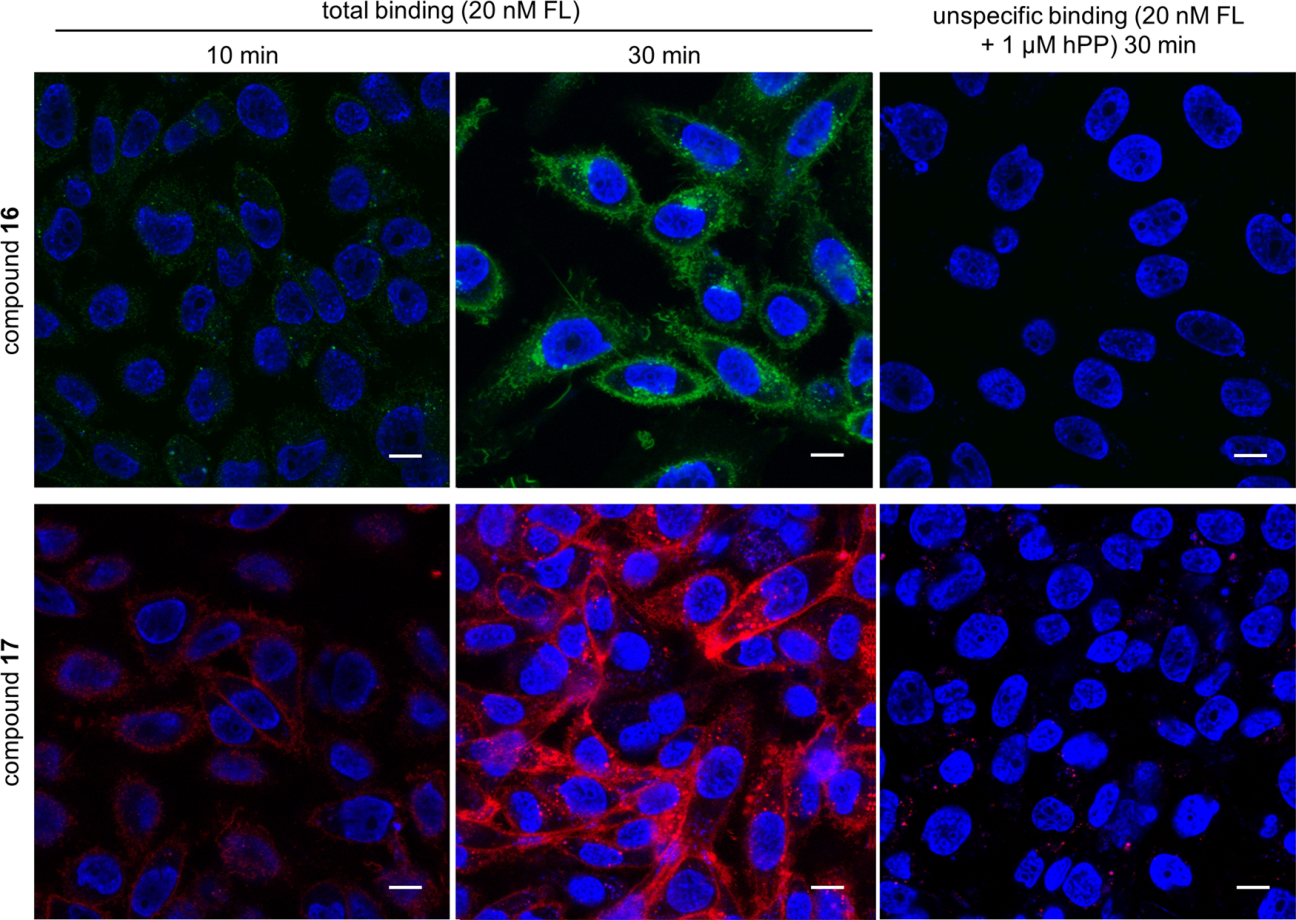
Visualization of fluorescent ligand (**16**, **17**) binding to CHO-hY_4_R-G_qi5_-mtAEQ cells by confocal microscopy. Shown are representative images acquired after incubation of the cells with **16** or **17** (each 20 nM) at 22 °C for 10 and 30 min. Unspecific binding was determined in the presence of 1 μM hPP. Nuclei were stained with H33342 (2 μM). Fluorescence of **16** and **17** is shown in green and red, respectively. Fluorescence of H33342 is shown in blue. Scale bar: 10 μm.

Y_4_R binding of **16** and **17** was visualized by confocal microscopy using intact CHO-hY_4_R-G_qi5_-mtAEQ cells. Cellular uptake of both fluorescent probes was evident after already 10 min of incubation at 22 °C (Figure 9). After 30 min, the intracellular and plasma membrane-associated fluorescence was higher compared to 10 min after start of incubation, which is in agreement with the association kinetics determined by flow cytometry (Figure 5A). Notably, in the presence of an excess of hPP, almost no ligand′s fluorescence could be detected (unspecific binding, Figure 9). This indicates that the cellular uptake of the fluorescent ligands, which appear to be located in vesicles, is caused by Y_4_R-mediated endocytosis. Similar results were previously observed with the Sulfo-Cy5 labeled hPP derivative **1** studied with the same cells.^26^

**Figure 10:**
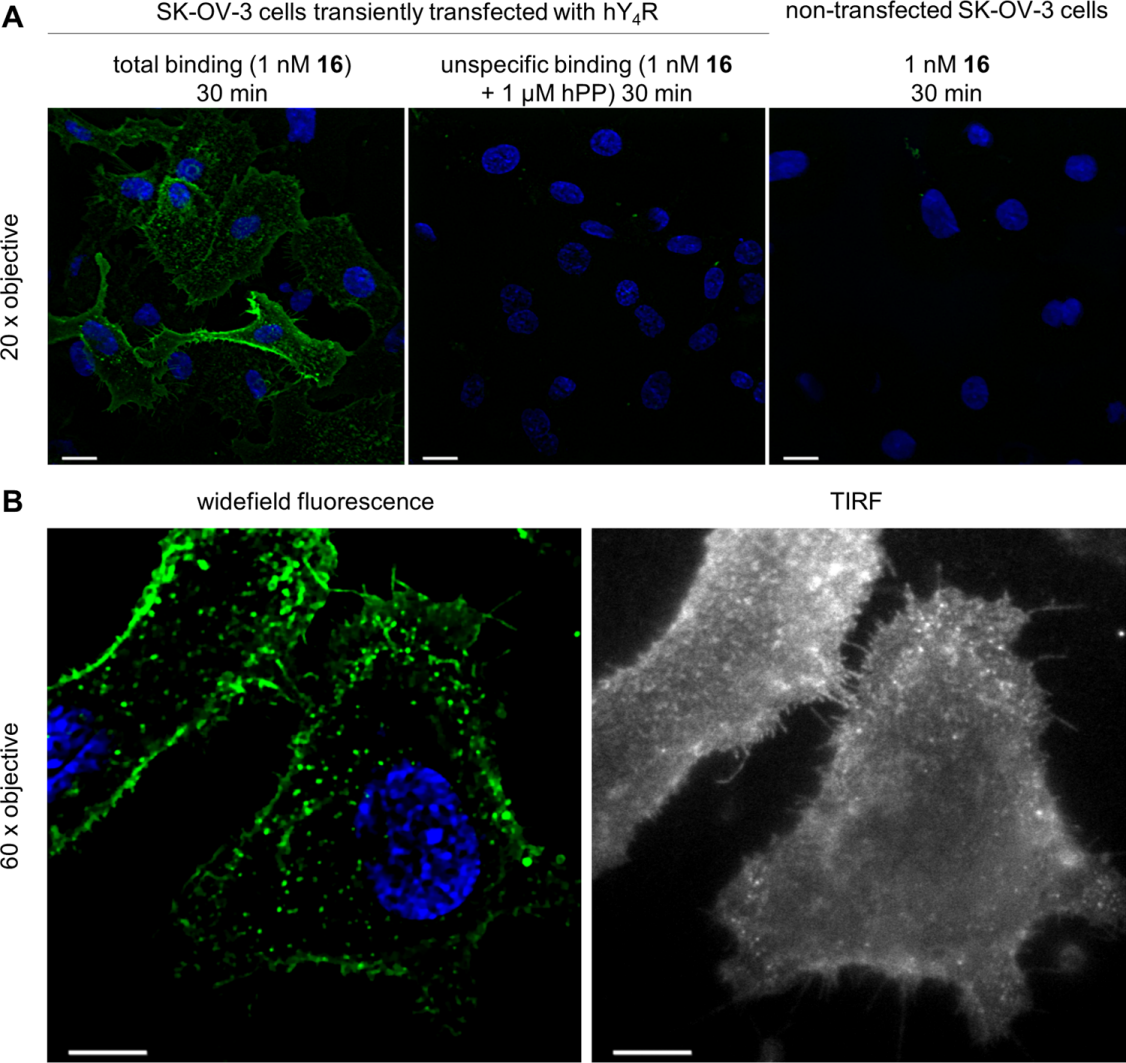
Visualization of binding of **16** to the hY_4_R transiently expressed by SK-OV-3 cells using wide-field and TIRF microscopy. (A) Wide-field fluorescence images acquired after incubation of the cells with **16** at 37 °C for 30 min. The two-color composite of individual focal planes after Z-stack deconvolution is shown with the green pseudocolor for **16** (561 nm excitation) and the blue pseudocolor for the nuclear stain channel (Hoechst 34580, 405 nm excitation). (B) Wide-field fluorescence and TIRF images of the same cells obtained after incubation of the cells with **16** (1 nM) at 37 °C for 30 min. Wide-field images were processed as under (A). In TIRF images, fluorescence of **16** is shown in white pseudocolor. Scale bar: 10 μm.

Y_4_R binding of the Cy3B-labeled ligand **16** was also studied at transiently transfected SK-OV-3 cells using wide-field and TIRF microscopy. For these experiments, a lower concentration of **16** (1 nM), compared to confocal microscopy (20 nM), was used, resulting in a lower receptor occupancy. As also observed for binding of **16** to CHO-hY_4_R-G_qi5_-mtAEQ cells (confocal microscopy), unspecific binding was very low (Figure 10A).

As an additional control, binding of **16** to nontransfected SK-OV-3 cells was investigated, also revealing very low unspecific binding. Thus, the fluorescence of **16** detected in the total binding samples represents ligand **16** bound to Y_4_R. The Y_4_R appeared to be largely associated with the plasma membrane of the cells (Figure 10A).

Using a higher magnification (60× objective), intracellular fluorescence could be detected (Figure 10B). In the TIRF images, also binding of the ligand to Y_4_R located in plasma membrane protrusions (filopodia) was visible. Notably, fluorescent ligand **16** proved to be sufficiently photostable and bright enough for single-molecule tracking studies by TIRF. This was demonstrated by binding of **16** to Y_4_R expressing SK-OV-3 cells using a low fluorescent ligand concentration of 0.1 nM (time lapse TIRF imaging sequence recorded at 30 Hz resolution; video, shown at double speed, is available as Supporting Information). Various diffusion modes could be observed along the particle tracks. Here, events of continuous confinement as well as transient confinement (when molecules diffused in a grid of impermeable barriers) were identified. The unconfined trajectories can be classified as having mostly random diffusion, except for the filopodia where directional diffusion with high linearity was observed. A deeper analysis of the trajectories is beyond the scope of this article.

## Conclusions

Conjugation of different fluorescent dyes to recently discovered small cyclic peptides, exhibiting high Y_4_R binding and selectivity, produced fluorescent Y_4_R ligands with unprecedented Y_4_R affinity (*K*_i_, *K*_d_ < 1 nM). Notably, the cyclic peptides contained one or two *N*^ω^-carbamoylated arginines. These modified arginines were recently introduced as bioisosteric replacements of natural arginine in arginine-containing peptides enabling radio- and fluorescence labeling of bioactive peptides via arginine residues.^32,35,39,56,57^ The present work further demonstrates the usefulness of *N*^ω^-carbamoylated arginines with respect to the design and preparation of labeled probes for peptidergic receptors. The study also suggests that a broad variety of fluorescent dyes and other bulky moieties can be attached to the cyclic peptides without marked impact on Y_4_R binding. Therefore, further tool compounds with high Y_4_R affinity and selectivity are conveniently accessible according to the presented strategy. The preparation of new fluorescent ligands might be of interest, for example, in improving the physicochemical properties (use of more polar fluorescent dyes) or an adjustment of the photophysical properties for the intended application. Nonetheless, the presented fluorescent probes are useful for the determination of Y_4_R binding affinities in fluorescence-based competition binding assays and, thus, could be used as tools in drug development programs aiming for therapeutics acting at Y_4_R.

## Experimental Section

### Materials

The protected amino acids Fmoc-Tyr(tBu)–OH, Fmoc-Leu−OH, and Fmoc-Arg(Pbf)–OH were purchased from Carbolution Chemicals (St. Ingbert, Germany). HBTU, Fmoc-Sieber PS Resin, and PyBOP were obtained from Iris Biotech (Marktredwitz, Germany). Copper(II)sulfate pentahydrate, HOBt, sodium ascorbate, and Triton X-100 were from Sigma-Aldrich (Taufkirchen, Germany). NMP and DMF for peptide synthesis, anhydrous DMF, dichloromethane, piperidine, succinic anhydride, trifluoroacetic acid (TFA), and tetrahydrofuran were purchased from ACROS/Fisher Scientific (Schwerte, Germany). DIPEA was obtained from TCI (Eschborn, Germany). *tert*-Butyl (3-bromopropyl)carbamate (**20**) was from ABCR (Karlsruhe, Germany). Acetonitrile (high-performance liquid chromatography (HPLC) gradient grade) was from VWR (Ismaning, Germany). The fluorescent dye **13** (S0586, succinimidyl ester of S0223) was from FEW chemicals (Wolfen, Germany). Human pancreatic polypeptide (hPP) and porcine neuropeptide Y (pNPY) were purchased from Synpeptide (Shanghai, China). Bacitracin and bovine serum albumin (BSA) were obtained from Serva (Heidelberg, Germany). The Nluc substrate furimazine was purchased from Promega (Walldorf, Germany). Coelenterazine h was from BIOTREND (Köln, Germany). Zeocine and Puromycine were from Invivogen (San Diego, CA), Hygromycin B and propidium iodide were from Carl Roth (Karlsruhe, Germany) and Geneticine G418 was from Fisher Scientific (Schwerte, Germany). The Y_2_R antagonist BIIE0246 was obtained from Boehringer Ingelheim (Ingelheim, Germany). The syntheses of the Y_1_R antagonists BIBO3304^41^ and [^3^H]UR-MK299 (molar activity: 1.81 TBq/mmol),^33^ and the synthesis of the Y_4_R ligands **4**,^30^**5**,^30^**6**,^31^**7**,^31^ and [^3^H]UR-KK200 (molar activity: 0.98 TBq/mmol)^35^ were described previously. [^3^H]Propionyl-pNPY (molar activity: 1.39 TBq/mmol) was prepared according to a previously reported procedure^33^ with minor modifications that were described elsewhere.^40^ Compounds **8**(32) and **9**,^29^ the fluorescent dyes **12** (Cy3B-SE),^38^**14** (Py-1),^58^ and **22** (Py-5),^58^ as well as the fluorescent dummy ligands **23**(38) and **24**(39) were prepared according to reported procedures. Millipore water was consistently used for the preparation of stock solutions, buffers, and aqueous eluents for HPLC. Polypropylene reaction vessels (1.5 and 2 mL) from Sarstedt (Nümbrecht, Germany) were used to keep stock solutions and for small-scale reactions (e.g., fluorescence labeling reactions and activation of Fmoc-protected amino acids for peptide synthesis).

### NMR Spectroscopy

NMR spectra were recorded on a Bruker AVANCE 400 (^1^H, 400 MHz; ^13^C, 100 MHz) or on an AVANCE 600 instrument with cryogenic probe (^1^H: 600 MHz and ^13^C: 150 MHz) (Bruker, Karlsruhe, Germany). NMR spectra were calibrated based on the solvent residual peaks (^1^H NMR, DMSO-*d*_6_: δ = 2.50 ppm; ^13^C NMR: DMSO-*d*_6_: δ = 39.50 ppm), and data are reported as follows: ^1^H NMR: chemical shift δ in ppm (multiplicity [s = singlet, d = doublet, t = triplet, m = multiplet, and br s = broad singlet], integral, coupling constant *J* in Hz).

### Mass Spectrometry

High-resolution mass spectrometry (HRMS) was performed with an Agilent 6540 UHD accurate-mass quadrupole time-of-flight (Q-TOF) liquid chromatography/mass spectrometry (LC/MS) system coupled to an Agilent 1290 analytical HPLC system (Agilent Technologies. Santa Clara, CA) using an ESI source and the following LC method: column: Luna Omega C18, 1.6 μm, 50 mm × 2.1 mm (Phenomenex, Aschaffenburg, Germany), column temperature: 40 °C, solvent/linear gradient: 0–4 min: 0.1% aqueous HCOOH/acetonitrile supplemented with 0.1% HCOOH 95:5–2:98, 4–5 min: 2:98, flow: 0.6 mL/min.

### Preparative HPLC

Preparative HPLC was performed with a system from Knauer (Berlin, Germany) consisting of two K-1800 pumps and a K-2001 detector (in the following referred to as “system 1”) or with a Prep 150 LC system from Waters (Eschborn, Germany) comprising a Waters 2545 binary gradient module, a Waters 2489 UV/vis detector, and a Waters fraction collector III (in the following referred to as “system 2”). A Gemini NX-C18, 5 μm, 250 mm × 21 mm (Phenomenex) was used as a reversed-phase (RP) column at a flow rate of 20 mL/min using mixtures of 0.1% aqueous TFA and acetonitrile as the mobile phase. A detection wavelength of 220 nm was used throughout. Collected fractions were lyophilized using a Scanvac CoolSafe 100–9 freeze-dryer (Labogene, Allerød, Denmark) equipped with an RZ 6 rotary vane vacuum pump (Vacuubrand, Wertheim, Germany).

### Analytical HPLC

Analytical HPLC analysis was performed with a system from Agilent Technologies composed of a 1290 Infinity binary pump equipped with a degasser, a 1290 Infinity autosampler, a 1290 Infinity thermostated column compartment, a 1260 Infinity diode array detector, and a 1260 Infinity fluorescence detector. A Kinetex-XB C18, 2.6 μm, 100 mm × 3 mm (Phenomenex) served as the stationary phase at a flow rate of 0.6 mL/min. Detection was performed at 220 nm, and the oven temperature was 25 °C. Mixtures of 0.04% aqueous TFA (A) and acetonitrile (B) were used as mobile phase. The following linear gradients were applied: for compounds **11** and **19**: 0–14 min: A/B 90:10–70:30, 14–16 min: 70:30–5:95, 16–20 min: 5:95 (isocratic); for compounds **16**–**18**: 0–14 min: A/B 90:10–65:35, 14–16 min: 65:35–5:95, 16–20 min: 5:95 (isocratic). The injection volume was 20 μL. Retention (capacity) factors *k* were calculated from the retention times *t*_R_ according to *k* = (*t*_R_ – *t*_0_)/*t*_0_ (*t*_0_ = dead time, 0.76 min for the system, column, and flow rate mentioned above).

#### Compound Characterization

The linear precursor peptide **10**, synthesized by solid-phase peptide synthesis, was characterized by HRMS. Peptide **11** was characterized by HRMS, ^1^H NMR spectroscopy, and RP-HPLC. The azido-functionalized fluorescent dye **15** was characterized by HRMS and ^1^H NMR spectroscopy. The fluorescent ligands **16**–**19** were characterized by HRMS and RP-HPLC. HPLC purities of all target compounds were ≥95% (UV detection, 220 nm).

#### Synthesis of Peptide **11** and the Fluorescent Ligands **16**–**19**

##### *N*^α^-Succinyl-*N*^ω^[hex-5-ynylaminocarbonyl]-Arg-Tyr-*N*^ω^-[(aminobutyl)aminocarbonyl] Arg-Leu-Arg-Tyr-Amide Tetrakis(hydrotrifluoroacetate) (**10**)

Peptide **10** was synthesized on a Fmoc-Sieber PS resin (100 mg, 0.59 mmol/g) by manual Fmoc strategy SPPS using DMF/NMP (both anhydrous) 4:1 v/v as solvent. 5 mL NORM-JECT syringes (B. Braun Melsungen, Melsungen, Germany), equipped with a 35 μm polypropylene frit (Roland Vetter Laborbedarf, Ammerbuch, Germany), were used as reaction vessels. The resin was allowed to swell in solvent for 45 min at room temperature followed by initial Fmoc deprotection using a 20% piperidine solution in DMF/NMP 4:1 v/v (2 × 20 min at rt). The contained standard Fmoc amino acids (Tyr, Arg, Leu) were used in 5-fold excess and preactivated with HOBt/HBTU/DIPEA (5/4.9/10 equiv) in solvent (about 2.2 mL/mmol aa) for at least 5 min before addition to the resin. For the activation of building blocks **8** and **9**, used in 3-fold excess, HBTU/HOBt/DIPEA (3/3/6 equiv) were used. Amino acid coupling was carried out on a shaker (Heidolph Multi Reax; Heidolph Instruments, Schwabach, Germany) covered with a thermostat-controlled (38 °C) box. In the case of standard (proteinogenic) amino acids, “double” coupling (2 × 45 min) was performed. Arginine derivatives **8** and **9** were attached by a single coupling procedure (16 h). After coupling, Fmoc deprotection was carried out using a 20% piperidine solution in DMF/NMP 4:1 v/v for 2 × 10 min at rt. The resin was washed 6 times with about 1 mL of solvent after Fmoc deprotection and 4 × 1 mL after amino acid coupling. After coupling of the last amino acid and subsequent Fmoc deprotection, the resin was treated with a solution of succinic anhydride (52.3 mg, 0.443 mmol) and DIPEA (77.1 μL, 0.443 mmol) in DMF/NMP 80:20 v/v (500 μL) at 35 °C for 30 min. The resin was washed with DMF/NMP 4:1 v/v (6×) and CH_2_Cl_2_ (3×), followed by cleavage off the resin. For this, a mixture of trifluoroacetic acid (TFA) and DCM (3:1 v/v) was added to the resin and the mixture was shaken for 20 min. The fluid was collected in a 100 mL round-bottom flask, and the procedure was repeated once. The resin was afterward washed 3 times with cleavage solution, and the volatiles were removed in vacuo. Full side-chain deprotection was carried out with 95% aq. TFA (2 mL) for 5 h at rt. The reaction mixture was concentrated in vacuo, then water (40 mL) was given to the mixture, followed by lyophilization. Purification by preparative HPLC (system 1, gradient: 0–18 min: 0.1% aq TFA/acetonitrile 95:5–55:45, *t*_R_ = 15 min) yielded **10** as a white fluffy solid (37.65 mg, 37%). HRMS (ESI): *m*/*z* [M + 2H]^2+^ calcd. for [C_58_H_93_N_19_O_13_]^2+^ 631.8595, found: 631.8616. C_58_H_91_N_19_O_13_·C_8_H_4_F_12_O_8_ (1262.49 + 456.09).

##### (9*S*,12*S*,15*S*,*E*)-4-Amino-*N*-{(*S*)-1-[((*S*)-1-{[(*S*)-1-amino-3-(4-hydroxyphenyl)-1-oxopropan-2-yl]amino}-5-guanidino-1-oxopentan-2-yl)amino]-4-methyl-1-oxopentan-2-yl}-15-{3-[(*E*)-2-(hex-5-yn-1-ylcarbamoyl)guanidino]propyl}-12-(4-hydroxybenzyl)-2,11,14,17,20-pentaoxo-1,3,5,10,13,16,21-heptaazacyclopentacos-3-ene-9-carboxamide Tris(hydrotrifluoroacetate) (**11**)

Peptide **10** (8.89 mg, 7.13 μmol) was dissolved in anhydrous DMF (5.7 mL), a solution of HOBt (2.89 mg, 21.4 μmol) in DMF (0.6 mL) and DIPEA (7.5 μL, 42.8 μmol) were added and the mixture was stirred at rt for 5 min. A solution of PyBOP (11.1 mg, 21.4 μmol) in DMF (0.8 mL) was added dropwise and stirring was continued at rt for 16 h. 1% aqueous TFA (10 mL) was added and the product was purified by preparative HPLC (system 1, gradient: 0–18 min: 0.1% aq TFA/acetonitrile 95:5–55:45, *t*_R_ = 16 min) to yield **11** as a white fluffy solid (2.39 mg, 21%) ^1^H NMR (600 MHz, DMSO-*d*_6_): δ (ppm) 0.82–0.91 (m, 6H), 1.33–1.49 (m, 12H), 1.49–1.59 (m, 7H), 1.59–1.70 (m, 3H), 1.70–1.79 (m, 1H), 2.15–2.2 (m, 2H), 2.29–2.41 (m, 3H), 2.68–2.74 (m, 1H), 2.74–2.81 (m, 2H), 2.81–2.89 (m, 2H), 2.89–2.97 (m, 1H), 3.02–3.08 (m, 3H), 3.09–3.14 (m, 3H), 3.17–3.21 (m, 3H), 3.28–3.32 (m, 3H), 4.05–4.12 (m, 1H), 4.16–4.23 (m, 1H), 4.25–4.42 (m, 4H), 6.44–6.59 (br s, 1H), 6.61–6.68 (m, 4H), 6.68–6.96 (br s, 2H), 6.95–7.03 (m, 4H), 7.04–7.07 (m, 1H), 7.09–7.48 (br s, 2H, interfering with the next listed signal), 7.35 (s, 1H), 7.50 (t, 1H, *J* 5.4 Hz), 7.54–7.62 (m, 2H), 7.75 (d, 1H, *J* 7.9 Hz), 7.83 (d, 1H, *J* 7.5 Hz), 7.87–7.94 (m, 2H), 7.97 (d, 1H, *J* 7.3 Hz), 8.05–8.11 (m, 1H), 8.11–8.21 (m, 1H), 8.23–8.6 (m, 3H), 8.95 (s, 1H), 9.09–9.25 (m, 3H), 9.85 (s, 1H), 10.0 (s, 1H). HRMS (ESI): *m*/*z* [M + 3H]^3+^ calcd. for [C_58_H_92_N_19_O_12_]^3+^ 415.5719, found: 415.5725 RP-HPLC (220 nm): 98% (*t*_R_ = 10.7 min, *k* = 13.0). C_58_H_89_N_19_O_12_·C_6_H_3_F_9_O_6_ (1244.47 + 342.07).

##### 24-{5-[(4-{[(9*S*,12*S*,15*S*,19*R*,*E*)-4-Amino-9-({(*S*)-1-[((*S*)-1-{[(*S*)-1-amino-3-(4-hydroxyphenyl)-1-oxopropan-2-yl]amino}-5-guanidino-1-oxopentan-2-yl)amino]-4-methyl-1-oxopentan-2-yl}carbamoyl)-15-(3-guanidinopropyl)-12-(4-hydroxybenzyl)-2,11,14,17,20-pentaoxo-1,3,5,10,13,16,21-heptaazacyclopentacos-3-en-19-yl]amino}-4-oxobutyl)amino]-5-oxopent-1-yn-1-yl}-5,5,27,27-tetramethyl-16-oxa-20-aza-12-azoniaheptacyclo[15.11.0.0^3,15^.^04,12^.0^6,11^.0^20,28^.0^21,26^]octacosa-1(28),2,4(12),6(11),7,9,21(26),22,24-nonaene-8-sulfonate Bis(hydrotrifluoroacetate) (**16**)

Peptide **6** (1.05 mg, 0.626 μmol) was dissolved in anhydrous DMF (100 μL) followed by the addition of DIPEA (0.88 μL, 5.00 μmol). A solution of **12** (0.49 mg, 0.626 μmol) in DMF (64 μL) was added, and the mixture was shaken at rt in the dark for 1.5 h. 100 μL of 1% aqueous TFA were added and the mixture was subjected to preparative HPLC (system 1, gradient: 0–20 min: 0.1% aq TFA/acetonitrile 90:10–50:50, *t*_R_ = 11 min) to yield **16** as a purple fluffy solid (0.40 mg, 35%). HRMS (ESI): *m*/*z* [M + 2H]^2+^ calcd. for [C_89_H_122_N_22_O_17_S]^2+^ 901.9548, found: 901.9555 RP-HPLC (220 nm): 99% (*t*_R_ = 10.4 min, *k* = 12.7). C_89_H_120_N_22_O_17_S·C_4_H_2_F_6_O_4_ (1802.14 + 228.04).

##### 4-(2-{(1*E*,3*E*)-5-[(*E*)-1-(6-{[4-({(9*S*,12*S*,15*S*,19*R*,*E*)-4-Amino-9-[((*S*)-1-{[(*S*)-1-({(*S*)-1-amino-3-(4-hydroxyphenyl)-1-oxopropan-2-yl}amino)-5-guanidino-1-oxopentan-2-yl]amino}-4-methyl-1-oxopentan-2-yl)carbamoyl]-15-(3-guanidinopropyl)-12-(4-hydroxybenzyl)-2,11,14,17,20-pentaoxo-1,3,5,10,13,16,21-heptaazacyclopentacos-3-en-19-yl}amino)-4-oxobutyl]amino}-6-oxohexyl)-3,3-dimethyl-5-sulfoindolin-2-ylidene]penta-1,3-dien-1-yl}-3,3-dimethyl-3*H*-indol-1-ium-1-yl)butane-1-sulfonate Bis(hydrotrifluoroacetate) (**17**)

Peptide **6** (1.05 mg, 0.626 μmol) was dissolved in anhydrous DMF (100 μL) followed by the addition of DIPEA (0.88 μL, 5.00 μmol). A solution of **13** (0.50 mg, 0.626 μmol) in DMF (100 μL) was added, and the mixture was shaken at rt in the dark for 1.5 h. 100 μL of 1% aqueous TFA were added and the mixture was subjected to preparative HPLC (system 1, gradient: 0–20 min: 0.1% aq TFA/acetonitrile 90:10–50:50, *t*_R_ = 12 min) affording **17** as a blue fluffy solid (0.82 mg, 70%). HRMS (ESI): *m*/*z* [M + 2H]^2+^ calcd. for [C_90_H_132_N_22_O_19_S_2_]^2+^ 944.4735, found: 944.4747. RP-HPLC (220 nm): 96% (*t*_R_ = 11.6 min, *k* = 14.3). C_90_H_130_N_22_O_19_S_2_·C_4_H_2_F_6_O_4_ (1888.29 + 228.04).

##### 1-{4-[((9*S*,12*S*,15*S*,19*R*,*E*)-4-Amino-9-{[(*S*)-1-({(*S*)-1-[((*S*)-1-amino-3-(4-hydroxyphenyl)-1-oxopropan-2-yl)amino]-5-guanidino-1-oxopentan-2-yl}amino)-4-methyl-1-oxopentan-2-yl]carbamoyl}-15-(3-guanidinopropyl)-12-(4-hydroxybenzyl)-2,11,14,17,20-pentaoxo-1,3,5,10,13,16,21-heptaazacyclopentacos-3-en-19-yl)amino]-4-oxobutyl}-2,6-dimethyl-4-[(*E*)-2-(2,3,6,7-tetrahydro-1*H*,5*H*-pyrido[3,2,1-ij]quinolin-9-yl)vinyl]pyridin-1-ium Bis(hydrotrifluoroacetate) Trifluoroacetate (**18**)

Peptide **6** (1.63 mg, 0.97 μmol) was dissolved in anhydrous DMF (150 μL) followed by the addition of DIPEA (1.1 μL, 6.31 μmol), the addition of a solution of **14** (0.47 mg, 1.16 μmol) in DMF (15 μL) and shaking of the mixture at rt in the dark for 2 h. 100 μL of 1% aqueous TFA were added, and the mixture was subjected to preparative HPLC (system 1, gradient: 0–20 min: 0.1% aq TFA/acetonitrile 90:10–50:50, *t*_R_ = 14 min) to yield **18** as a red fluffy solid (0.61 mg, 34%). HRMS (ESI): *m*/*z* [M^+^ + 3H]^4+^ calcd. for [C_76_H_113_N_21_O_12_]^4+^ 377.9714, found: 377.9727. RP-HPLC (220 nm): 97% (*t*_R_ = 13.1 min, *k* = 16.2). C_76_H_110_N_21_O_12_^+^·C_6_H_2_F_9_O_6_^–^ (1508.86 + 341.06).

##### 1-{3-[4-(4-{3-[(*E*)-Amino({3-[(9*S*,12*S*,15*S*,*E*)-4-amino-9-({(*S*)-1-[((*S*)-1-{[(*S*)-1-amino-3-(4-hydroxyphenyl)-1-oxopropan-2-yl]amino}-5-guanidino-1-oxopentan-2-yl)amino]-4-methyl-1-oxopentan-2-yl}carbamoyl)-12-(4-hydroxybenzyl)-2,11,14,17,20-pentaoxo-1,3,5,10,13,16,21-heptaazacyclopentacos-3-en-15-yl]propyl}amino)methylene]ureido}butyl)-1*H*-1,2,3-triazol-1-yl]propyl}-4-{(1*E*,3*E*)-4-[4-(dimethylamino)phenyl]buta-1,3-dien-1-yl}-2,6-dimethylpyridin-1-ium Bis(hydrotrifluoroacetate) Trifluoroacetate (**19**)

Peptide **11** (0.84 mg, 0.53 μmol) was dissolved in H_2_O (190 μL). A solution of copper(II)sulfate pentahydrate (0.2 mg, 0.64 μmol) in H_2_O (30 μL), a solution of sodium ascorbate (0.36 mg, 1.59 μmol) in H_2_O (30 μL) and a solution of **15** (0.25 mg, 0.53 μmol) in NMP (70 μL) were added followed by the addition of 180 μL of NMP to adjust the ratio of the solvents (H_2_O/NMP) to 1:1 v/v (final volume: ca. 500 μL). The mixture was shaken at rt in the dark for 2 h. 100 μL of 1% aqueous TFA were added, and the mixture was subjected to preparative HPLC (system 1, gradient: 0–20 min: 0.1% aq TFA/acetonitrile 80:20–50:50, *t*_R_ = 9 min) to yield **19** as a red fluffy solid (0.29 mg, 27%). HRMS (ESI): *m*/*z* [M^+^ + 3H]^4+^ calcd. for [C_80_H_120_N_24_O_12_]^4+^ 402.2374, found: 402.2380. RP-HPLC (220 nm): 95% (*t*_R_ = 13.7 min, *k* = 17.0). C_80_H_117_N_24_O_12_^+^·C_6_H_2_F_9_O_6_^–^ (1606.97 + 341.06).

#### Chemical Stability

The chemical stability of the fluorescent ligands **16, 17**, and **18** was investigated in PBS (adjusted to pH 7.4) at 22 °C. The incubation was started by the addition of 7.5 μL of a 2 mM stock solution (solvent: DMSO/H_2_O 1:1 v/v) to 142.5 μL of PBS (in the case of compound **18** supplemented with 10% DMSO due to solubility reasons) to yield a concentration of 100 μM. After time periods of 0, 6, and 48 h, an aliquot (25 μL) was withdrawn and added to 25 μL of acetonitrile/0.04% aq. TFA 1:9 v/v to obtain a peptide solution with a concentration of 50 μM. 20 μL of this solution were subjected to analytical RP-HPLC analysis using the same system and conditions as described under Analytical HPLC, but applying a different linear gradient: 0–14 min: A/B 90:10–45:55, 14–15 min: 45:55–5:95, 15–19 min: 5:95 (isocratic). A 1:1 mixture of PBS and acetonitrile/0.04% aq. TFA 1:9 v/v (20 μL) was analyzed to obtain the blank chromatogram.

#### Fluorescence Excitation and Emission Spectra

Excitation and emission spectra were recorded with a Cary Eclipse spectrofluorimeter (Varian, Mulgrave, Victoria, Australia) using polystyrene cuvettes (10 mm × 10 mm, reference 1961, Kartell, Noviglio, Italy). Sample solutions (2 mL) were prepared in the cuvettes. Spectra in PBS (pH 7.4) and PBS supplemented with 1% BSA were obtained for all fluorescent ligands (**16**–**19**) using the following concentrations: 6 μM for **19**, 5 μM for **18** (in PBS), and 1 μM for **16**, **17**, and **18** (in PBS with 1% BSA). Excitation spectra (cf. Figure S7) were recorded with spectral bandwidths of 5 nm (excitation slit) and 10 nm (emission slit). The spectral bandwidth applied for the emission spectra was 10 nm (excitation slit) and 5 nm (emission slit). Net spectra were obtained by subtracting the respective reference spectrum of a vehicle sample.

#### Determination of Fluorescence Quantum Yields

The fluorescence quantum yield of compound **19** was determined following a reported procedure using the aforementioned Cary Eclipse spectrofluorimeter for the measurement of emission spectra and a Lambda 650 UV/vis spectrophotometer (PerkinElmer, Waltham, MA) for the measurement of absorption spectra.^41^ The concentration of **19** was 6 μM and cresyl violet perchlorate (Acros Organics, Geel, Belgium) was used as reference compound (concentration: 2 μM). The excitation wavelength was set close to the absorption maximum (500 nm for **19** in PBS, 515 nm for **19** in PBS with 1% BSA). Emission spectra of cresyl violet perchlorate in EtOH were recorded at an excitation wavelength of 575 nm. Sample solutions (2 mL) were prepared in polystyrene cuvettes (10 mm × 10 mm), immediately followed by measurement of the emission spectra, transfer of the solutions into acryl cuvettes (10 mm × 10 mm, reference 67.755, Sarstedt), and measurement of the absorption spectra. Emission spectra were recorded for the spectral bandwidth settings (excitation/emission) of 10/5 and 10/10 nm. The obtained quantum yields were averaged.

The fluorescence quantum yield of compounds **16**–**18** was determined via an absolute method using an Ulbricht sphere with an inaccuracy of ca. 3% (Hamamatsu C9920–02 system equipped with a Spectralon integrating sphere) at room temperature (23 ± 1 °C). The optical density at the excitation wavelength of the sample was <0.1 (optical path length: 1 cm). Samples were measured in a 10 mm × 10 mm quartz cuvette.

#### Cell Culture

Cells were cultured in T75 or T175 tissue culture flasks (Sarstedt, Nümbrecht, Germany) in a humidified atmosphere (95% air, 5% CO_2_) at 37 °C. SK-N-MC neuroblastoma cells (obtained from the American Type Culture Collection, ATCC HTB-10) were maintained in EMEM (Sigma) supplemented with 5% FBS. CHO-hY_2_R cells (obtained from PerkinElmer, Rodgau, Germany) were cultured in Ham’s F-12 supplemented with 5% FBS and G418 (400 μg/mL). CHO-hY_4_-Gq_i5_-mtAEQ cells^25^ were cultured in HAM’s F-12 (Sigma) supplemented with 10% FBS, hygromycin (400 μg/mL), zeocin (250 μg/mL) and G418 (400 μg/mL). HEC-1B-hY_5_R cells^59^ were maintained in EMEM supplemented with 5% FBS and G418 (400 μg/mL). HEK293T-NlucN-mG_si_/Y_4_R-NlucC cells^37^ were cultured in DMEM (Sigma) supplemented with 10% FBS, puromycin (1 μg/mL), and G418 (600 μg/mL). HEK293T-CAMYEN-hY_4_R cells^31^ were cultured in DMEM supplemented with 10% FBS, zeocin (100 μg/mL), and G418 (600 μg/mL). Human SK-OV-3 ovarian adenocarcinoma cells (obtained from the American Type Culture Collection, ATCC HTB-77) were maintained in McCoy’s 5A Medium (Corning) supplemented with 10% FBS, 2 mM l-glutamine, 100 U/mL penicillin, and 100 μg/mL streptomycin.

#### Molecular Cloning

All enzymes and reagents used for cloning were obtained from New England Biolabs (Frankfurt am Main, Germany) unless stated otherwise. Plasmids were generated using standard restriction cloning techniques, in detail: the plasmid pcDNA3.1-Nluc-hY_4_R encoding the N-terminally Nanoluciferase-tagged human NPY Y_4_ receptor, was cloned by exchanging the receptor-encoding sequence in Nluc-hH_4_R (in pcDNA3.1)^60^ with the sequence of the hY_4_R. Therefore, the nucleotide sequence for the hY_4_R was first amplified from pcDNA3.1-hY_4_R (obtained from Missouri cDNA Research Center, Rolla, MO) via polymerase chain reaction (PCR, using Phusion High Fidelity Polymerase according to the manufacturer’s instructions) attaching flanking *BamH*I and *Apa*I to the 5′ and 3′ ends, respectively. Sequences of the used primers were as follows: forward, CTGAGGATCCATGAACACCTCTCACCTC; reverse, GACTGGGCCCGAAATGGGATTGGACCTGCCAC. After a PCR purification step using the FastGene Gel/PCR Extraction Kit (Nippon Genetics, Düren, Germany), both the purified PCR product and the target plasmid Nluc-hH_4_R were subjected to a restriction digest using *BamH*I and *Apa*I restriction enzymes overnight at 37 °C. Restriction digests were gel purified, ligated using T4 DNA ligase at room temperature for 1 h, and transformed into Top10F′ *Escherichia coli* (made competent in house).

For the plasmid pcDNA3.1-hY_4_R-Nluc(intraECL2), which encodes the Y_4_R with Nluc in the second extracellular loop (inserted via short glycine-serine linkers after the leucine in position 195 (L195)), pcDNA3.1-hY_4_R served as a vector backbone and pcDNA3.1-Nluc-Y_1_R(Y192)^51^ was used to extract the Nluc insert. First, pcDNA3.1-hY_4_R was linearized after L195 via PCR adding flanking *BamH*I (5′ end of the linearized vector) and *Sac*I (3′ end of the linearized vector; note: the *Sac*I restriction site was incorporated into a -SGGGGSS- linker sequence) restriction sites using the aforementioned Phusion polymerase. Sequences of the used primers were as follows: forward: GATCGGATCCGCAGATAAGGTGGTCTGTAC; reverse: GATCAGAGCTCCCTCCACCGCCACTCAGGAACTCCAGAGCCTTG. Next steps were performed as described above: After a PCR cleanup, the purified PCR product and the pcDNA3.1-Nluc-Y_1_R(Y192) (used to excise the Nluc insert) were subjected to a restriction digest using *BamH*I and *Sac*I restriction enzymes at 37 °C overnight. Restriction digests were gel purified, ligated, and transformed into competent *E. coli*, as described above. Plasmids were extracted from bacterial overnight cultures (Miniprep Kit, Nippon Genetics, Düren, Germany) and all sequences were verified by Sanger Sequencing (Eurofins Genomics, Ebersberg, Germany).

Plasmids used to prepare MultiBac(Mam) baculoviruses,^61^ were generated according to the ACEMBL technology concept.^62^ Acceptor and donor plasmids, used to prepare multicomponent DNA constructs, were joined by the Cre-LoxP fusion reaction. The hY_4_R-Nluc sequence from pcDNA3.1-hY_4_R-NlucC^37^ (note: NlucC, fused to the C-terminus of Y_4_R, stands for the C-terminal fragment of Nluc consisting of 11 amino acids) was recloned into the pIMACE-CMVintP10 vector. This vector was obtained by replacing the insect polyhedrin promoter in pACEBac1 (Geneva Biotech, Pregny-Chambésy, Switzerland) with dual-host (insect/mammalian) CMVintP10 promotor taken from pSPL-IM vector (Geneva Biotech). For this purpose, pACEBac1 and pSPL-IM were both digested with the FastDigest restriction enzymes *Bsu15*I and *XmaJ*I (Thermo Fisher Scientific) according to the manufacturer’s instructions. Restriction digests were gel purified using the FavorPrep Gel/PCR Purification Kit (Favorgen, Vienna, Austria), ligated by T4 DNA ligase (Thermo Fisher Scientific) at 22 °C for 1 h, and transformed into DH5α *E. coli* (made competent in house). After selection with gentamycin (MP Biomedicals, Eschwege, Germany), plasmids were extracted from overnight bacterial cultures (FavorPrep Plasmid DNA Extraction Kit). The resulting pIMACE-CMVintP10 was digested with *Hind*III and *Sca*I and pcDNA3.1-hY_4_R-Nluc was digested with *Hind*III and *Mss*I to obtain the acceptor plasmid pIMACE-CMVintP10-hY_4_R-Nluc after gel purification and ligation. Depending on the targets of further use (mammalian, insect, or insect with mammalian-like glycosylation expression system), the acceptor plasmid pIMACE-CMVintP10-hY_4_R-Nluc was subjected to different types of recombination.

To obtain baculoviruses used for hY_4_R transduction into human SK-OV-3 ovarian adenocarcinoma cells, the acceptor plasmid pIMACE-CMVintP10-hY_4_R-Nluc was recombined with the donor plasmid pIDC-VSVG using Cre recombinase (New England Biolabs) and the product was propagated in pirHC *E. coli* strain (Geneva Biotech) under selection pressure of gentamycin and chloramphenicol (Sigma). Pseudotyping of baculovirions with vesicular stomatitis virus G-protein (VSVG) has been reported to enhance the transduction efficiency of mammalian cells by these baculoviruses. The donor plasmid pIDC-VSVG was obtained by inserting VSVG from the pUCDM-VSVG vector (Geneva Biotech) into the pIDC vector (Geneva Biotech): pIDC was digested with *Rru*I and *XmaJ*I, and pUCDM-VSVG was digested with *Mss*I and *XmaJ*I. After the cre-recombination step, the resulting multicomponent construct pIMACE-CMVintP10-hY_4_R-Nluc × pIDC-VSVG was subjected to restriction analysis and used for transposition into baculovirus genomes presented as bacterial artificial chromosomes in *E. coli* cells (DH10MultiBac, Geneva Biotech) to generate baculovirus Y_4_R^VSVG^.

To create Y_4_R baculoviruses for the expression of hY_4_R in Sf9 insect cells, pIMACE-CMVintP10-hY_4_R-Nluc was directly used for transposition into DH10MultiBac. To mimic the mammalian-like pattern of protein glycosylation in Sf9 cells, another type of baculovirus, encoding for Y_4_R^SwBac^, was prepared. For this purpose, the acceptor plasmid pIMACE-CMVintP10-hY_4_R-Nluc was recombined with the donor plasmid pIDC-SweetBac. This plasmid was obtained by recloning the SweetBac module^49^ from pUDCM-GntII-GalT plasmid (Geneva Biotech) into the pIDC backbone: pUDCM-GntII-GalT was digested with *Mss*I and *XmaJ*I, and pIDC was digested with *Rru*I and *XmaJ*I. As before, the generated multicomponent construct pIMACE-CMVintP10-hY_4_R-Nluc × pIDC-SweetBac was restriction analyzed and used for transposition into DH10MultiBac.

#### Generation of the Stable HEK293T-Nluc-hY_4_R and HEK293T-hY_4_R-Nluc(intraECL2) Cell Lines

HEK293T cells were seeded into a 6-well dish in DMEM supplemented with 10% FBS at 300,000 cells/mL. The following day, cells were transfected with 2 μg of pcDNA3.1-Nluc-hY_4_R or pcDNA3.1-hY_4_R-NLuc(intraL195) using the XtremeGene HP transfection reagent (Merck, Darmstadt, Germany) according to the manufacturer’s protocol. Cells were passaged after 48 h into a T175 culture flask and selected for 2 weeks at a high G418 concentration (1000 μg/mL). The culture medium was changed three times in this time period and the cells were regularly monitored for colonial growth. Starting with the observation of the latter, cells were cultured in DMEM supplemented with 10% FBS and 600 μg/mL G418 for maintained selection pressure.

#### Buffers and Media Used for Binding and Functional Assays

Buffer I (used for radiochemical (Y_2_R, Y_4_R) and flow cytometric (Y_4_R) binding experiments): a hypotonic sodium-free HEPES buffer (25 mM HEPES, 2.5 mM CaCl_2_, 1 mM MgCl_2_, adjusted to pH 7.4 using 25% ammonia in water) supplemented with 1% BSA (Serva, Heidelberg, Germany) and 0.1 mg/mL bacitracin (Serva).

Buffer II (used for binding experiments at the Y_1_R and Y_5_R): an isotonic sodium-containing HEPES buffer (10 mM HEPES, 150 mM NaCl, 25 mM NaHCO_3_, 2.5 mM CaCl_2_, 5 mM KCl, pH 7.4) supplemented with 1% BSA.

Buffer III (used for the Ca^2+^ aequorin assay): an isotonic sodium-containing HEPES buffer (25 mM HEPES, 120 mM NaCl, 1.5 mM CaCl_2_, 1 mM MgCl_2_, 5 mM KCl, 10 mM d-glucose, pH 7.4).

DPBS (used for flow cytometric and fluorescence anisotropy-based Y_4_R binding assays): Dulbecco’s phosphate-buffered saline with calcium and magnesium (1.8 mM CaCl_2_, 2.68 mM KCl, 1.47 mM KH_2_PO_4_, 3.98 mM MgSO_4_, 137 mM NaCl, 8.06 mM Na_2_HPO_4_, pH 7.4) supplemented with 1% BSA and 0.1 mg/mL bacitracin (flow cytometry) or with 0.1% Pluronic F-127 and cOmplete EDTA-free Protease Inhibitor Cocktail (fluorescence anisotropy).

L15-HEPES (used for miniG_si_ recruitment, cAMP CAMYEN and NanoBRET binding assays): Leibovitz’s L-15 medium (140 mM NaCl, 1.3 mM CaCl_2_, 1 mM MgCl_2_, various amino acids and vitamins) (Fisher Scientific, Schwerte, Germany) supplemented with 10 mM HEPES and additionally with 5% FBS (miniG_si_ recruitment and cAMP CAMYEN assays) or 1.5% BSA (NanoBRET). Before the addition of FBS and BSA, the pH was adjusted to 7.4.

#### Radioligand Binding Assays

All radioligand binding assays were performed at intact Y receptor expressing cells at 23 ± 2 °C. The radiochemical competition binding assays, used to study Y_1_, Y_2_, Y_4_, and Y_5_ receptor binding of peptide **11**, and fluorescent ligands **16**–**19**, were recently validated by the determination of binding affinities of the reference ligands pNPY (Y_1_R, Y_2_R, and Y_5_R) and hPP (Y_4_R), which were in good agreement with previously reported binding affinities (for determined and reference p*K*_i_ or *K*_i_ values of pNPY (Y_1_R, Y_2_R, Y_5_R), see Konieczny et al.;^30^ for determined and reference p*K*_i_ values of hPP (hY_4_R), see Gleixner et al.^31^).

### Y_1_R Binding

Competition binding assays at Y_1_R-expressing SK-N-MC neuroblastoma cells were performed as previously described using [^3^H]UR-MK299 as radioligand (concentration: 0.15 nM).^33^ Due to low radioligand displacement, no curve fitting was performed for the investigated compounds **11** and **17**–**19** (studied at concentrations up to 10 μM for **11** and 3 μM for **17**–**19**). For compound **16**, binding data (dpm) were normalized (100% = bound radioligand in the absence of competitor; 0% = unspecific binding), plotted as % over log(concentration of competitor, *M*), and analyzed by a four-parameter logistic fit (log(inhibitor) vs response–variable slope; GraphPad Prism 5, GraphPad Software, San Diego, CA) to obtain pIC_50_ and IC_50_ values, which were converted to p*K*_i_ and *K*_i_ values according to the Cheng–Prusoff equation^63^ (logarithmic form in the case of p*K*_i_ values).

### Y_2_R Binding

Competition binding assays at CHO-hY_2_R cells were performed as previously described using [^3^H]propionyl-pNPY (*K*_d_ = 0.14 nM, concentration: 0.5 nM) as radioligand.^34^ Due to low radioligand displacement, no curve fitting was performed for the investigated compounds **11** and **16**–**19** (studied at concentrations up to 10 μM for **11** and 3 μM for **16**–**19**).

### Y_4_R Binding

Competition binding experiments at CHO-hY_4_R-G_qi5_-mtAEQ cells using [^3^H]UR-KK200 as radioligand (*K*_d_ = 0.67 nM, concentration: 1.0 nM)^35^ were performed as previously described.^30^ Binding data (dpm) were normalized (100% = bound radioligand in the absence of competitor; 0% = unspecific binding), plotted as % over log(concentration of competitor, *M*), and analyzed by a four-parameter logistic fit (log(inhibitor) vs response–variable slope; GraphPad Prism 5) to obtain pIC_50_ and IC_50_ values, which were converted to p*K*_i_ and *K*_i_ values according to the Cheng–Prusoff equation^63^ (logarithmic form in the case of p*K*_i_ values).

### Y_5_R Binding

Competition binding studies at HEC-1B-hY_5_R cells using [^3^H]propionyl-pNPY (*K*_d_ = 11 nM,^26^ concentration: 5 nM) as radioligand were performed as previously reported.^35^ Due to low radioligand displacement, no curve fitting was performed for the investigated compounds **11**, **17**, and **19** (studied at concentrations up to 10 μM for **11**, 3 μM for **17**, and 1 μM for **19**). For compounds **16** and **18**, binding data (dpm) were normalized (100% = bound radioligand in the absence of competitor; 0% = unspecific binding), plotted as % over log(concentration of competitor, *M*), and analyzed by a four-parameter logistic fit (log(inhibitor) vs response–variable slope; GraphPad Prism 5) to obtain pIC_50_ and IC_50_ values, which were converted to p*K*_i_ and *K*_i_ values according to the Cheng–Prusoff equation^63^ (logarithmic form in the case of p*K*_i_ values).

#### Functional Y_4_R Assays

All functional assays were performed in agonist mode as all studied compounds exhibit Y_4_R agonistic activity.

### Y_4_R Ca^2+^ Aequorin Assay

The assay was performed with live CHO-hY_4_R-G_qi5_-mtAEQ cells as previously described (used buffer: buffer III). Measurements were carried out on a Tecan Infinite Lumi plate reader. Data analysis was performed as reported using GraphPad Prism 5 for the calculation of the area under the curve. Fractional luminescences, obtained by dividing the area of the initially occurring agonist-induced peak by the total area (sum of the areas of the agonist-induced peak and the subsequent lysis-induced peak), were normalized (100% = fractional luminescence obtained from 1 μM hPP, 0% = fractional luminescence obtained by vehicle addition (basal effect), GraphPad Prism 5). The normalized responses were plotted against log(concentration of agonist, *M*) and the data were fitted according to a four-parameter logistic equation (log(agonist) vs response–variable slope, GraphPad Prism 5) to obtain pEC_50_ values, which were converted to EC_50_ values. Efficacies *E*_max_ correspond to the upper plateaus of the normalized concentration–response curves.

### Y_4_R miniG_si_ Recruitment Assay

The assay was performed with live HEK293T-NlucN-mG_si_/Y_4_R-NlucC cells using a reported protocol with a minor modification:^37^ on the day of the experiment, cells were not washed with FBS-free medium, i.e., the assay was performed in L15-HEPES supplemented with 5% FBS. Furimazine was used as nanoluciferase substrate. The original stock was diluted 1:1000 in L15-HEPES to obtain the feed solution for the assay. Data analysis was performed based on the areas under the curves (time window: 45 min starting with the agonist addition) using GraphPad Prism 5. Normalized responses (100% = response induced by 1 μM hPP, 0% = vehicle control) were plotted against log(concentration of agonist, *M*) and the data were fitted according to a four-parameter logistic equation (log(agonist) vs response–variable slope, GraphPad Prism 5) yielding pEC_50_ values, which were converted to EC_50_ values. Efficacies *E*_max_ correspond to the upper plateaus of the normalized concentration–response curves.

### Y_4_R cAMP CAMYEN Assay

The assay was performed with live HEK293T-CAMYEN-hY_4_R cells applying a reported protocol.^31^ Experiments were performed in triplicate. Data processing was performed with GraphPad Prism 9.0 as previously described.^31^

#### Flow Cytometric Y_4_R Binding Assays

Flow cytometry-based Y_4_R binding studies with the fluorescent ligands **16, 17**, and **18** were performed at intact CHO-hY_4_R-G_qi5_-mtAEQ cells with a FACSCantoII flow cytometer (Becton Dickinson), equipped with an argon laser (488 nm), a red diode laser (640 nm) and a BD High Throughput Sampler (HTS unit) for microtiter plates. The latter was used for injection of the samples of saturation and competition binding experiments. At least three individual experiments were performed in duplicate (kinetic experiments) or triplicate (saturation and competition binding). The following gain settings for forward and sideward scatter were applied throughout: FSC: 0 V, SSC: 252 V. Fluorescence was recorded using the following settings: compound **16**, excitation at 488 nm, emission at 585 ± 21 nm (PE channel), gain: 470–500 V; compound **17**, excitation at 640 nm, emission at 660 ± 10 nm (APC channel), gain: 450 V; compound **18**, excitation at 488 nm, emission at >670 nm (PerCP-Cy5.5 channel), gain: 470–500 V, propidium (cell viability studies), excitation at 488 nm, emission at 585 ± 21 nm (PE channel), gain: 330 V. For measurements requiring the use of classical injection tubes (kinetic experiments), the medium flow rate (60 mL/min) was used. Measurements were stopped after counting of 2000–10000 gated events.

2 to 3 days prior to the experiment, cells were seeded in T75 or T175 culture flasks. On the day of the experiment, cells were detached by trypsinization, suspended in culture medium, and centrifuged. The cell pellet was resuspended in buffer I (binding studies with **16**) or DPBS (binding studies with **16**–**18**), and the cell density was adjusted to 1.5 × 10^5^ (saturation binding) or 2 × 10^5^ cells/mL (kinetic and competition binding experiments). All receptor ligands were added to the cell suspension as 100-fold concentrated (compared to the final concentration) solutions in DMSO/H_2_O 1:1 v/v (fluorescent ligands, all experiments; nonlabeled ligands, competition binding studies) or in 10 mM aq. HCl (nonlabeled ligands, saturation, and kinetic experiments).

For saturation binding experiments, the wells of a polypropylene round-bottom 96-well plate (Brand, Wertheim) were prefilled with 200 μL of cell suspension followed by the addition of 2 μL of 10 mM aq. HCl (samples for total binding) or 2 μL of a 100 μM solution of hPP (samples for unspecific binding). The final concentration of hPP was 1 μM. Incubation was started by adding 2 μL of a 100-fold concentrated fluorescent ligand solution followed by gentle shaking in the dark at 22 ± 2 °C for 2 h and subsequent measurement using the HTS unit (sample volume: 45 μL, injection speed: 1.5 μL/s). For the determination of autofluorescence, three ligand-free samples were prepared by the addition of 2 μL of 10 mM aq. HCl and 2 μL of DMSO/H_2_O 1:1 v/v to the cell suspension, followed by incubation and measurement.

For association experiments, polypropylene tubes (usable as injection tubes for the FACSContoII flow cytometer) were prefilled with 2000 μL of cell suspension and 20 μL of 10 mM aq. HCl (samples for total binding) or 20 μL of a 100 μM solution of hPP (samples for unspecific binding) were added. The association was started by the addition of 20 μL of a 30 nM (buffer I) or 70 nM (DPBS) solution of **16** (final concentrations: 0.3 and 0.7 nM, respectively), 20 μL of a 100 nM solution of **17** in DPBS (final concentration: 1.0 nM), or 20 μL of a 50 nM solution of **18** in DPBS (final concentration: 0.5 nM) to the cell suspension, followed by short mixing and gentle shaking under protection from light at 22 ± 2 °C. After different periods of time (0.5–120 min for all studied fluorescent ligands), sample aliquots were measured by placing the tube in the injection port of the cytometer.

For dissociation experiments, polypropylene tubes (usable as injection tubes for the FACSContoII flow cytometer) were prefilled with 2000 μL of cell suspension followed by the addition of 20 μL of 10 mM aq. HCl (samples for total binding) or 20 μL of a 100 μM solution of hPP (samples for unspecific binding). Preincubation was started by the addition of 20 μL of a 150 nM (buffer I) or 350 nM (DPBS) solution of **16** (final concentrations: 1.5 and 3.5 nM, respectively), 20 μL of a 500 nM solution of **17** in DPBS (final concentration: 5 nM) or a 250 nM solution of **18** in DPBS (final concentration: 2.5 nM). The samples were gently shaken in the dark at 22 ± 2 °C for 2 h. The dissociation was initiated by the addition of 20 μL of a solution of hPP and **5** (final concentrations: 1000-fold (hPP) and 100-fold (**5**) over the final concentration of the respective fluorescent ligand) prepared in the buffer used for the respective experiment (note: a combination of hPP and **5** was used to prevent rebinding of the labeled ligand more effectively). After different periods of time (**16** in buffer I: 1–360 min, **16** in DPBS: 1–300 min, **17** in DPBS: 1–200 min, **18** in DPBS: 1–300 min), sample aliquots were measured by placing the tube in the injection port of the cytometer.

For competition binding experiments with **16**, the wells of a polypropylene round-bottom 96-well plate were prefilled with cell suspension (200 μL) followed by the addition of 2 μL of a 100-fold concentrated solution of the competing (nonlabeled) ligand and gentle shaking in the dark for 5 min. 2 μL of the fluorescent ligand solution (100-fold concentrated) were added and shaking in the dark at 22 ± 2 °C was continued for 2 h. The final concentrations of **16** were 0.5 nM (buffer I) and 1 nM (DPBS). For the determination of unspecific binding and total binding in the absence of the studied competitor, 2 μL of a 100 μM solution of hPP (final concentration: 1 μM) and 2 μL of DMSO/water 1:1 v/v, respectively, were initially added instead of the competitor solution, followed by the addition of the fluorescent ligand after the preincubation period and further processing as described above.

Specific binding data were obtained by subtracting unspecific binding from total binding. Unspecific binding data shown in Figure 4 are autofluorescence corrected. Specific binding data from saturation binding experiments were analyzed by an equation describing a hyperbolic isotherm (binding-saturation: one site-specific binding, GraphPad Prism 5) yielding *K*_d_ values, which were converted to p*K*_d_ values. Specific binding data from association experiments were analyzed by a three-parameter equation describing a monophasic exponential rise to a maximum (*Y*_0_ constrained to zero), yielding *k*_obs(mono)_, and by a five-parameter equation describing a biphasic exponential rise to a maximum (*Y*_0_ constrained to zero) yielding *k*_obs(bi,fast)_ and *k*_obs(bi,slow)_ (GraphPad Prism 5). Specific binding data from dissociation experiments were analyzed by a two-parameter equation describing a monophasic exponential decline (GraphPad Prism 5) yielding *k*_off_. Association rate constants *k*_on(mono)_, *k*_on(bi,fast)_, and *k*_on(bi,slow)_ were calculated from the observed association rate constants *k*_obs(mono)_, *k*_obs(bi,fast)_, and *k*_obs(bi,slow)_ (mean values), the mean values of the corresponding dissociation rate constants *k*_off_ (cf. Table 4), and the fluorescent ligand concentrations used for the association experiments (see above) according to the equation *k*_on_ = (*k*_obs_ – *k*_off_)/[fluorescent ligand]. The kinetically derived dissociation constants *K*_d_(kin) were calculated from *k*_on(mono)_ and the mean value of the dissociation rate constants *k*_off_ (cf. Table 4) according to the equation *K*_d(kin)_ = *k*_off_/*k*_on_.

Total binding data from competition binding experiments were plotted as fluorescence intensity over log(competitor concentration, *M*) and analyzed by a four-parameter logistic equation [log(inhibitor) vs response-variable slope, GraphPad Prism 5] to obtain the TOP value (upper curve plateau), which was used for data normalization (100% = TOP value, 0% = average signal obtained from unspecific binding). The normalized data (%) were plotted over log(competitor concentration, *M*) and analyzed by a four-parameter logistic equation to obtain pIC_50_ values, which were converted to p*K*_i_ values according to the Cheng–Prusoff equation (logarithmic form) using the following *K*_d_ values of **16**: buffer I, *K*_d_ = 0.30 nM; DPBS, *K*_d_ = 0.70 nM (mean *K*_d_ values from three individual saturation binding experiments).

For cell viability studies, cell suspensions were prepared as for fluorescent ligand binding experiments, but with a higher cell density (3 × 10^5^ cells/mL for buffer I and DPBS). The cell suspensions were shaken in 50 mL polypropylene vessels (VWR International, Radnor, PA) at 22 °C. For measurements, 250 μL aliquots were transferred into polystyrene measurement tubes. 2.5 μL of an aqueous solution of propidium iodide (200 μg/mL; final concentration: 2 μg/mL) were added followed by a short incubation period (1 min) and measurement. Measurements were stopped after counting of 20,000 gated events.

#### Fluorescence Anisotropy Y_4_R Binding Assays

Fluorescence anisotropy-based Y_4_R binding studies with fluorescent ligand **16** were performed at hY_4_R displaying budded baculovirus particles (termed BBVs below), which were obtained from Sf9 insect cells following the procedure described for the preparation of Y_1_R displaying BBVs.^40^ Two types of MultiBac baculoviruses were used: one type encodes only for hY_4_R resulting in BBVs displaying Y_4_R devoid of a mammalian-like glycosylation (herein termed Y_4_R^nonglyco^) and the second type additionally encodes for enzymes providing a mammalian-like glycosylation in Sf9 cells (herein termed Y_4_R^SwBac^; see also Section Molecular Cloning). The obtained viruses were collected by centrifugation at 1600*g* for 10 min, and the virus titer was determined with an image-based cell-size estimation assay as described elsewhere.^64^ The viruses were amplified using a multiplicity of infection (MOI) between 0.01 and 0.1 until a high-titer baculovirus stock (virus concentration of at least 1.0 × 10^8^ infectious viral particles/mL) was acquired. To produce BBV preparations with a high receptor density, Sf9 cells with a density of 1.9 × 10^6^ cells/mL were infected with an MOI of 3 and the BBVs were collected by centrifugation 63 h after the infection, when the cell viability was <30%. The BBVs were concentrated 40-fold by centrifugation at 48,000*g* at 4 °C for 40 min, washed with ice-cold DPBS, resuspended, and homogenized using a 0.3 mm diameter needle (Sterican, B. Braun, Melsungen, Germany). Aliquots of the BBV preparations were stored at −90 °C until use.

FA measurements were carried out using a Synergy NEO multimode plate reader (BioTek, Winooski, VT) and black, half-area, flat-bottom polystyrene NBS (nonbinding surface) 96-well plates (Corning, Corning, NY, product no. 3993). Polarizing excitation (530 ± 15 nm) and dual emission (590 ± 17.5 nm) filters with a dichroic mirror were used, allowing the simultaneous detection of parallelly and perpendicularly polarized fluorescence emission. All measurements were performed at 27 °C. Saturation binding experiments, association and dissociation experiments, and competition binding experiments were performed in duplicate, following a previously reported protocol with minor modifications.^65^ All ligand dilutions were prepared in DPBS at 4- or 5-fold concentrations compared to the final concentration in the assay. The final total volume per well was 100 μL. Frozen preparations of hY_4_R displaying BBVs were thawed and thoroughly resuspended using a 1 mL syringe (Norm-Ject-F, Braun Melsungen) with a 0.3 mm diameter needle (Sterican, B. Braun).

For equilibrium binding studies (saturation binding experiments), used for the determination of equilibrium *K*_d_ values and the Y_4_R concentration in the BBV preparations, 25 μL of a 4-fold concentrated solution of **16** (used at final concentrations of 0.5 and 2 nM) and 25 μL of DPBS were premixed in the 96-well plate followed by the addition of 50 μL of the BBV suspension (used in 6 or 12 different dilutions) to initiate total binding. To determine unspecific binding, the procedure was the same, but instead of neat binding buffer, 25 μL of a 4 μM solution of hPP (final concentration: 1 μM) were added. For blank measurements, 50 μL of the BBV suspension were added to 50 μL of DPBS. Experiments were performed with 6 or 12 different BBV dilutions. Measurements were started immediately after the addition of BBV suspension and were stopped after 90 min.

For association experiments, 20 μL of a 5-fold concentrated solution of **16** (used at final concentrations of 0.5 and 2 nM) and 20 μL of DPBS were premixed in the 96-well plate followed by the addition of 60 μL of the BBV suspension (used in six different dilutions) to initiate total binding. To determine unspecific binding, the procedure was the same, but instead of neat binding buffer, 20 μL of a 5 μM solution of hPP (final concentration: 1 μM) were added. For blank measurements, 60 μL of the BBV suspension were added to 40 μL of binding buffer. Note that due to the very fast association of **16** with the Y_4_R, measurements were not performed simultaneously for all BBV dilutions. Instead, to enable short time intervals between the readout cycles, individual measurements (for 10 min) were performed for all BBV concentrations. For each combination of receptor and ligand concentration, binding equilibrium was reached within this time period.

For dissociation experiments, the preincubation was set up as the association experiments (final concentrations of **16** of 0.5 or 2 nM, same BBV dilutions). Samples were incubated for 1 h, followed by starting the dissociation by the addition of 2 μL of a solution containing 50 μM hPP and 5 μM **5** (final concentrations: 1 μM and 100 nM, respectively) prepared in DPBS, and the FA signal was recorded for 2 h.

Note that in principle, association, equilibrium binding, and dissociation experiments can be performed simultaneously in one plate. However, as mentioned above, association experiments, requiring a higher data sampling rate than equilibrium binding and dissociation experiments, had to be performed consecutively for the various combinations of BBV and ligand concentrations. In contrast, in the case of equilibrium binding and dissociation experiments, all combinations were measured simultaneously.

For competition binding experiments (only performed with Y_4_R^SwBac^), 20 μL of a 5-fold concentrated solution of the competitive ligand (used at varying concentrations) and 20 μL of a 5-fold concentrated solution of **16** (final concentration: 0.5 nM) were premixed in the 96-well plate followed by the addition of 60 μL of the BBV suspension (final concentration of Y_4_R^SwBac^: 0.13 nM) to initiate total binding. To determine total binding in the absence of competitor and unspecific binding, the same procedure was used, but instead of the addition of competitive ligand, 20 μL of neat binding buffer and 20 μL of a 5 μM solution of hPP (final concentration: 1 μM), respectively, were added. FA signals were measured for 1.5 h.

Data processing for FA binding assays was assisted by the software Aparecium 2.0.20 developed in house (http://www.gpcr.ut.ee/software.html). Prior to the calculation of FA using eq 1,^19^ the measured parallel [*I*_∥_(*t*)] and perpendicular [*I*_⊥_(*t*)] fluorescence intensities were blank-corrected on the basis of the values obtained from wells containing BBVs but no receptor ligand.
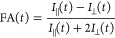
1where *I*_∥_(*t*) and *I*_⊥_(*t*) are the blank-corrected parallel and perpendicular fluorescence intensities, respectively, measured at time *t*, and FA(*t*) is the calculated fluorescence anisotropy at time *t*. FA equilibrium binding data were globally fitted using a modified version of a previously described user-defined GraphPad Prism-compatible binding model^19^ (for the detailed equation, see the Supporting Information), which takes ligand depletion into account and yields the dissociation constant *K*_d_ and simultaneously the Y_4_R concentration of the BBV stock. FA association binding data were globally fitted to obtain association rate constants *k*_on_ and *K*_d(kin)_ values also using a previously presented user-defined GraphPad Prism-compatible binding model, which takes ligand depletion into account and requires the specification of the *k*_off_ value (the mean *k*_off_ value obtained from the dissociation experiments was used).^19^ Individual association rate constants *k*_on_ and individual p*K*_d_ values, obtained from at least three independent FA association binding experiments using six different BBV concentrations and two different concentrations of **16**, were averaged to obtain mean *k*_on_ and mean p*K*_d_ values ± SEM (cf. Table 5). Kinetically derived dissociation constants *K*_d(kin)_ were calculated from individual (*n* = 3) *k*_on_ values and the mean *k*_off_ value (cf. Table 5) according to the equation *K*_d(kin)_ = *k*_off_/*k*_on_, and averaged. The mean value ± SEM of the dissociation rate constant *k*_off_ was calculated from individual *k*_off_ values obtained from three independent FA dissociation experiments each using six different BBV concentrations and two different concentrations of **16** (GraphPad Prism 5). Data from FA competition binding experiments using **16** as the fluorescent probe (*K*_d_ = 0.14 nM, mean value from four individual FA equilibrium binding experiments) were analyzed by a four-parameter logistic equation (log(inhibitor) vs response–variable slope, GraphPad Prism 5) to obtain pIC_50_ values, which were converted to p*K*_i_ values according to the Cheng–Prusoff equation^63^ (logarithmic form) using the *K*_d_ value for **16** that is mentioned above.

#### NanoBRET Y_4_R Binding Assay

BRET-based Y_4_R binding studies with **16** (saturation binding, association, dissociation, displacement by Y_4_R reference ligands) were performed following a protocol reported for a NanoBRET Y_4_R binding assays using HEK293T cells stably expressing an N-terminally Nluc-tagged human Y_4_R.^51^ One the day prior to the experiment, HEK293T-hY_4_R-Nluc(intraECL2) cells were detached from the culture flask by trypsinization, centrifuged (500*g*, 5 min), and resuspended in FBS-free L15-HEPES. The cell density was adjusted to 1.14 × 10^6^ cells/mL, and the cells were seeded into white 96-well plates (Brand, Wertheim, Germany) (70 μL, 80,000 cells per well) and incubated overnight in a water-saturated atmosphere (only atmospheric CO_2_). On the day of the experiment, dilutions of **16**, **5**, and Y_4_R ligands studied in competition binding assays were prepared as 10-fold concentrated (compared to the final concentration) solutions in L15-HEPES supplemented with 5% BSA (in the following referred to as L15-HEPES-BSA). The addition of these feed solutions to the wells resulted in a 1:10 dilution and the final compound concentrations. The final BSA concentration in the assay was 1.5% (30 μL of a solution containing 5% BSA added to 70 μL of BSA-free medium, as outlined below).

All BRET measurements were performed at a temperature of 25 °C using a TECAN Infinite Lumi plate reader (Tecan, Crailsheim, Germany) equipped with an injection module. Bioluminescence of the nanoluciferase was detected using a 460/35 nm band-pass (460/35 BP) filter. The emission of the fluorescent ligands was detected through a 610 nm long-pass (610 LP) filter. For all experiments integration times were set as follows: 100 ms for the 460/35 BP channel and 1000 ms for the 610 LP filter to increase S/N.

For saturation binding experiments, 10 μL of L15-HEPES-BSA and 10 μL of the 10-fold concentrated solution of **16** (determination of total binding) or 10 μL of a 10 μM solution of **5** and 10 μL of the 10-fold concentrated solution of **16** (determination of unspecific binding) were added to the cells. After incubation under shaking in the dark at 22 ± 1 °C for 1.5 h, 10 μL of a 10 μM solution of the Nluc substrate coelenterazine h in L15-HEPES-BSA (obtained by a 1:200 dilution of a 2 mM stock solution in methanol) were added to each well, the plate was transferred to the preheated plate reader and luminescence signals were recorded for 15 min (5 cycles).

For association experiments, 10 μL of L15-HEPES-BSA (determination of total binding) or a 10 μM solution of **5** in L15-HEPES-BSA (determination of unspecific binding) were added to the cells followed by the addition of 10 μL of the feed solution of the Nluc substrate furimazine (obtained by diluting the furimazine stock 1:1000 with L15-HEPES-BSA). The plate was immediately transferred to the plate reader, luminescence was measured for one cycle (ca. 15 s) followed by the addition of 10 μL of a 10 nM solution of **16** (final concentration: 1 nM) to each well via the injector module and continued measurement for 60 min (300 cycles).

For dissociation experiments, 10 μL of L15-HEPES-BSA (determination of total binding) or 10 μL of a 10 μM solution of **5** in L15-HEPES-BSA (determination of unspecific binding) were added to the cells followed by the addition of 10 μL of a 35 nM solution of **16** in L15-HEPES-BSA and incubation under shaking in the dark at 22 ± 1 °C for 1.5 h. 10 μL of the feed solution of the Nluc substrate furimazine (obtained by diluting the furimazine stock 1:600 with L15-HEPES-BSA) were added to each well and the plate was immediately transferred to the plate reader. Luminescence was measured for five cycles (1.5 min) followed by the addition of 10 μL of a 10 μM solution of **5** in L15-HEPES-BSA to each well with a pipette and continued measurement of luminescence for 150 min (750 cycles). In order to take the time span between the addition of the 10 μM solution of **5** (initiation of the dissociation) and the start of the measurement into account for data analysis, this delay was determined with a stopwatch.

For competition binding experiments, 10 μL of a 10-fold concentrated solution of the competing ligand (determination of total binding in the presence of a competitor), 10 μL of L15-HEPES-BSA (determination of total binding in the absence of competitor), or 10 μL of a 10 μM solution of **5** in L15-HEPES-BSA (determination of unspecific binding) were added to the cells. After a short period of preincubation (5 min), 10 μL of a 15 nM solution of **16** in L15-HEPES-BSA were added followed by incubation under gentle shaking in the dark at 22 ± 2 °C for 1.5 h. 10 μL of the 10 μM feed solution of the Nluc substrate coelenterazine h in L15-HEPES-BSA (obtained as described above) were added, the plate was immediately transferred to the plate reader and luminescence was measured for 15 min (5 cycles).

BRET ratios were calculated by dividing the signal measured in the 610LP channel through the signal obtained from the 460/35 BP channel. Corrected BRET ratios were calculated by subtracting the BRET ratio obtained from a vehicle control sample (no receptor ligand added) from the BRET ratios obtained from samples containing fluorescent ligand. Specific binding data were obtained by subtracting unspecific binding from total binding. Specific binding data from saturation binding experiments were analyzed by an equation describing a hyperbolic isotherm (binding-saturation: one site-specific binding, GraphPad Prism 5) yielding *K*_d_ values, which were converted to p*K*_d_ values. Specific binding data from association experiments were analyzed by a three-parameter equation describing a monophasic exponential rise to a maximum (*Y*_0_ constrained to zero) yielding *k*_obs(mono)_ values, and by a five-parameter equation describing a biphasic exponential rise to a maximum (*Y*_0_ constrained to zero) affording *k*_obs(bi,slow)_ and *k*_obs(bi,fast)_ values (GraphPad Prism 5). Specific binding data from dissociation experiments were analyzed by a three-parameter equation describing a monophasic exponential decline (*Y*_0_ constrained to 100%) yielding *k*_off(mono)_ values, and by a five-parameter equation describing a biphasic exponential decline (*Y*_0_ constrained to 100%, plateau constrained to zero) yielding *k*_off(bi,fast)_ and *k*_off(bi,slow)_ values (GraphPad Prism 5.0). Note: Dissociation data were initially analyzed with a variable plateau. As the plateau mean value was not significantly different from zero in the case of the biphasic analysis (*P* > 0.05, *t* test), data were reanalyzed with the plateau value constrained to zero.

The association rate constants *k*_on(mono)_, *k*_on(bi,fast)_, and *k*_on(bi,slow)_ were calculated from the observed association rate constants *k*_obs(mono)_, *k*_obs(bi,fast)_, and *k*_obs(bi,slow)_, respectively (mean values), the mean values of the dissociation rate constants *k*_off(mono)_, *k*_off(bi,fast)_, or *k*_off(bi,slow)_ (cf. Table 6), and the fluorescent ligand concentration used for the association experiments (see above) according to the equation *k*_on_ = (*k*_obs_ – *k*_off_)/[fluorescent ligand]. The kinetically derived dissociation constants *K*_d(kin)_ were calculated from *k*_on(mono)_, *k*_on(bi,fast)_, or *k*_on(bi,slow)_ and the mean values of the dissociation rate constants *k*_off(mono)_, *k*_off(bi,fast)_, or *k*_off(bi,slow)_ (cf. Table 6) according to the equation *K*_d(kin)_ = *k*_off_/*k*_on_.

Total binding data from competition binding experiments were plotted as corrected BRET ratio over log(competitor concentration, *M*) and analyzed by a four-parameter logistic equation (log(inhibitor) vs response–variable slope, GraphPad Prism 5) to obtain the TOP value (upper curve plateau), which was used for data normalization (100% = TOP value, 0% = average of the corrected BRET ratio obtained from unspecific binding). The normalized data (%) were plotted over log(competitor concentration, *M*) and analyzed by a four-parameter logistic equation to obtain pIC_50_ values, which were converted to p*K*_i_ values according to the Cheng–Prusoff equation^63^ (logarithmic form) using the following *K*_d_ value for **16**: *K*_d_ = 0.94 nM (mean value from three individual saturation binding experiments).

#### Confocal Microscopy

Confocal microscopy was performed with a Zeiss LSM 710 confocal laser scanning microscope (Zeiss). The objective was 63× magnification with oil (1.4 NA). One day prior to the experiment CHO-hY_4_R-G_qi5_-mtAEQ cells were seeded in Nunc LabTekTM II cover glasses with 8 chambers (Thermo Fisher Scientific). On the day of the experiment, the confluency of the cells was 60–80%. The culture medium was removed, cells were washed with L15 medium (200 μL) and covered with L15 medium (100 μL) containing H33342 (2 μg/mL). L15 medium (100 μL) containing H33342 (2 μg/mL) and either compound **16** or **17** (final concentration: 20 nM) were added for total binding. For the determination of unspecific binding, L15 medium (100 μL) containing H33342 (2 μg/mL) and hPP (final concentration: 1 μM), and L15 medium (100 μL) containing H33342 (2 μg/mL) and either **16** or **17** (final concentration: 20 nM) were added. Images were acquired after an incubation period of 30 min (37 °C). The settings for compound **16** were: laser power/pinhole: 405 nm: 1.8%/1.97 airy units, 561 nm: 2%/1.32 airy units. The settings for compound **17** were: laser power/pinhole: 405 nm: 2.0%/1.90 airy units, 633 nm: 28%/1.35 airy units. Filter settings for fluorescence detection: 410–549 nm (H33342) (studies with **16**), 410–585 nm (H33342) (studies with **17**), 562–649 nm (**16**), and 638–759 nm (**17**).

#### Wide-Field Epifluorescence and TIRF Microscopy

For fluorescent ligand binding imaging experiments, human SK-OV-3 ovarian adenocarcinoma cells (ATCC HTB-77) were seeded at a density of 20,000 cells per well into eight-well CG imaging chambers (Zell Kontakt GmbH, Nörten-Hardenberg, Germany). After incubation for 24 h in McCoy’s 5A Medium (supplemented with 10% FBS, 2 mM l-glutamine, 100 U/mL penicillin, and 100 μg/mL streptomycin) at 37 °C in a humidified atmosphere (95% air, 5% CO_2_), recombinant MultiBacMam baculoviruses of Y_4_R^VSVG^ were added (cell confluency: approximately 70%) at an MOI of 3 and incubated for additional 24 h (medium was supplemented with 1 mM sodium butyrate). Native cells without the addition of Y_4_R baculovirus were used as a negative control. Prior to imaging, nuclei were stained with 2 mM (final concentration) Hoechst 34580 (Chemodex Ltd., St. Gallen, Switzerland) for 5 min. The culture medium was replaced by LiveLight MEMO imaging medium (Cell Guidance Systems, Cambridge, U.K.) (200 μL, supplemented with supplement A according to the manufacturer’s protocol). After incubation at 37 °C in a humidified atmosphere containing 5% CO_2_ for 1 h, compound **16** (final concentration 0.1 or 1 nM) was added. In the case of unspecific binding, hPP was additionally added at a final concentration of 10 μM. Cells were imaged after 30 min of incubation (37 °C, atmosphere with 5% CO_2_). Imaging was performed using a microscope setup as described previously.^66^ Briefly, wide-field epifluorescence and TIRF imaging were performed using an inverted microscope based on a Till iMIC body (Till Photonics/FEI, Munich, Germany), equipped with a UPLSAPO 20× oil (NA 0.85) and TIRF APON 60× oil (NA 1.49) objectives (Olympus Co., Tokyo, Japan). The samples were alternately excited using a 405 nm (120 mW) PhoxX laser diode (Omicron-Laserage, Rodgau, Germany) for Hoechst 34580 or at 561 nm (106 mW) for compound **16**. Lasers were combined in a SOLE-6 light engine (Omicron-Laserage) and their emission was coupled into a Yanus scan head, which, along with a Polytrope galvanometric mirror (Till Photonics/FEI), was used to position the laser beam for wide-field epifluorescence or TIRF (approximately 100 nm depth) illumination. Excitation and emission light were spectrally separated using an imaging filter cube containing a flat 2 mm beamsplitter zt 405/488/531/640rpc (Chroma Technology Co.) and a TIRF emission filter ZET 405/488/561/640 (Chroma Technology Co.). In addition, a bright-field channel was used to define cell boundaries. The electron-multiplying charge-coupled device Ultra 897 camera (Andor Technology, Belfast, U.K.) was mounted to the microscope via a TuCam adapter with 2× magnification (Andor Technology). The camera was cooled to −100 °C using an Oasis 160 liquid recirculating chiller (Solid State Cooling Systems, Wappingers Falls, NY). All measurements were performed in the eight-well CG imaging chambers, and at least 10 selected areas per well were recorded as 16-bit multicolor OME-TIFF Z-stacks (100 frames, with 200 nm piezo-focusing increment in the case of the 60× objective or 500 nm in case of 20× objective), with an exposure time of 100 ms and an EM gain of 300 in the case of wide-field epifluorescence imaging or one-color time stacks (1000 frames at 30 Hz) in the case of single-particle tracking TIRF imaging. For different conditions, the laser powers were varied to enable optimal signal levels (the absence of a detectable crosstalk signal in the channels was ensured). Wide-field epifluorescence Z-stacks were deconvolved using the EpiDEMIC plugin.^67^ Single-particle time lapse tracking was performed in the Icy image analysis environment using the Spot Tracking^68^ plugin and classified using Track Manager. Tracks longer than 150 frames were filtered out for video presentation.

#### Statistical Significance

For the applied statistical tests (*F*-test, *t* test), the significance level was set to *P* = 0.05.

#### Calculation of Propagated Errors

Propagated errors (applying to specifically bound fluorescent ligand (saturation binding), association rate constants *k*_on_, and kinetically derived dissociation constants *K*_d(kin)_ obtained from flow cytometric and NanoBRET assays) were calculated as described elsewhere.^31^

## Supporting Information Available

Preparation of the azido-functionalized Py-5 derivative **15**; chromatograms of the investigation of the chemical stability of **16**–**18** in PBS pH 7.4 (Figure S1); radioligand displacement curves from competition binding experiments with [^3^H]UR-KK200 (Figure S2); concentration–response curves of hPP, **11** and **16**–**19** obtained from a hY_4_R Ca^2+^ aequorin assay (Figure S3); concentration–response curves of hPP, **5**, **11**, and **16**–**19** obtained from a hY_4_R mini-G_si_ protein recruitment assay (Figure S4); concentration–response curves of hPP, **16** and **17** obtained from a hY_4_R CAMYEN cAMP assay (Figure S5); study of dummy fluorescent ligands in functional assays (Figure S6); excitation and emission spectra (Figure S7); viability of CHO-hY_4_R-G_qi5_-mtAEQ cells (Figure S8); analyses of flow cytometric saturation binding data of **16** based on nonviable and viable cell populations (Figure S9); syntax of the equation used to fit FA equilibrium binding data (GraphPad Prism 5); RP-HPLC chromatograms of compounds **11** and **16**–**19**; and ^1^H NMR spectra of compound **11** (PDF)
TIRF video sequence (recorded and shown at a 30 Hz resolution, shown at double speed) showing the interactions of **16** with the basal plasma membrane of adherent SK-OV-3-Y_4_R cells including single-particle tracking (magnified region) (AVI)

## Supplementary Material

pt4c00013_si_001.pdf

pt4c00013_si_002.avi

## Abbreviations Used

Arg(carb): *N*^ω^-carbamoylated arginine containing a tetramethylene spacer or a hexyne structure with a terminal alkyne group in the carbamoyl substituent (cf. Figure 3)
BSA: bovine serum albumin
CAMYEN: cAMP sensor using YFP-Epac-nanoluciferase
CHO: Chinese hamster ovary
Cy3B-SE: Cy3B succinimidyl ester
DIPEA: *N*,*N*-diisopropylethylamine
*E*_max_: efficacy (maximum effect) of a receptor agonist determined in a functional assay
Epac: exchange protein activated by cAMP
EtOH: ethanol
FA: fluorescence anisotropy
FBS: fetal bovine serum
FL: fluorescent ligand
Fmoc amino acid: *N*^α^-Fmoc-protected and side-chain-protected (if required) amino acid
HBTU: 3-[bis(dimethylamino)methyliumyl]-3*H*-benzotriazol-1-oxide hexafluorophosphate
HEC: human endometrial cancer
HEPES: 4-(2-hydroxyethyl)-1-piperazineethanesulfonic acid
HOBt: hydroxybenzotriazole
hPP: human pancreatic polypeptide
hY_4_R: human Y_4_ receptor
*k*: retention (or capacity) factor (HPLC)
MeOH: methanol
Nluc: nanoluciferase
Pbf: 2,2,4,6,7-pentamethyl-dihydrobenzofuran-5-sulfonyl
PBS: phosphate-buffered saline
pEC_50_: negative decadic logarithm of the half-maximal effective concentration (functional assays)
p*K*_i_: negative decadic logarithm of the dissociation constant *K*_i_ (in M) obtained from a competition binding experiment
pNPY: porcine neuropeptide Y
PS: polystyrene
PyBOP: benzotriazol-1-yl-oxytripyrrolidinophosphonium-hexafluorophosphate
PYY: peptide YY
RP: reversed phase
SEM: standard error of the mean
SPPS: solid-phase peptide synthesis
TFA: trifluoroacetic acid
TIRF: total internal reflection fluorescence
*t*_R_: retention time
YFP: yellow fluorescent protein
YR: NPY receptor
