# Supplementary material for: Illuminating Neuropeptide Y Y4 Receptor Binding: Fluorescent Cyclic Peptides with Subnanomolar Binding Affinity as Novel Molecular Tools
Source: ACS Pharmacol Transl Sci. 2024 Mar 20;7(4):1142–68. doi: 10.1021/acsptsci.4c00013 (PMC11019746; doi:10.1021/acsptsci.4c00013)
Supplement: Supplementary file 1 — pt4c00013_si_001.pdf [file pt4c00013_si_001.pdf]

Supporting information

Illuminating neuropeptide Y Y_4 receptor binding: fluorescent cyclic peptides with subnanomolar binding affinity as novel molecular tools

Jakob Gleixner, Sergei Kopanchuk, Lukas Grätz, Maris-Johanna Tahk, Tõnis Laasfeld, Santa Veikšina, Carina Höring, Albert O. Gattor, Laura J. Humphrys, Christoph Müller, Nataliya Archipowa, Johannes Köckenberger, Markus R. Heinrich, Roger Jan Kutta, Ago Rinken and Max Keller**

*E-mail addresses: max.keller@chemie.uni-regensburg.de
ago.rinken@ut.ee

Content

1. Preparation of the azido-functionalized Py-5 derivative **15** S2

2. Figures S1-S9 S4

3. Syntax of the equation used to fit FA equilibrium binding data (GraphPad Prism 5) S13

4. RP-HPLC chromatograms of compounds **11** and **16-19** S16

5. ^{1}H-NMR spectra of compound **11** S18

6. References S19

1. Preparation of the azido-functionalized Py-5 derivative 15

Treatment of t*ert*-butyl (3-bromopropyl)carbamate (**20**) with sodium azide followed by Boc deprotection of the amino group yielded amine **21** (Scheme S1). The latter was used to convert the pyrylium dye **22** (Py-5) to the azide-functionalized cyanine dye **15**.

Scheme S1. Synthesis of the Py-5 derivative **15**.

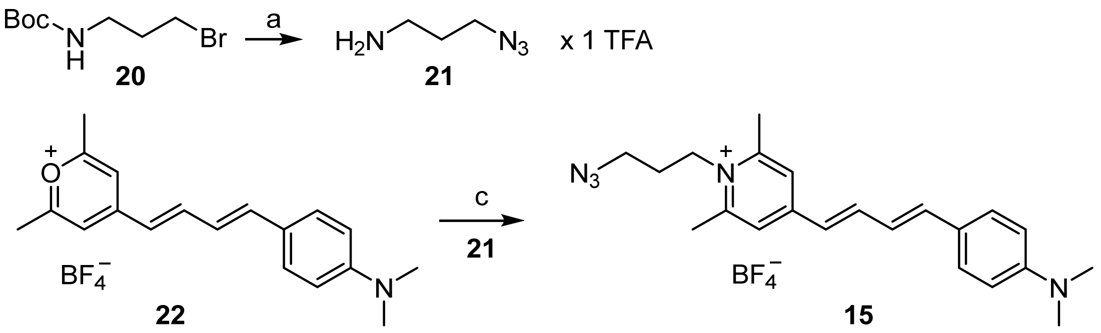

Reagents and conditions: (a) (1) sodium azide, DMF, 60 °C, 16 h, (2) TFA/CH_2Cl_2 1:5 v/v, rt, 16 h, 50% (c) **21,** triethylamine, ethanol, rt, 1 h, 38%

Synthesis protocols

3-Azidopropane-1-amine hydrotrifluoroacetate (21)[1]

Tert-butyl (3-bromopropyl)carbamate (**20**) (250 mg, 1.1 mmol) was dissolved in DMF (30 mL) followed by the addition of sodium azide (75 mg, 1.2 mmol). The mixture was stirred overnight under reflux. After cooling to rt, water (30 mL) was added, and the mixture was transferred into a separation funnel followed by extraction with EtOAc (3 x 50 mL). The organic phases were combined, washed with brine (1 x 50 mL) and dried over Na_2SO_4. The volatiles were removed in vacuo, the residue was dissolved in TFA/CH_2Cl_2 1:5 v/v (5 mL), and the mixture was stirred at rt for 16 h. Removal of the volatiles in vacuo yielded **21** as a brownish oil (118 mg, 50%), which was used without further purification. ^{1}H-NMR (400 MHz, CD_3OD): δ (ppm) 1.84-1.96 (m, 2H), 2.84-2.88 (m, 2H), 2.97-3.07 (m, 3H), 3.49 (t, 2H, *J* 6.4 Hz). ^{13}C-NMR-DEPT135 (100 MHz, CD_3OD): δ (ppm) 26.49, 37.03, 48.10. HRMS (ESI): *m/z* $[M+H]^+$ calcd. for $[C_3H_9N_4]^+$ 101.0822, found: 101.0821. $C_3H_8N_4 \cdot C_2HF_3O_2$ (101.13 + 114.02)

1-(3-Azidopropyl)-4-((1*E*,3*E*)-4-(4-(dimethylamino)phenyl)buta-1,3-dien-1-yl)-2,6-dimethylpyridin-1-ium tetrafluoroborate (15)

Compounds **21** (23.3 mg, 109 µmol) and **22** (45.8 mg, 125 µmol) (note: **22** was used as tetrafluoroborate salt) were dissolved in ethanol (5 mL). Triethylamine (45 µL) was added, and the mixture was stirred at rt in the dark for 1 h. The volatiles were removed in vacuo and purification by column chromatography (CH_2Cl_2/methanol 9:1 v/v, isocratic) yielded **15** as a red solid (18.8 mg, 38%). ^{1}H-NMR (400 MHz, DMSO-d_6): δ (ppm) 1.22-1.27 (m, 2H), 1.97-2.09 (m, 2H), 2.77 (s, 6H), 2.98 (s, 6H), 4.33-4.46 (m, 2H), 6.56-6.63 (m, 1H), 6.71-6.75 (m, 2H), 6.92-7.07 (m, 2H), 7.43-7.49 (m, 2H), 7.61-7.75 (m, 1H), 7.86 (s, 2H). HRMS (ESI): *m/z* $[M]^+$ calcd. for $[C_{22}H_{28}N_5]^+$ 362.2340, found: 362.2328. $C_{22}H_{28}N_5^+ \cdot BF_4^-$ (362.50 + 86.81)

2. Figures S1-S9

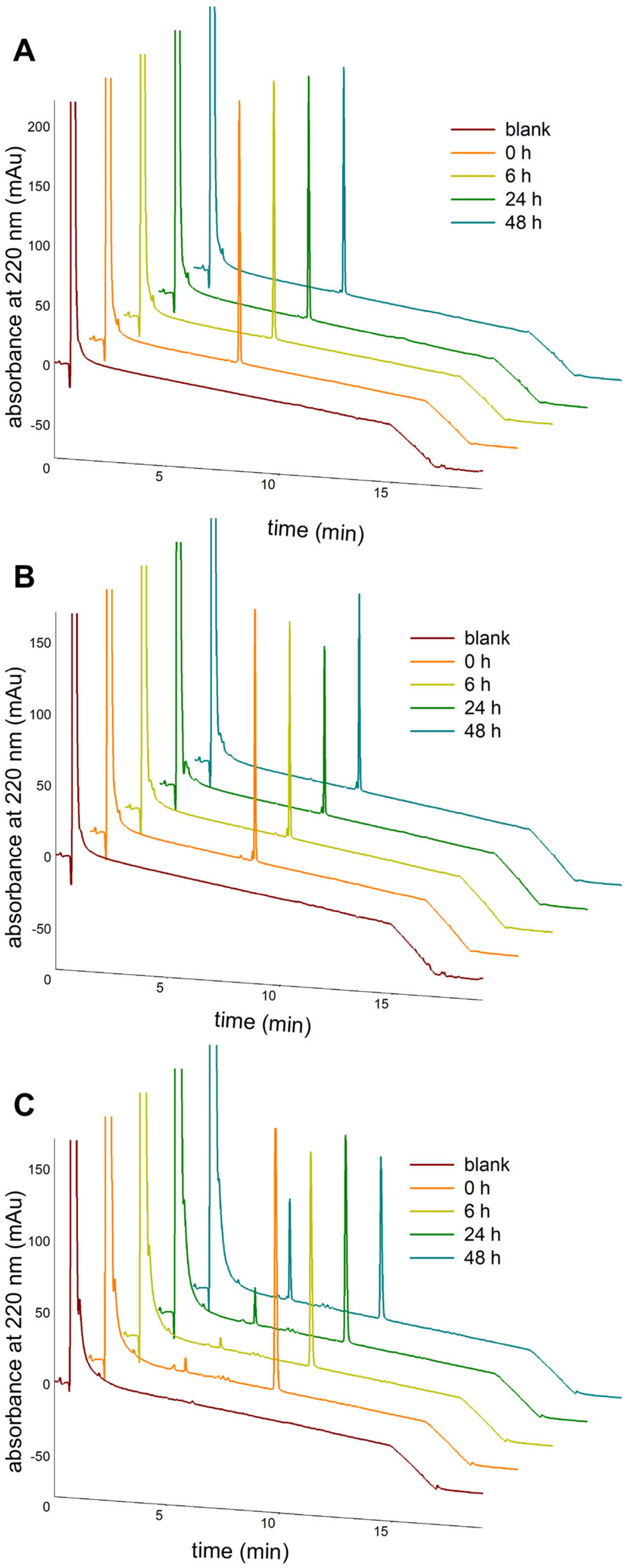

Figure S1. Investigation of the chemical stability of **16** (**A**), **17** (**B**) and **18** (**C**) in aqueous solution at pH = 7.4. Chromatograms of the RP-HPLC analysis of **16**, **17**, and **18** after incubation in PBS pH = 7.4 for up to 48 h. Whereas **16** and **17** showed no decomposition, **18** proved to be unstable.

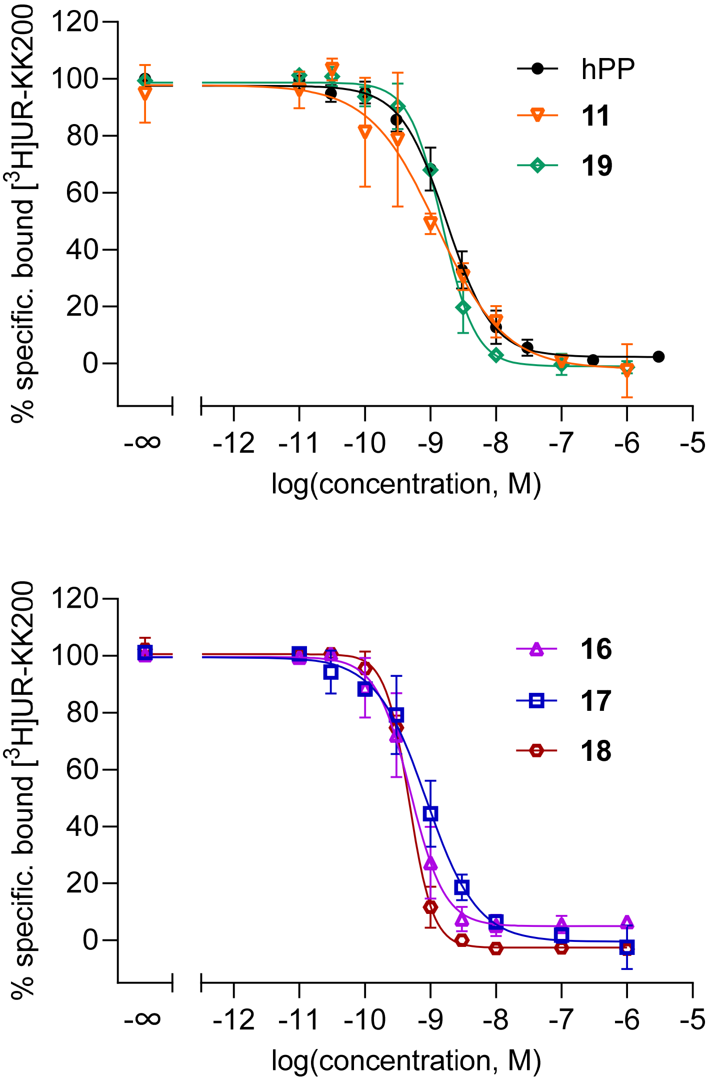

Figure S2. Radioligand displacement curves from competition binding experiments with [^{3}H]UR-KK200 (K_d = 0.67 nM, c = 1 nM) and hPP, **11**, and **16-19** at intact CHO-hY$_4$R-G$_{qi5}$-mtAEQ cells. Data represent mean values ± SEM from 3-5 independent experiments (performed in triplicate). Data of hPP were taken from Wirth et al.[2] For pK_i values, see Table 1 (main article)

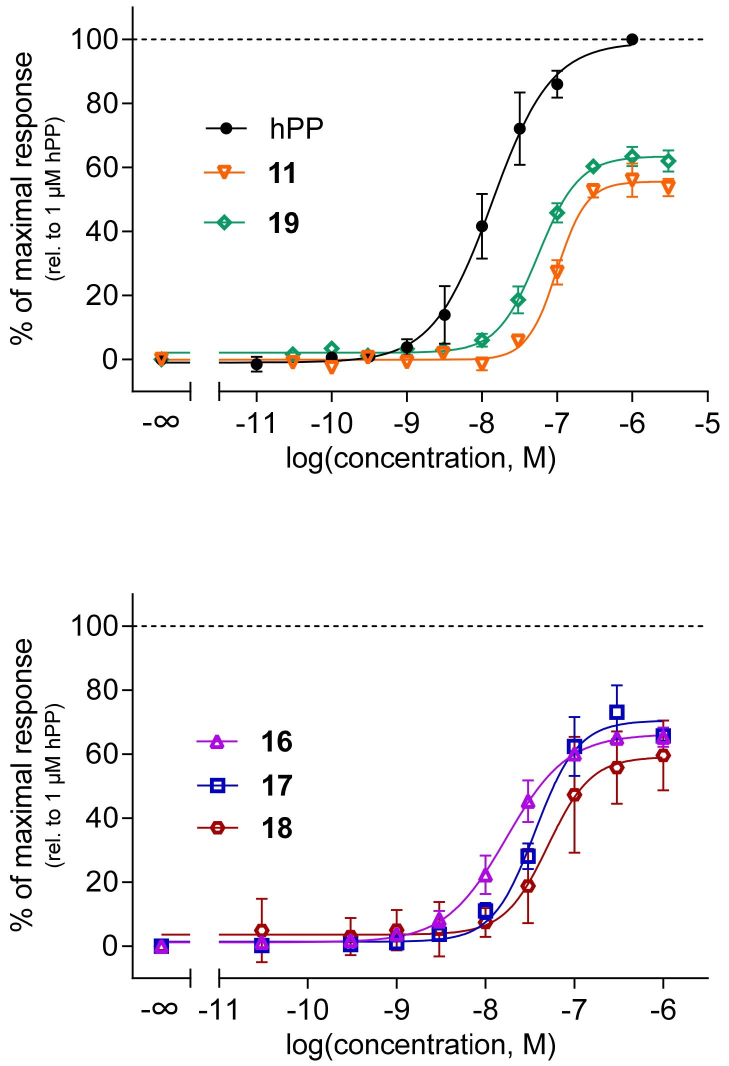

Figure S3. Concentration-response curves of hPP, **11**, and **16-19** obtained from a Ca^{2+} aequorin assay performed with CHO-hY_4R-G_{qi5}-mtAEQ cells. Presented are mean values ± SEM from three or four independent experiments (performed in triplicate). Data of hPP were taken from Gleixner et al.[3] For pEC_{50} values, see Table 2 (main article).

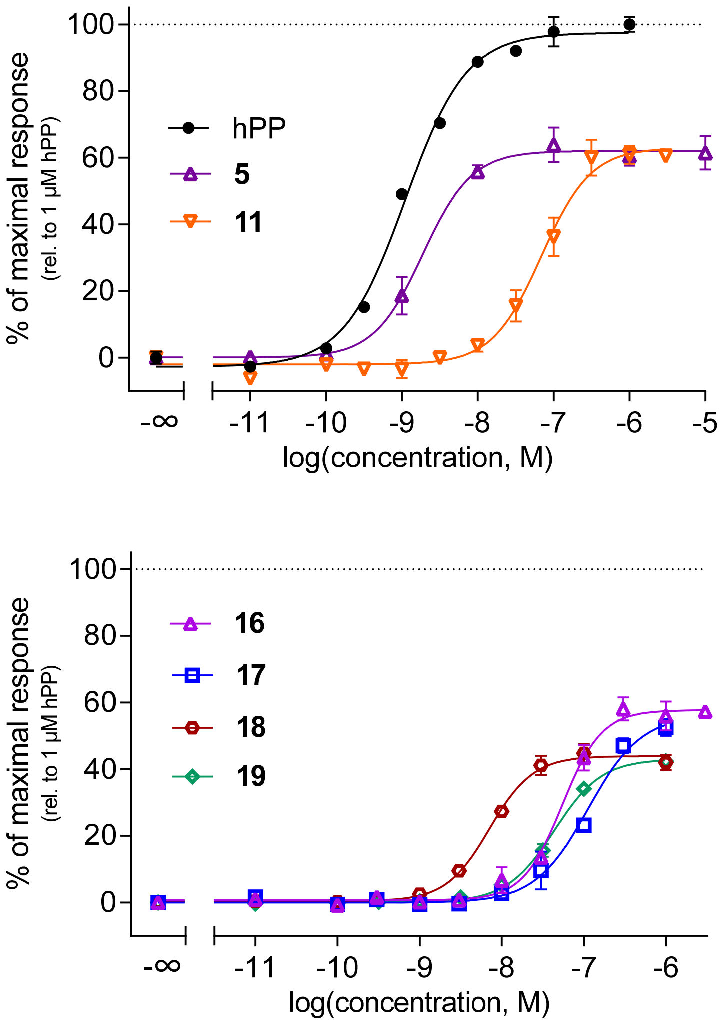

Figure S4. Concentration-response curves of hPP, **5, 11**, and **16-19** obtained from a hY$_4$R miniG$_{si}$ protein recruitment assay performed with HEK293T-NlucN-mG$_{si}$/Y$_4$R-NlucC cells. Presented are mean values ± SEM from three independent experiments (performed in triplicate). Data of hPP were taken from Gleixner et al.[3] For pEC$_{50}$ values, see Table 2 (main article).

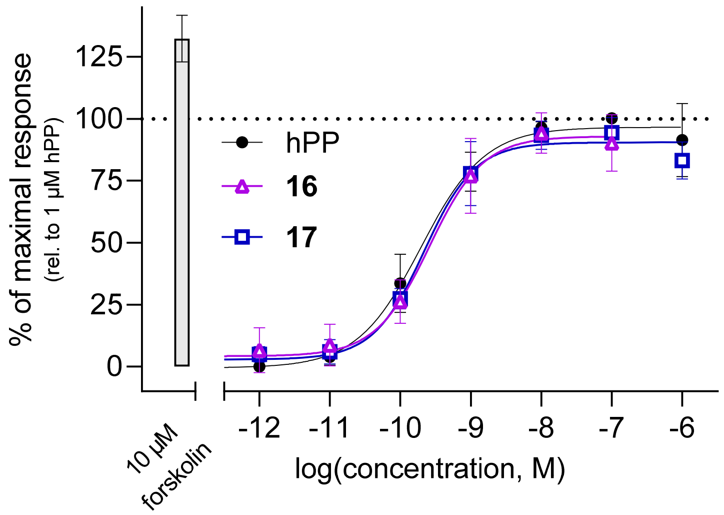

Figure S5. Concentration-response curves of hPP, **16** and **17**, obtained from a hY_4R CAMYEN cAMP assay performed with HEK293T-CAMYEN-hY_4R cells. Presented are mean values ± SEM from three independent experiments (performed in triplicate). Data of hPP were taken from Gleixner et al.[3] For For pEC_{50} values, see Table 2 (main article).

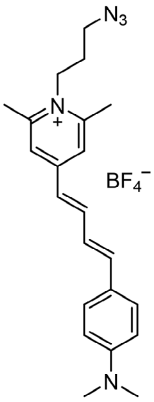

15

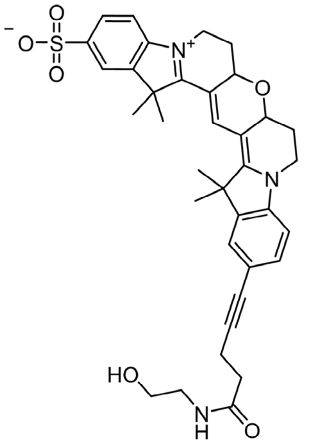

23

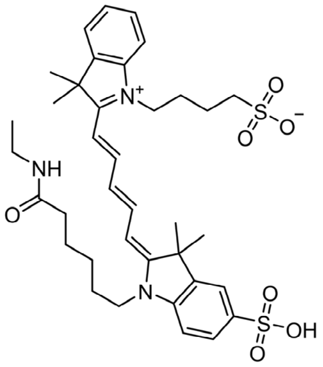

24

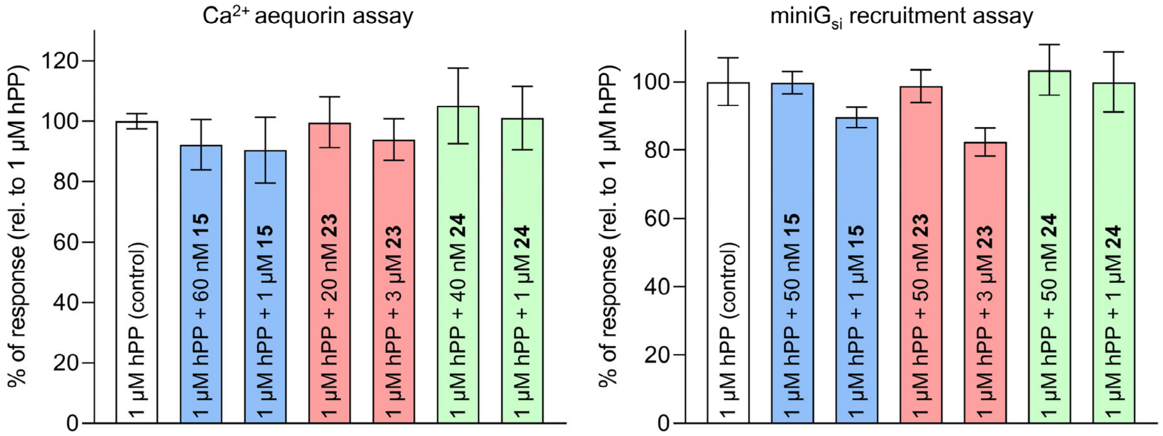

Figure S6. Potential impact of the fluorophores in the fluorescent ligands **16, 17** and **19** on the readout of the Ca^{2+} aequorin and the miniG$_{si}$ recruitment assay studied with the fluorescent dummy ligands **15, 23** and **24** used at a concentration approximately corresponding to the EC_{50} of **16**, **17** and **19**, and at a second concentration corresponding to the highest concentration of **16, 17** and **19** in the functional assays. Data represent mean values ± SEM (shown as error bar), obtained from three independent experiments each performed in sextuplicate. The effect elicited by 1 µM hPP was not or only marginally affected by the presence of the fluorescence dummy ligands.

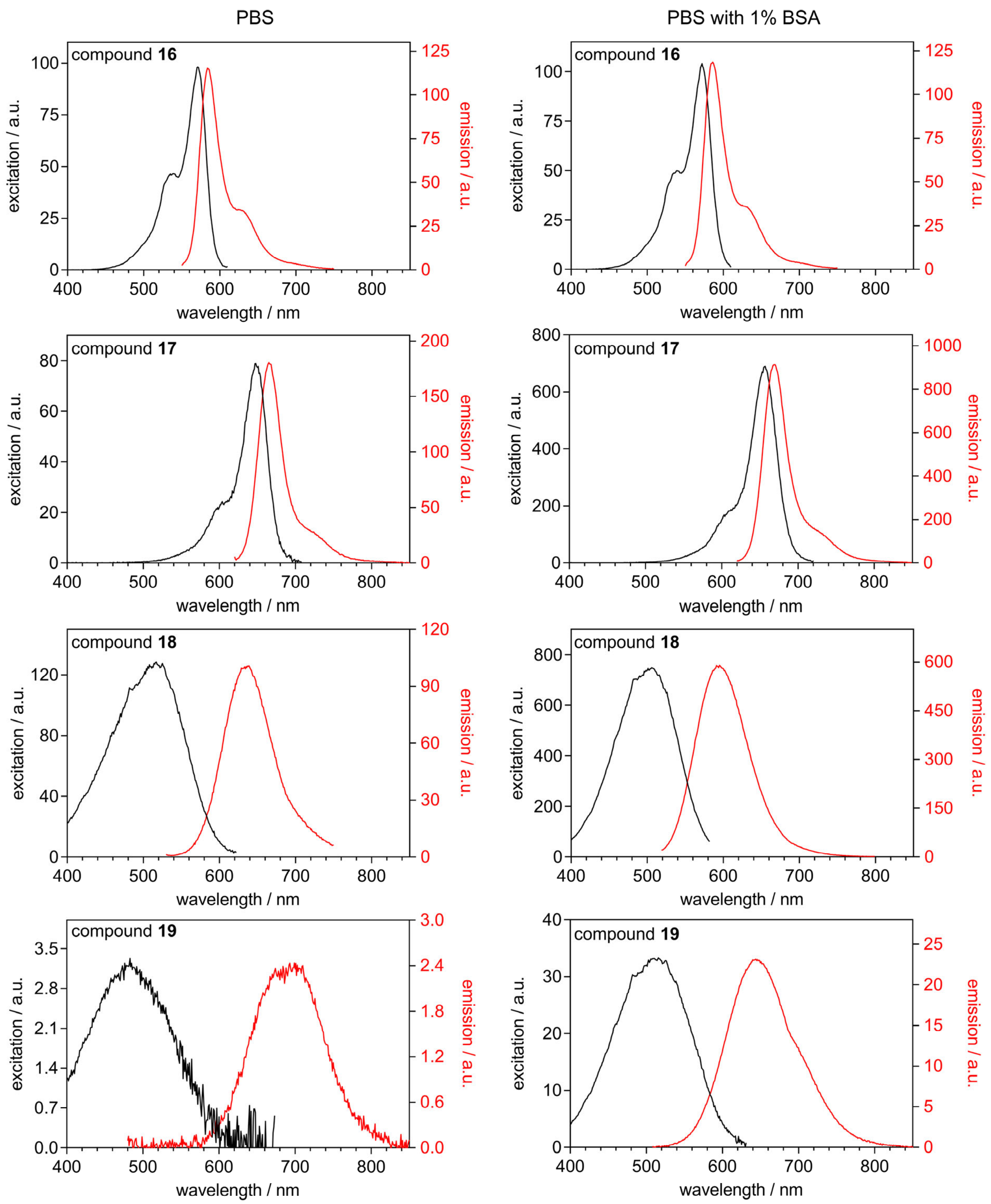

Figure S7. Excitation (black line) and emission (red line) spectra of fluorescent ligands **16-19** recorded in PBS (pH 7.4) and PBS supplemented with 1% BSA at 22 °C. The fluorescent ligand concentrations were 1 µM (**16-17** and **18** in PBS with 1% BSA), 5 µM (**18** in PBS) and 6 µM (**19**).

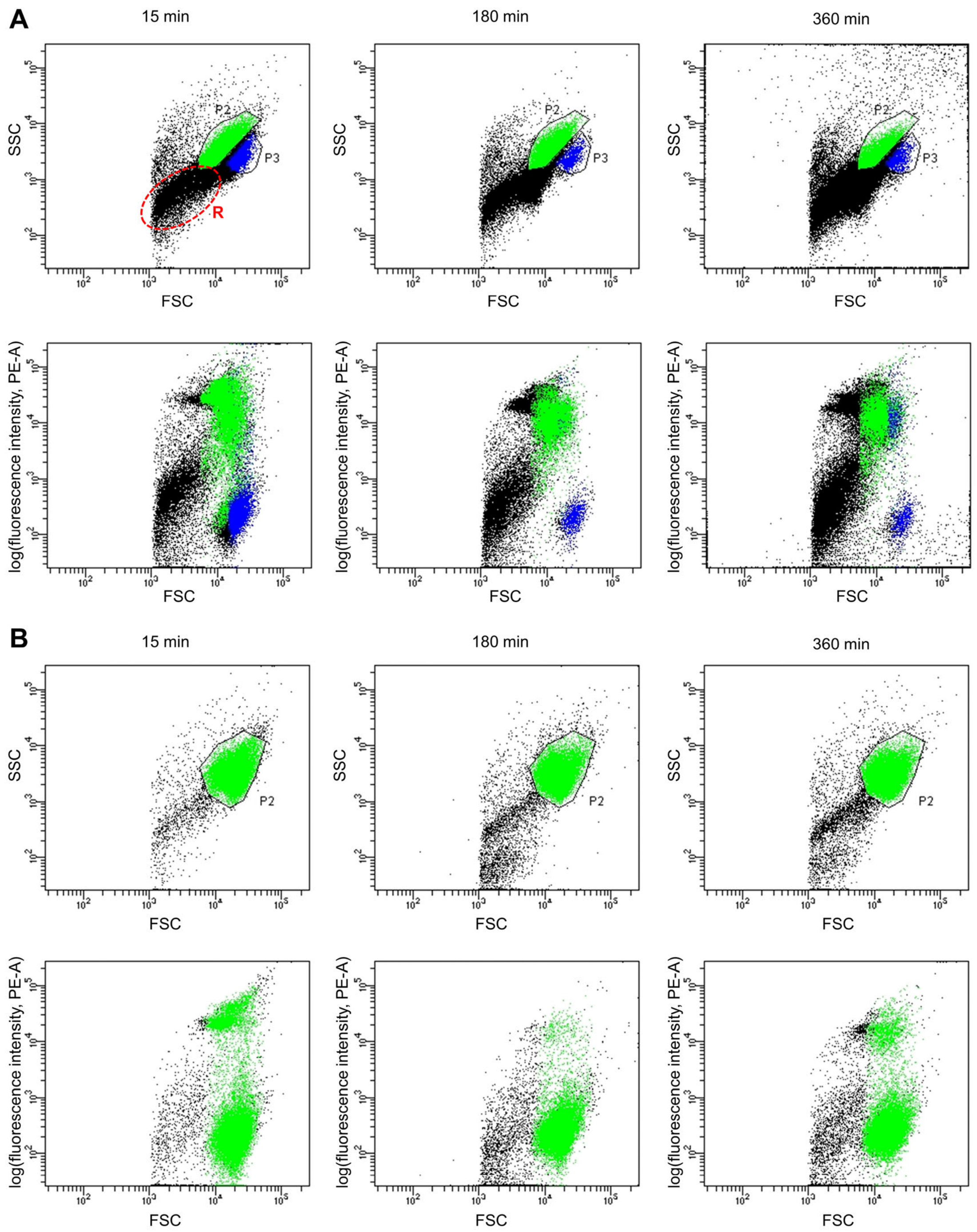

Figure S8. Viability of CHO-hY$_4$R-G$_{qi5}$-mtAEQ cells studied by flow cytometry using propidium iodide (2 µg/mL) as staining agent (added 1 min prior to measurement). (A) Analysis of a cell suspension in sodium-free *buffer I* (gently shaken at 22 °C) after different times. Dot plots in the upper panel show sideward scatter (SSC) plotted against forward scatter (FSC) and dot plots in the lower panel show propidium fluorescence (detected in the PE-A channel) plotted over FSC. The cell population P2 represents non-viable cells (high propidium uptake) and the population P3 represents intact cells (low propidium uptake). The same experiment, performed in the presence of the Y$_4$R partial agonist **6** (1.5 nM), gave the same result (data not shown). Counting events marked by the red oval (R) represent necrotic cells and apoptotic bodies showing low fluorescent ligand binding (data not shown). (B) Analysis of a cell suspension in *DPBS* (gently shaken at 22 °C) after different times. Dot plots in the upper panel show SSC plotted against FSC and dot plots in the lower panel show propidium fluorescence plotted over FSC. The cell population P2 represents intact cells (low propidium uptake). The number of non-viable cells (high propidium uptake), not appearing as a separate population, was low.

A

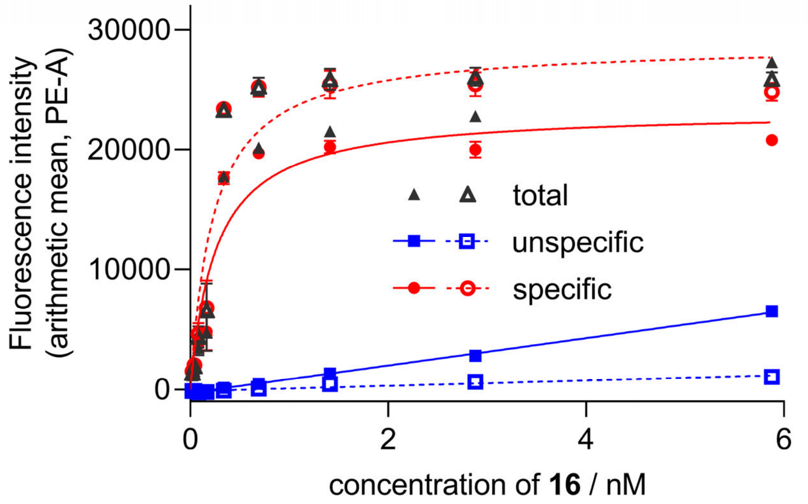

B

cell population	individual K_d values [nM]	mean K_d [nM]
non-viable (corresp. to population P2 in Figure S8A)	0.20, 0.43, 0.26	0.30
viable (corresp. to population P3 in Figure S8A)	0.21, 0.39, 0.23	0.28

Figure S9. Flow cytometric saturation binding data obtained from experiments with fluorescent ligand **16** at CHO-hY$_4$R-G$_{qi5}$-mtAEQ cells in sodium-free *buffer I*: comparison of the data analyses based on non-viable and viable cell populations. (A) Saturation isotherms (circles) of **16** from a representative saturation binding experiment. Filled symbols represent the data analysis based on the non-viable cell population (corresponds to population P2 in Figure S8A) (same data as shown in Figure 4A, main article). Open symbols represent the data analysis based on the viable cell population (corresponds to population P3 in Figure S8B). (B) Individual and mean K_d values of **16** resulting from the different data analyses (non-viable *vs*. viable cell population).

3. Syntax of the equation used to fit FA equilibrium binding data (GraphPad Prism 5)

```
<A>Rt=StockR*X/WellVolume

<B>Rt=0

<C>Rt=StockR*X/WellVolume

<D>Rt=0

NS=StockNS*X/WellVolume

A=Kd+Kdns+Rt+NS-Lav

B=(NS-Lav)*Kd+(Rt-Lav)*Kdns+Kd*Kdns

C=-Kd*Kdns*Lav

Theta=arccos[(-2*A^3+9*A*B-27*C)/(2*sqrt((A^2-3*B)^3))]

fractionRL_of_Rt=Rt*((2*sqrt(A^2-3*B)*cos(Theta/3)-A)/(3*Kd+2*sqrt(A^2-
3*B)*cos(Theta/3)-A))

fractionNBVL_of_NS=NS*((2*sqrt(A^2-3*B)*cos(Theta/3)-A)/(3*Kdns+2*sqrt(A^2-
3*B)*cos(Theta/3)-A))

fractionRL = fractionRL_of_Rt/Lav

fractionNS = fractionNBVL_of_NS/Lav

Stotal=fractionRL*Abound+fractionNS*Ans+Afree*(1-fractionRL-fractionNS)

Y=Stotal
```

Where:

- **X** is the volume of the baculovirus preparation in well plotted on the x axis and **Lt** constrained to the values in Column titles.
- **Rt** corresponds to the total receptor concentration in the well $[R]_T$
- **StockR** is the receptor stock concentration $[R]_{stock}$
- **VolumeBV** is the volume of the baculovirus preparation in well V_{BV}

- **WellVolume** is the total reaction volume in well V_{well}
- **NS** corresponds to the total nonspecific ligand binding site concentration in the well
- **StockNS** is the nonspecific ligand binding site concentration in the stock $[NS]_{stock}$
- **Kdns** is nonspecific binding apparent affinity K_d^{ns}
- **Lt** is the total concentration of the fluorescent ligand $[L]_T$
- **Lns** is the concentration of the fluorescent ligand that was depleted by nonspecific binding sites $[L]^{ns}$
- **Lav** is the concentration of the fluorescent ligand that is available for a specific reaction with receptors $[L]_{Av}$
- **A, B, C, Theta** are auxiliary parameters
- **RL** is the receptor-ligand complex formed $[RL]$ and reflects the amount of the fluorescent ligand that is specifically bound to the receptor
- **fractionRL** is the fraction of the ligand bound to the receptor
- **fractionNS** is the fraction of nonspecifically bound ligand
- **Stotal** is the measured anisotropy signal in absence of non-labeled ligand
- **Sns** is the measured anisotropy signal in presence of non-labeled ligand
- **Abound** is the anisotropy value of ligand specifically bound to the receptor
- **Ans** is the anisotropy value for the nonspecifically bound ligand
- **Afree** is the anisotropy value for the free ligand

The following constrains were applied:

"StockR > 0 and shared"

"WellVolume = 100"

"StockNS = 10000"

"Kd > 0 and shared"

"Kdns > 0 and shared"

"0 < Abound < 0.4 and shared"

"0 < Ans < 0.4 and shared"

"0 < Afree < 0.4 and shared"

"Lav is obtained from column title "

4. RP-HPLC chromatograms of compounds 11 and 16-19

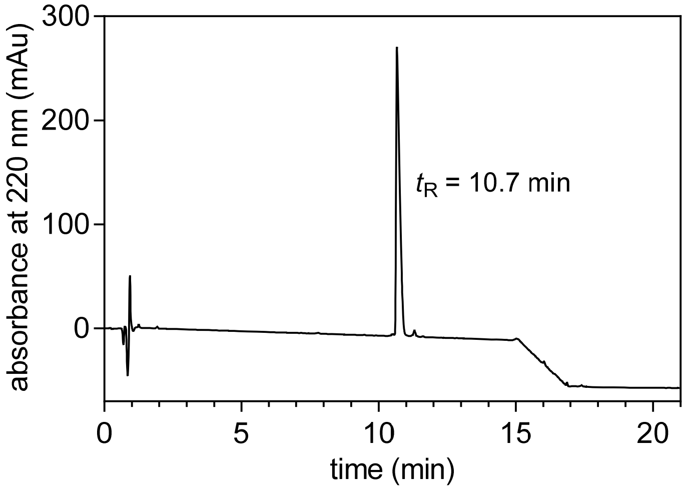

RP-HPLC analysis of compound **11**

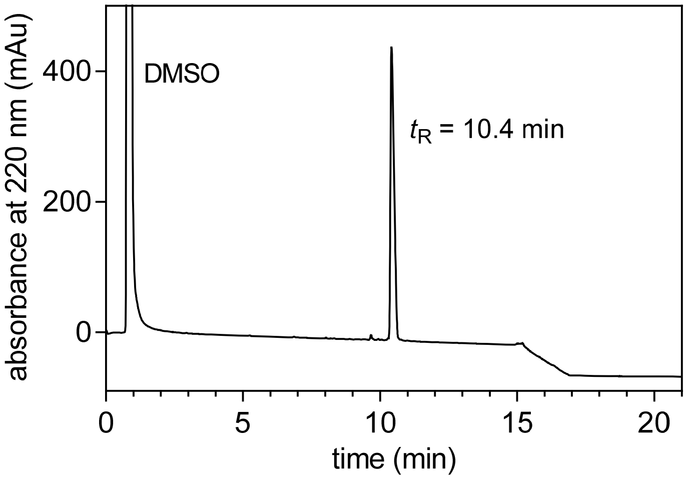

RP-HPLC analysis of compound **16**

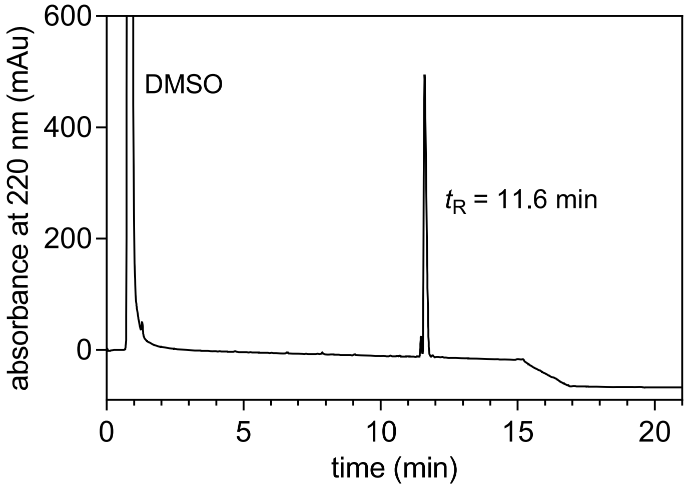

RP-HPLC analysis of compound **17**

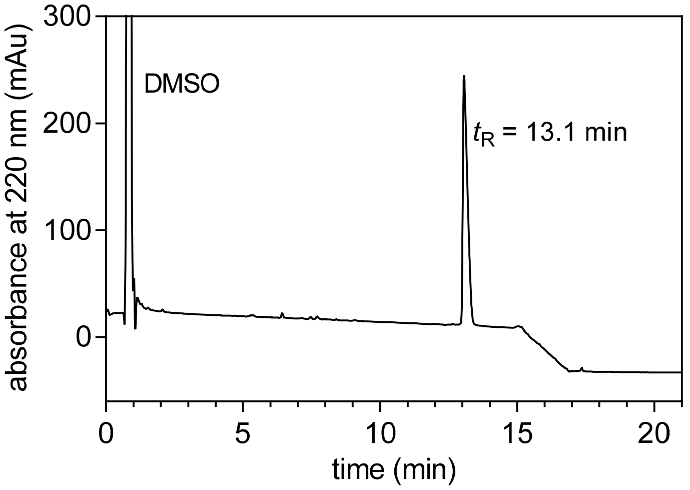

RP-HPLC analysis of compound **18**

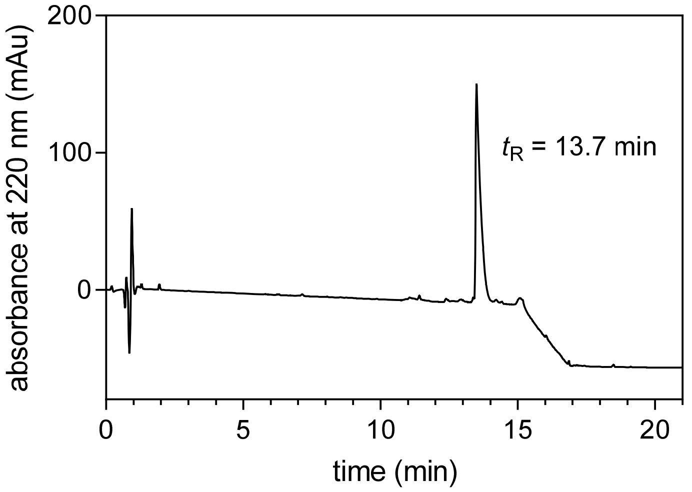

RP-HPLC analysis of compound **19**

5. ^{1}H-NMR spectra of compound 11

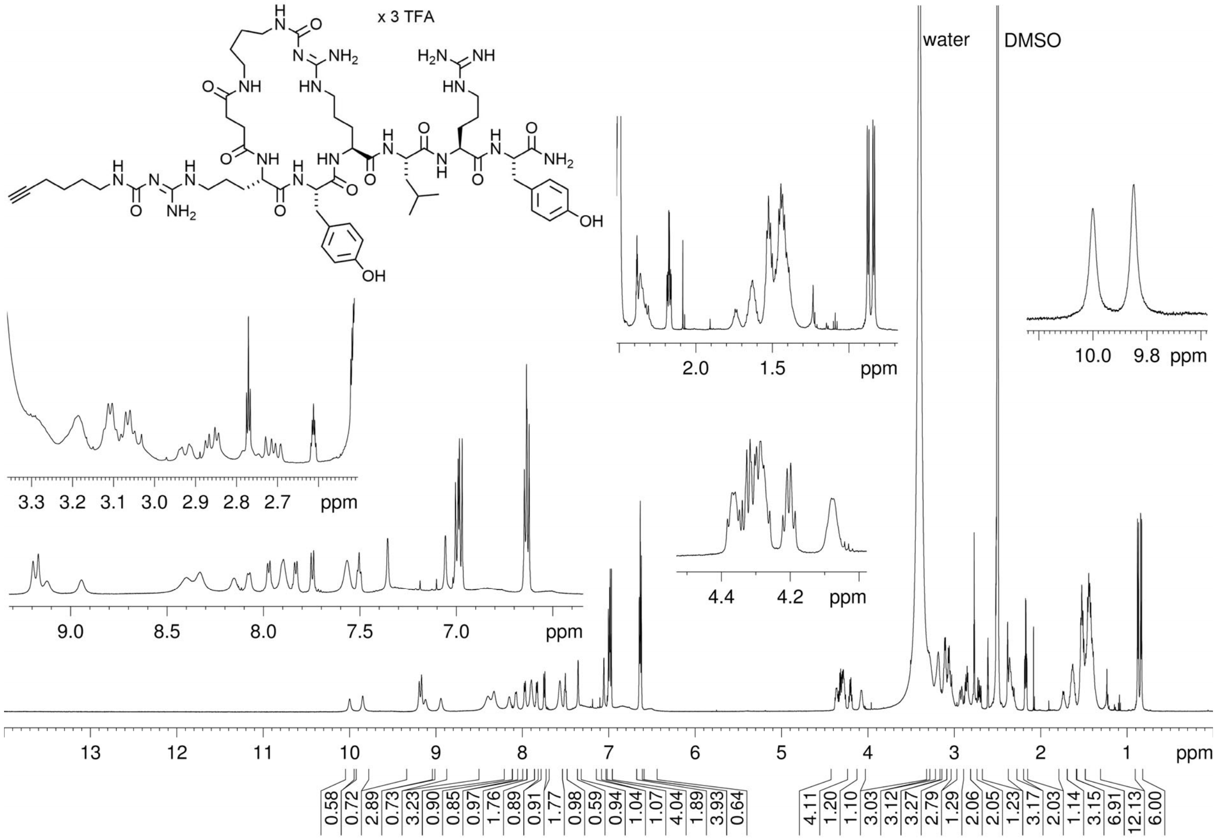

^{1}H-NMR spectrum (600 MHz, DMSO- d_6) of compound **11**

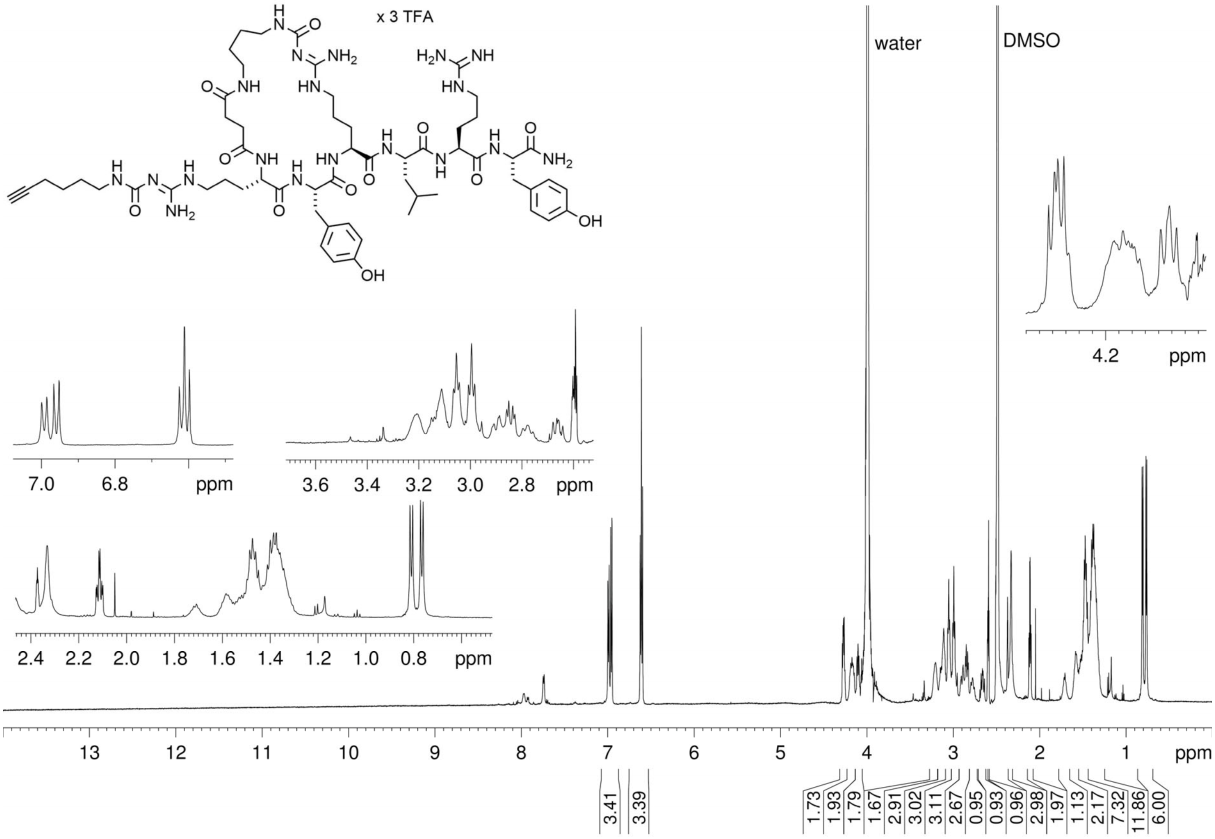

^{1}H-NMR spectrum (600 MHz, DMSO- d_6/D_2O 4:1 v/v) of compound **11**

6. References

1. Barnard, A., Long, K., Yeo, D. J., Miles, J. A., Azzarito, V., Burslem, G. M., Prabhakaran, P., A. Edwards, T., and Wilson, A. J. (2014) Orthogonal functionalisation of α-helix mimetics, *Org. Biomol. Chem. 12*, 6794-6799. DOI: 10.1039/C4OB00915K.
2. Wirth, U., Erl, J., Azzam, S., Höring, C., Skiba, M., Singh, R., Hochmuth, K., Keller, M., Wegener, J., and König, B. (2023) Monitoring the reversibility of GPCR signaling by combining photochromic ligands with label-free impedance analysis, *Angew. Chem. Int. Ed. 62*, e202215547. DOI: 10.1002/anie.202215547.
3. Gleixner, J., Gattor, A. O., Humphrys, L. J., Brunner, T., and Keller, M. (2023) [^{3}H]UR-JG102 - a radiolabeled cyclic peptide with high affinity and excellent selectivity for the neuropeptide Y Y_4 receptor, *J. Med. Chem. 66*, 13788-13808. DOI: 10.1021/acs.jmedchem.3c01224.
